# Selective Oxidation of Benzo[*d*]isothiazol-3(2*H*)-Ones Enabled by Selectfluor

**DOI:** 10.3390/molecules29163899

**Published:** 2024-08-17

**Authors:** Qin Li, Dan Yuan, Chong Liu, Faith Herington, Ke Yang, Haibo Ge

**Affiliations:** 1 Jiangsu Key Laboratory of Advanced Catalytic Materials & Technology, School of Petrochemical Engineering, Changzhou University, Changzhou 213164, China; s22020860048@smail.cczu.edu.cn (Q.L.); s23020860015@smail.cczu.edu.cn (D.Y.); 2 Department of Chemistry and Biochemistry, Texas Tech University, Lubbock, TX 79409, USA; chong.liu@ttu.edu (C.L.); fheringt@ttu.edu (F.H.)

**Keywords:** Selectfluor, oxidation, benzoisothiazol-3-one-1-oxide

## Abstract

A metal-free and Selectfluor-mediated selective oxidation reaction of benzo[*d*]isothiazol-3(2*H*)-ones in aqueous media is presented. This novel strategy provides a facile, green, and efficient approach to access important benzo[*d*]isothiazol-3(2*H*)-one-1-oxides with excellent yields and high tolerance to various functional groups. Furthermore, the purification of benzoisothiazol-3-one-1-oxides does not rely on column chromatography. Moreover, the preparation of saccharine derivatives has been achieved through sequential, double oxidation reactions in a one-pot aqueous media.

## 1. Introduction

**Scheme 1:**
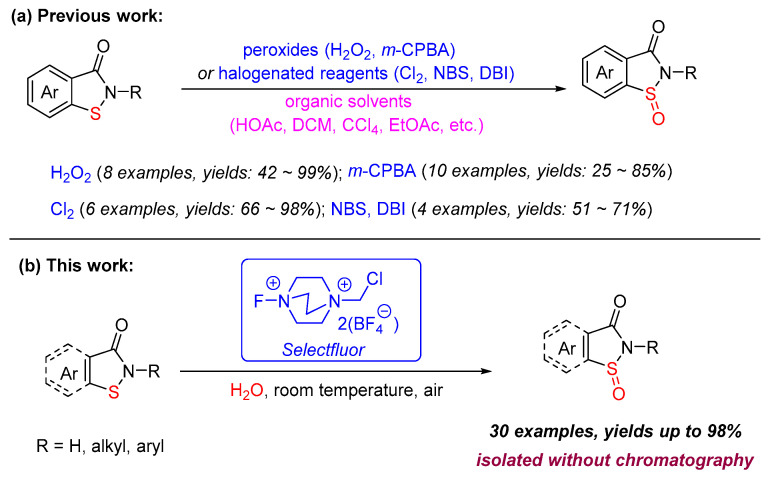
The direct oxidation of benzo[*d*]isothiazol-3(2*H*)-ones for benzoisothiazol-3-one-1-oxides.

Benzo[*d*]isothiazol-3(2*H*)-ones and their derivatives are widely used in medicine, agriculture, and the food industry [1,2,3,4,5,6,7]. Benzo[*d*]isothiazol-3(2*H*)-one-1-oxides, as important oxidative derivatives, have also attracted attention due to their promising biological properties, including antifungal, anxiolytic, and psychotropic activities [6,7]. As a result, significant efforts have been dedicated to the development of efficient methodologies [8,9,10]. The common method for synthesizing such skeletons involves the direct oxidation of benzo[*d*]isothiazol-3(2*H*)-ones. However, current oxidation approaches often require the use of unstable peroxides (H_2_O_2_ and *m*-CPBA) or toxic halogenated reagents (Cl_2_, NBS, and DBI). Moreover, the use of H_2_O_2_ and *m*-CPBA requires meticulous control of the oxidation temperature and reagent quantities. Notably, these methods are also constrained by their limited substrate scope, moderate yields, and considerable amounts of organic solvent usage (Scheme 1a) [10]. Therefore, seeking an ecofriendly, straightforward, and efficient method for constructing benzo[*d*]isothiazol-3(2*H*)-one-1-oxides would be of prime synthetic value.

In recent years, water has progressively been recognized as a green solvent in organic chemistry due to its advantages, such as its abundance, low cost, non-toxicity, and excellent chemical stability [11,12,13]. Meanwhile, Selectfluor [1-chloromethyl-4-fluoro-1,4-diazoniabicyclo-[2.2.2]octane bis(tetrafluoroborate)], a commercially available and exceptionally stable solid, possesses remarkable properties, including high thermal stability, excellent solubility and stability in water, as well as low toxicity [14]. Furthermore, Selectfluor not only serves as an electrophilic fluorinating reagent, but also functions as a remarkable oxidant in various organic transformations [15,16,17,18,19]. Therefore, utilizing water as the solvent and Selectfluor as the oxidant represents a sustainable strategy for achieving the direct oxidation of benzo[*d*]isothiazol-3(2*H*)-ones. As part of our continuous efforts in the field of sulfur chemistry [20,21], we herein report on the Selectfluor-mediated selective oxidation of benzo[*d*]isothiazol-3(2*H*)-ones in aqueous media to access benzo[*d*]isothiazol-3(2*H*)-one-1-oxides (Scheme 1b). In addition, the most famous sweetening agent, saccharine (benzo[*d*]isothiazol-3(2*H*)-one-1,1-dioxide) can also be prepared via one-pot, sequential, double oxidation reactions, using Selectfluor and *m*-CPBA in aqueous media.

## 2. Results and Discussion

**Scheme 2:**
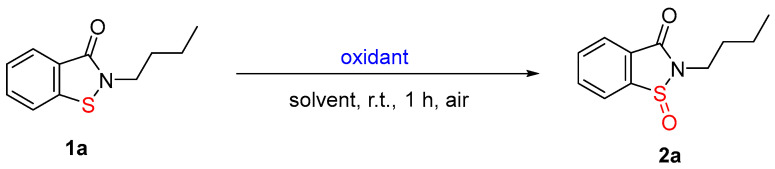
Selectfluor-mediated selective oxidation of 2-butylbenzo[*d*]isothiazol-3(2*H*)-one.

To begin our investigation, we explored the reaction of 2-butylbenzo[*d*]isothiazol-3(2*H*)-one (**1a**) with an oxidant at room temperature, in an ambient atmosphere (Scheme 2 and Table 1). To our delight, the desired product **2a** was obtained with an 87% NMR yield, using a water solvent and Selectfluor oxidant (Table 1, entry 1). We subsequently explored other organic solvents, including MeOH, EtOH, DMC, and DMF, with the latter yielding excellent results (>99% NMR yield) (Table 1, entries 2–5). After conducting a comprehensive screening of the oxidants (Selectfluor II, NFSI, NFTP, NIS, NaIO_4_, and K_2_S_2_O_8_), Selectfluor achieved unmatched results (Table 1, entries 6–11). We further enhanced the yield of aqueous **2a** by adding varying volumes of DMF, resulting in H_2_O/DMF (*v*/*v* = 9/1) being the optimal solvent ratio (Table 1, entries 12–14). It is important to note that **2a** was able to be isolated with a 95% yield without column chromatography purification. Finally, the investigation of differing Selectfluor amounts indicated that reducing its loading decreased the desired product yield, while increasing its loading had no impact on its reactivity (Table 1, entries 15–17).

**Scheme 3:**
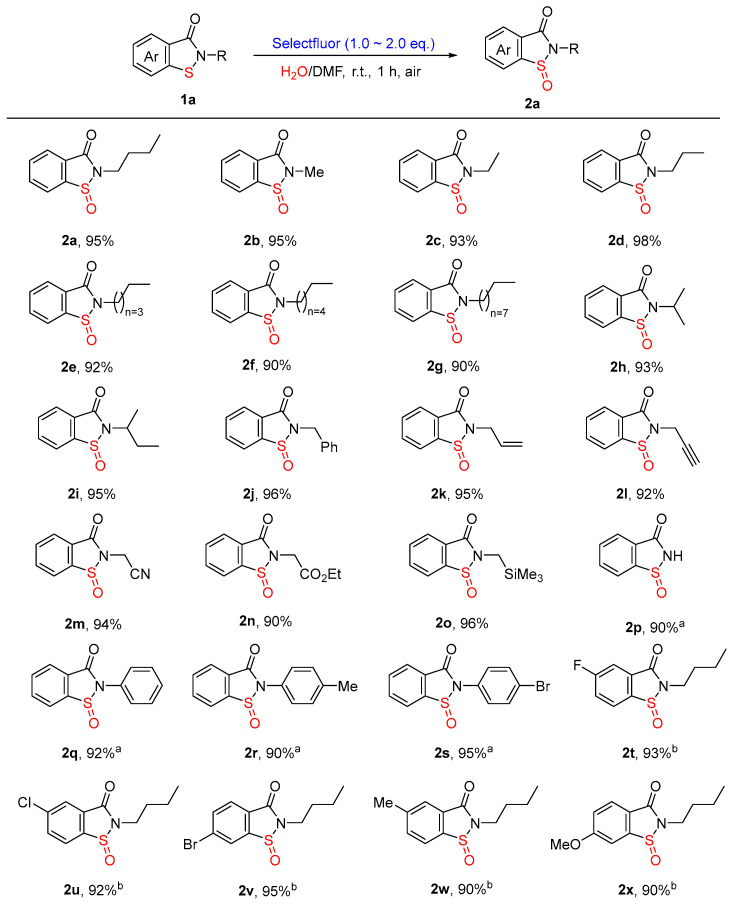
Scope of benzo[*d*]isothiazol-3(2*H*)-ones. Reaction conditions: **1a** (0.2 mmol), Selectfluor (0.2 mmol), H_2_O/DMF (2.0 mL, *v*/*v* = 9:1), room temperature (25 °C), 1 h, air. Isolated yields. ^a^ Selectfluor (0.4 mmol). ^b^ Selectfluor (0.3 mmol).

With the optimized conditions in hand, the substrate scope of benzo[*d*]isothiazol-3(2*H*)-ones was conducted. Various *N*-substituents on benzo[*d*]isothiazol-3(2*H*)-ones were initially tested, as depicted in Scheme 3. As anticipated, the introduction of different linear alkyl groups, including *n*-butyl, methyl, ethyl, *n*-propyl, *n*-amyl, *n*-hexyl, and *n*-nonyl, yielded the desired products **2a**–**g** in excellent yields. Next, both *iso*-propyl and *sec*-butyl substrates provided the products **2h** and **2i** with 93% and 95% yields, respectively. The benzyl substrate additionally resulted in the desired product **2j**, with a 96% yield. Gratifyingly, the substates **1k**–**o**, containing diverse functional groups, such as alkenyl, alkynyl, cyano, ester, and trimethylsilyl, exhibited excellent compatibility in this protocol, affording the desired products **2k**–**o** with high yields, ranging from 90% to 96%. The ability to convert these well-tolerated functional groups into other important moieties, further highlights the synthetic applicability of this protocol.

Subsequently, the N−H substrate **1p** was efficiently converted into product **2p**, with a yield of 90%, by using 2.0 equivalents of Selectfluor. Furthermore, the utilization of *N*-aryl substituents on benzo[*d*]isothiazol-3(2*H*)-ones resulted in excellent isolated yields (**2q**–**s**) when employing the same amount of Selectfluor. Finally, the presence of electron-withdrawing (F, Cl, Br) or electron-donating (Me, MeO) substituents on the phenyl ring at the 5- or 6- position of benzo[*d*]isothiazol-3(2*H*)-one was also compatible with the current reaction system, resulting in the isolation of desired products **2t**–**x**, with exceptional yields.

**Scheme 4:**
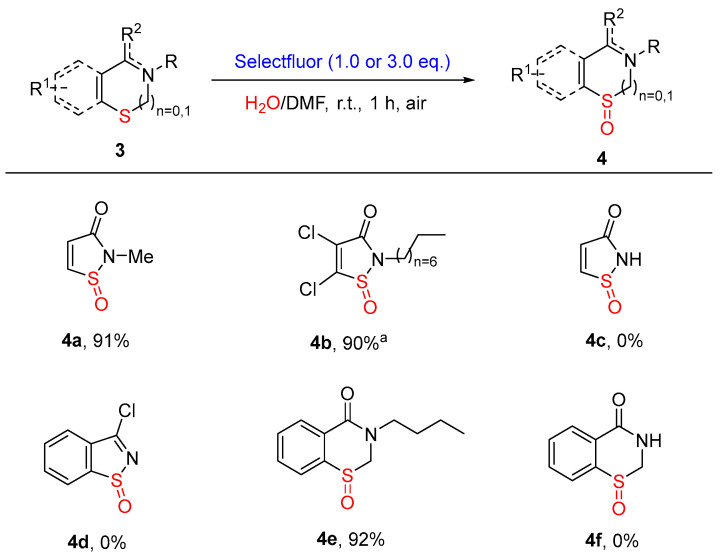
Scope of isothiazol-3-ones, isothiazoles, and benzothiazin-4-ones. Reaction conditions: **1a** (0.2 mmol), Selectfluor (0.2 mmol), H_2_O/DMF (2.0 mL, *v*/*v* = 9:1), room temperature (25 °C), 1 h, air. Isolated yields. ^a^ Selectfluor (0.6 mmol), 100 °C and 12 h.

A substrate scope of other similar sulfur-containing substrates was then studied (Scheme 4). The use of 2-methylisothiazol-3(2*H*)-one afforded the corresponding product **4a** with a 91% yield under standard conditions. Furthermore, the desired product **4b** was achieved with a yield of 90% by oxidizing 4,5-dichloro-2-octylisothiazol-3(2*H*)-one with 3.0 equivalents of Selectfluor at 100 °C for 12 h. Unfortunately, neither isothiazol-3(2*H*)-one nor 3-chlorobenzo[*d*]isothiazole provided the corresponding products **4c** and **4d**. Moreover, when benzothiazin-4-ones were employed, only the *N*-butyl substituted product **4e** was obtained with a 92% yield; additionally, displaying the unreliability of this strategy in isolating the 2-unsubstituted product **4f**.

**Scheme 5:**
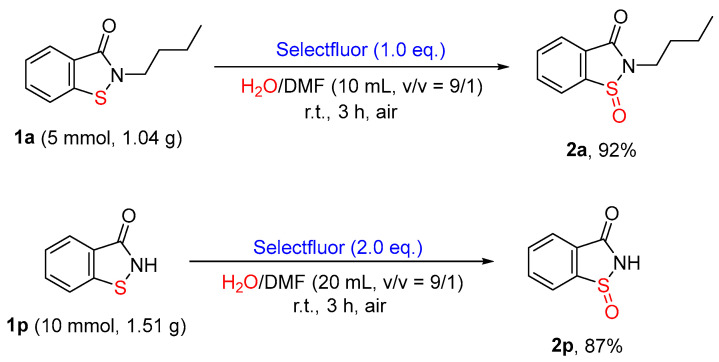
Gram-scale synthesis of benzo[*d*]isothiazol-3(2*H*)-one-1-oxides.

To demonstrate the synthetic usefulness of this approach, two gram-scale reactions were conducted for the synthesis of benzo[*d*]isothiazol-3(2*H*)-one-1-oxides (Scheme 5). By slightly modifying the conditions, both products **2a** and **2p** were successfully obtained with 92% and 87% yields, respectively, without employing traditional column chromatography purification methods.

**Scheme 6:**
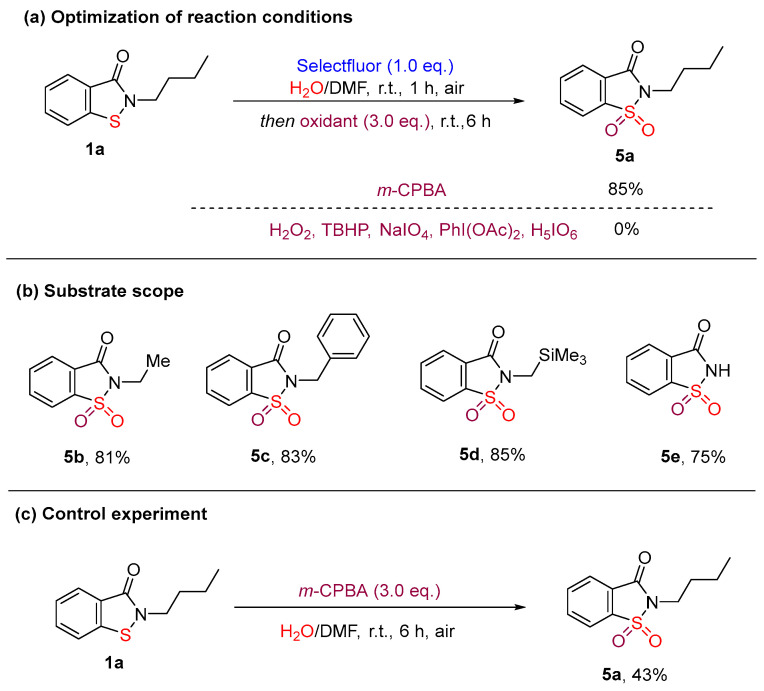
Sequential, one-pot, double oxidation reactions for the synthesis of saccharine derivatives.

Saccharine derivatives have garnered extensive interest due to their well-established role as non-caloric sweetening agents [22]. The previous approach for this skeleton mainly relied on the use of H_5_IO_6_ as an oxidant and CrO_3_ as a catalyst in regard to the MeCN solvent [23]. Inspired by the above results, we speculated that the addition of other oxidants (*m*-CPBA, H_2_O_2_, TBHP, NaIO_4_, PhI(OAc)_2_, and H_5_IO_6_) into the benzoisothiazol-3-one-1-oxide system could facilitate further oxidation for constructing saccharine derivatives. The screening results indicated that only *m*-CPBA was able to undergo sequential double oxidation in one-pot reactions, yielding the desired saccharine derivative **5a**, with an 85% yield (Scheme 6a). Additionally, different kinds of *N*-substituted benzo[*d*]isothiazol-3(2*H*)-ones were successfully converted into the desired saccharine derivatives **5b**–**e**, with good yields (Scheme 6b). Finally, the isolated yield of **5a** only reached 43% when exclusively employing *m*-CPBA (Scheme 6c).

**Scheme 7:**
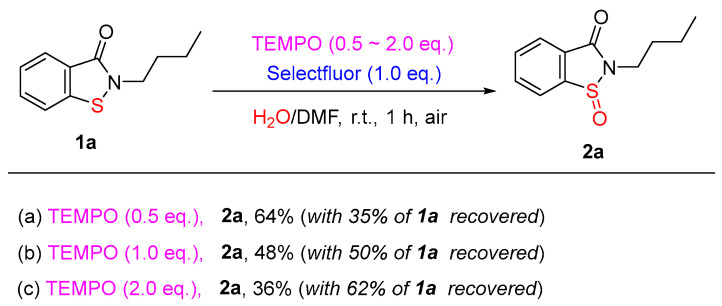
Free radical trapping experiments.

To further explore the reaction mechanism, we performed a series of free radical trapping experiments (Scheme 7). The addition of TEMPO (2,2,6,6-tetramethyl-1-piperidinyloxyl) suppressed the formation of product **2a**, leading to the recovery of starting material **1a**. These results demonstrate that Selectfluor may be utilized as a single-electron transfer (SET) oxidant in this process.

**Scheme 8:**
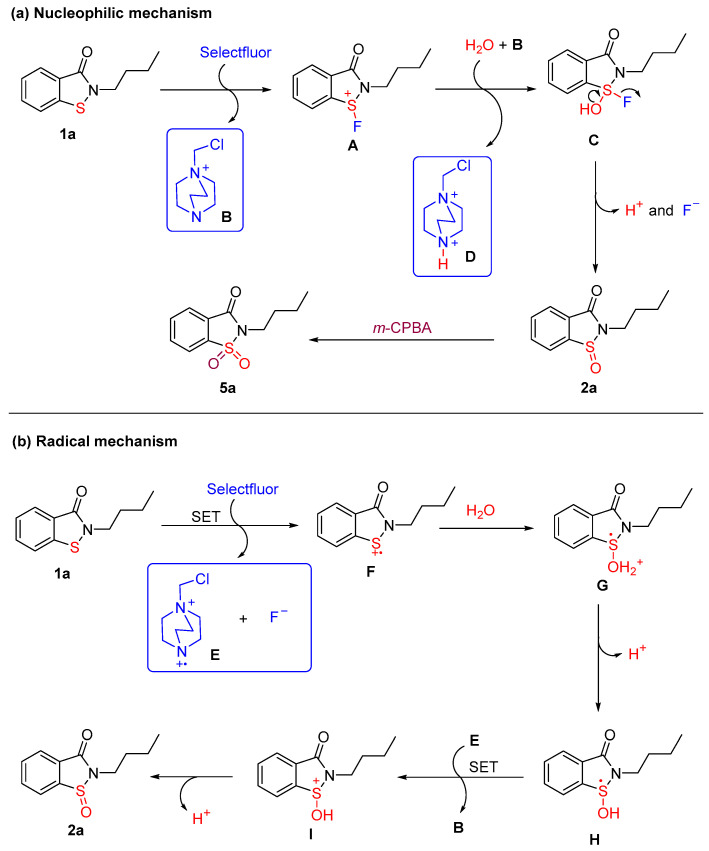
Proposed mechanism.

Based on the previous literature and control experiments, two plausible reaction mechanisms are proposed (Scheme 8). The nucleophilic mechanism involves the initial coordination of **1a** with Selectfluor, resulting in the formation of transient fluorosulfonium salt **A** and chloromethyl quaternary ammonium salt **B**. Next, salt **A** reacts with H_2_O and salt **B** to form intermediate **C** and salt **D**. Subsequently, the desired product **2a** is formed via the elimination of a hydrogen cation, along with a fluoride anion. Finally, *m*-CPBA oxidizes product **2a**, leading to the formation of the subsequent product **5a** (Scheme 8a) [20,24,25,26]. In addition, the presence of a radical pathway cannot be excluded at the present stage [27,28]. The single-electron transfer (SET) process between product **1a** and Selectfluor provides the nitrogen radical cation **E**, sulfur radical cation **F**, and fluoride anion. Subsequently, the sulfur radical cation **F** reacts with H_2_O to form the intermediate product **G**. Next, the deprotonation of **G**, followed by a second SET process with the nitrogen radical cation **E**, yields the cation intermediate **I**. This intermediate product **I** can then undergo deprotonation to yield the desired product **2a** (Scheme 8b).

## 3. Materials and Methods

### 3.1. General Information

All the solvents and commercially available reagents were purchased and used directly. Thin-layer chromatography (TLC) was performed on EMD precoated plates (silica gel 60 F254, Art 5715, Yantai Jiangyou Silica gel Development Co., Ltd., Yantai, China) and visualized by fluorescence quenching under UV light. Column chromatography was performed on EMD silica gel 60 (200–300 mesh, Shanghai Titan Technology Co., Ltd., Shanghai, China), using a forced flow of 0.5–1.0 bar. The ^1^H and ^13^C NMR spectra were obtained using a Bruker Avance III–300 or 400 spectrometer (Bruker Corporation, Billerica, MA, USA). ^1^H NMR data were reported as: chemical shift (δ ppm), multiplicity, coupling constant (Hz), and integration. ^13^C NMR data were reported in terms of the chemical shift (δ ppm), multiplicity, and coupling constant (Hz). Mass (HRMS) analysis was conducted using the Agilent 6200 Accurate-Mass TOF LC/MS system (Agilent Technologies Co., LTD, Santa Clara, CA, USA), with electrospray ionization (ESI). The melting points were measured by X4-A microscopic melting point apparatus (Shanghai INESA Physico-Optical Instrument Co., Ltd., Shanghai, China).

Benzo[*d*]isothiazol-3(2*H*)-ones (**1a**–**h**, **1j**–**n**, **1p**–**s**, **1u**–**x**), isothiazol-3(2*H*)-ones (**3a**–**c**), 3-chlorobenzo[*d*]isothiazole **3d**, and benzothiazin-4-one **3e** were purchased from Energy-chemical (Shanghai, China), BLDpharm (Shanghai, China), Chemieliva (Chongqing, China), Adamas-beta^®^ (Shanghai, China), TCI (Shanghai, China), J&K^@^ (Shanghai, China), or Sigma-Aldrich (Shanghai, China). Benzo[*d*]isothiazol-3(2*H*)-ones **1i** and **1o** were prepared by using benzo[d]isothiazol-3(2*H*)-one **1p** with 2-iodobutane and (iodomethyl)trimethylsilane, according to procedures in the literature [29]. Benzo[*d*]isothiazol-3(2*H*)-one **1t** was prepared by using *N*-butyl-5-fluoro-2-(methylthio)benzamide with Selectfluor, according to procedures in the literature [20]. Benzothiazin-4-ones **3f** was prepared by using *N*-butyl-2-(methylthio)benzamide with Selectfluor, according to procedures in the literature [21].

### 3.2. Optimization of the Reaction Conditions

A 25 mL ordinary tube was charged with 2-butylbenzo[*d*]isothiazol-3(2*H*)-one (**1a**, 41.46 mg, 0.2 mmol), a solvent (2.0 mL), and an additive (0.1–0.6 mmol). The tube was sealed, and the reaction was then stirred vigorously at room temperature (25 °C) for 1 h. After the reaction was finished, ethyl acetate (5 mL) was added. The organic phase was subjected to washing with H_2_O (2 × 5 mL) and brine (5 mL), followed by drying over Na_2_SO_4_ and filtration. The filtrate was concentrated in vacuo; the crude product was analyzed by ^1^H NMR in CDCl_3_. The yields are based on **1a**, determined by crude ^1^H NMR, using dibromomethane as the internal standard. The residue did not require further purification in order to obtain product **2a**.

### 3.3. Synthetic Procedures for the Synthesis of Compounds ***2***

A 25 mL ordinary tube was charged with *N*-substituted benzo[*d*]isothiazol-3(2*H*)-ones (**1a-o**, 0.2 mmol), Selectfluor (70.85 mg, 0.2 mmol), DMF (0.2 mL), and H_2_O (1.8 mL). The reaction was then stirred vigorously at room temperature for 1 h. After the reaction was finished, ethyl acetate (5 mL) was added. The organic phase was subjected to washing with H_2_O (2 × 5 mL) and brine (5 mL), followed by drying over Na_2_SO_4_ and filtration. The filtrate was concentrated in vacuo to yield the *N*-substituted benzo[*d*]isothiazol-3(2*H*)-one-1-oxides **2a**–**o**.

The details for *2-Butylbenzo[d]isothiazol-3(2H)-one-1-oxide* (**2a**). Colorless oil, 42.4 mg, 95%. ^1^H NMR (300 MHz, CDCl_3_) δ 7.94–7.91 (m, 1H), 7.83 (d, *J* = 7.3 Hz, 1H), 7.76–7.66 (m, 2H), 3.93–3.83 (m, 1H), 3.75–3.65 (m, 1H), 1.75–1.66 (m, 2H), 1.39–1.32 (m, 2H), 0.90 (t, *J* = 7.3 Hz, 3H). ^13^C NMR (75 MHz, CDCl_3_) δ 165.38, 145.56, 134.10, 133.21, 128.43, 126.11, 125.07, 41.07, 31.36, 20.09, 13.67. HRMS (ESI, *m*/*z*): calcd. for C_11_H_14_NO_2_S [M + H]^+^, 224.0740; found, 224.0737.

The details for *2-Methylbenzo[d]isothiazol-3(2H)-one-1-oxide* (**2b**). White solid, 34.4 mg, 95%, m.p. = 114–115 °C. ^1^H NMR (300 MHz, CDCl_3_) δ 8.01–7.99 (m, 1H), 7.93–7.90 (m, 1H), 7.84–7.73 (m, 2H), 3.39 (s, 3H). ^13^C NMR (75 MHz, CDCl_3_) δ 165.45, 145.47, 134.18, 133.27, 128.10, 126.07, 125.09, 26.98. HRMS (ESI, *m*/*z*): calcd. for C_8_H_8_NO_2_S [M + H]^+^, 182.0270; found, 182.0264.

The details for *2-Ethylbenzo[d]isothiazol-3(2H)-one-1-oxide* (**2c**). White solid, 36.3 mg, 93%, m.p. = 80–81 °C. ^1^H NMR (300 MHz, CDCl_3_) δ 8.02–7.99 (m, 1H), 7.93–7.89 (m, 1H), 7.83–7.73 (m, 2H), 4.09–3.97 (m, 1H), 3.91–3.79 (m, 1H), 1.42 (t, *J* = 7.2 Hz, 3H). ^13^C NMR (75 MHz, CDCl_3_) δ 165.14, 145.54, 134.12, 133.21, 128.47, 126.05, 125.07, 36.39, 14.81. HRMS (ESI, *m*/*z*): calcd. for C_9_H_10_NO_2_S [M + H]^+^, 196.0427; found, 196.0422.

The details for *2-Propylbenzo[d]isothiazol-3(2H)-one-1-oxide* (**2d**). White solid, 41.0 mg, 98%, m.p. = 55–56 °C. ^1^H NMR (300 MHz, CDCl_3_) δ 8.01–7.98 (m, 1H), 7.92–7.89 (m, 1H), 7.83–7.73 (m, 2H), 3.96–3.86 (m, 1H), 3.80–3.70 (m, 1H), 1.91–1.77 (m, 2H), 1.01 (t, *J* = 7.4 Hz, 3H). ^13^C NMR (75 MHz, CDCl_3_) δ 165.40, 145.55, 134.12, 133.21, 128.38, 126.10, 125.07, 42.93, 22.67, 11.37. HRMS (ESI, *m*/*z*): calcd. for C_10_H_12_NO_2_S [M + H]^+^, 210.0583; found, 210.0581.

The details for *2-Pentylbenzo[d]isothiazol-3(2H)-one-1-oxide* (**2e**). Colorless oil, 43.7 mg, 92%. ^1^H NMR (300 MHz, CDCl_3_) δ 7.91 (d, *J* = 7.1 Hz, 1H), 7.83 (d, *J* = 7.3 Hz, 1H), 7.75–7.65 (m, 2H), 3.91–3.82 (m, 1H), 3.73–3.63 (m, 1H), 1.78–1.68 (m, 2H), 1.37–1.23 (m, 4H), 0.83 (t, *J* = 7.2 Hz, 3H). ^13^C NMR (75 MHz, CDCl_3_) δ 165.34, 145.59, 134.07, 133.17, 128.43, 126.08, 125.05, 41.29, 28.99, 28.93, 22.23, 13.93. HRMS (ESI, *m*/*z*): calcd. for C_12_H_15_NNaO_2_S [M + Na]^+^, 260.0716; found, 260.0716.

The details for *2-Hexylbenzo[d]isothiazol-3(2H)-one 1-oxide* (**2f**). Colorless oil, 45.2 mg, 90%. ^1^H NMR (300 MHz, CDCl_3_) δ 7.92 (d, *J* = 7.2 Hz, 1H), 7.83 (d, *J* = 7.3 Hz, 1H), 7.75–7.65 (m, 2H), 3.92–3.79 (m, 1H), 3.73–3.63 (m, 1H), 1.78–1.67 (m, 2H), 1.35–1.18 (m, 6H), 0.81 (t, *J* = 6.7 Hz, 3H). ^13^C NMR (75 MHz, CDCl_3_) δ 165.35, 145.58, 134.08, 133.19, 128.44, 126.10, 125.06, 41.32, 31.37, 29.29, 26.51, 22.51, 14.02. HRMS (ESI, *m*/*z*): calcd. for C_13_H_17_NNaO_2_S [M + Na]^+^, 274.0872; found, 274.0866.

The details for *2-Nonylbenzo[d]isothiazol-3(2H)-one-1-oxide* (**2g**). Colorless oil, 52.8 mg, 90%. ^1^H NMR (300 MHz, CDCl_3_) δ 8.00 (dd, *J* = 6.9, 1.7 Hz, 1H), 7.91 (dd, *J* = 6.8, 1.6 Hz, 1H), 7.83–7.72 (m, 2H), 3.99–3.90 (m, 1H), 3.81–3.71 (m, 1H), 1.88–1.73 (m, 2H), 1.42–1.25 (m, 12H), 0.87 (t, *J* = 6.8 Hz, 3H). ^13^C NMR (75 MHz, CDCl_3_) δ 165.35, 145.60, 134.07, 133.18, 128.46, 126.11, 125.05, 41.33, 31.83, 29.44, 29.33, 29.22, 29.17, 26.85, 22.66, 14.12. HRMS (ESI, *m*/*z*): calcd. for C_16_H_23_NNaO_2_S [M + Na]^+^, 316.1342; found, 316.1337.

The details *2-Isopropylbenzo[d]isothiazol-3(2H)-one-1-oxide* (**2h**). White solid, 39.0 mg, 93%, m.p. = 47–48 °C. ^1^H NMR (300 MHz, CDCl_3_) δ 7.90 (d, *J* = 7.2 Hz, 1H), 7.81 (d, *J* = 7.3 Hz, 1H), 7.75–7.61 (m, 2H), 4.67–4.54 (m, 1H), 1.52–1.48 (m, 6H). ^13^C NMR (75 MHz, CDCl_3_) δ 165.29, 145.59, 134.07, 133.07, 128.74, 125.96, 124.87, 46.96, 22.49, 21.87. HRMS (ESI, *m*/*z*): calcd. for C_10_H_12_NO_2_S [M + H]^+^, 210.0583; found, 210.0583.

The details for *2-(sec-Butyl)benzo[d]isothiazol-3(2H)-one-1-oxide* (**2i**). Yellow oil, 42.4 mg, 95%. ^1^H NMR (300 MHz, CDCl_3_) δ 8.00–7.96 (m, 1H), 7.90–7.87 (m, 1H), 7.82–7.71 (m, 2H), 4.52–4.40 (m, 1H), 2.11–1.77 (m, 2H), 1.57–1.52 (m, 3H), 1.04–0.93 (m, 3H). ^13^C NMR (75 MHz, CDCl_3_) δ 165.56 (d, *J* = 10.3 Hz), 145.67 (d, *J* = 6.9 Hz), 134.05, 133.05 (d, *J* = 1.7 Hz), 128.68 (d, *J* = 7.7 Hz), 126.00 (d, *J* = 3.3 Hz), 124.87, 52.72 (d, *J* = 13.2 Hz), 29.00 (d, *J* = 38.6 Hz), 20.23 (d, *J* = 35.2 Hz), 11.14 (d, *J* = 3.9 Hz). HRMS (ESI, *m*/*z*): calcd. for C_11_H_14_NO_2_S [M + H]^+^, 224.0740; found, 224.0738.

The details for *2-Benzylbenzo[d]isothiazol-3(2H)-one-1-oxide* (**2j**). White solid, 49.4 mg, 96%, m.p. = 92–93 °C (known compound [30]). ^1^H NMR (300 MHz, CDCl_3_) δ 7.93–7.90 (m, 1H), 7.79 (d, *J* = 7.2 Hz, 1H), 7.72–7.62 (m, 2H), 7.35–7.18 (m, 5H), 5.20 (d, *J* = 15.3 Hz, 1H), 4.65 (d, *J* = 15.3 Hz, 1H). ^13^C NMR (75 MHz, CDCl_3_) δ 165.22, 145.64, 135.83, 134.28, 133.29, 128.89, 128.65, 128.24, 128.22, 126.31, 125.24, 44.33.

The details for *2-Allylbenzo[d]isothiazol-3(2H)-one-1-oxide* (**2k**). Colorless oil, 39.4 mg, 95% (known compound [30]). ^1^H NMR (300 MHz, CDCl_3_) δ 7.94 (d, *J* = 7.2 Hz, 1H), 7.84 (d, *J* = 7.4 Hz, 1H), 7.77–7.66 (m, 2H), 5.95–5.82 (m, 1H), 5.31 (d, *J* = 17.0 Hz, 1H), 5.24 (d, *J* = 10.1 Hz, 1H), 4.62–4.54 (m, 1H), 4.22 (dd, *J* = 15.9, 6.8 Hz, 1H). ^13^C NMR (75 MHz, CDCl_3_) δ 165.11, 145.68, 134.26, 133.26, 131.73, 128.20, 126.24, 125.19, 119.34, 43.17.

The details for *2-(Prop-2-yn-1-yl)benzo[d]isothiazol-3(2H)-one-1-oxide* (**2l**). White solid, 37.8 mg, 92%, m.p. = 142–143 °C. ^1^H NMR (300 MHz, CDCl_3_) δ 7.99–7.96 (m, 1H), 7.85–7.82 (m, 1H), 7.75–7.65 (m, 2H), 5.29–5.16 (m, 2H), 2.67 (t, *J* = 2.5 Hz, 1H). ^13^C NMR (75 MHz, CDCl_3_) δ 169.98, 154.81, 132.73, 132.39, 129.33, 125.05, 124.27, 77.00, 76.30, 57.79. HRMS (ESI, *m*/*z*): calcd. for C_10_H_7_NNaO_2_S [M + Na]^+^, 228.0090; found, 228.0090.

The details for *2-(1-Oxido-3-oxobenzo[d]isothiazol-2(3H)-yl)acetonitrile* (**2m**). White solid, 38.7 mg, 94%, m.p. = 120–121 °C. ^1^H NMR (300 MHz, CDCl_3_) *δ* 7.98 (d, *J* = 7.4 Hz, 1H), 7.90 (d, *J* = 7.5 Hz, 1H), 7.85–7.72 (m, 2H), 4.84 (d, *J* = 17.9 Hz, 1H), 4.50 (d, *J* = 17.9 Hz, 1H). ^13^C NMR (75 MHz, CDCl_3_) δ 164.57, 145.53, 135.26, 133.86, 126.79, 126.61, 125.69, 114.17, 28.04. HRMS (ESI, *m*/*z*): calcd. for C_9_H_7_N_2_O_2_S [M + H]^+^, 207.0223; found, 207.0223.

The details for *Ethyl 2-(1-oxido-3-oxobenzo[d]isothiazol-2(3H)-yl)acetate* (**2n**). Colorless oil, 45.5 mg, 90%, (known compound [31]). ^1^H NMR (300 MHz, CDCl_3_) δ 7.89 (d, *J* = 6.8 Hz, 1H), 7.79 (d, *J* = 7.9 Hz, 1H), 7.69–7.58 (m, 2H), 5.13 (d, *J* = 15.7 Hz, 1H), 4.97 (d, *J* = 15.7 Hz, 1H), 4.22 (q, *J* = 7.2 Hz, 2H), 1.23 (t, *J* = 7.2 Hz, 3H). ^13^C NMR (75 MHz, CDCl_3_) δ 170.23, 166.54, 154.91, 132.75, 132.38, 129.12, 125.02, 124.37, 65.61, 61.90, 14.11.

The details for *2-((Trimethylsilyl)methyl)benzo[d]isothiazol-3(2H)-one-1-oxide* (**2o**). Colorless oil, 48.7 mg, 96%. ^1^H NMR (300 MHz, CDCl_3_) δ 7.96–7.93 (m, 1H), 7.88–7.83 (m, 1H), 7.78–7.69 (m, 2H), 3.43 (d, *J* = 15.6 Hz, 1H), 3.19 (d, *J* = 15.6 Hz, 1H), 0.16 (s, 9H). ^13^C NMR (75 MHz, CDCl_3_) δ 165.22, 145.49, 133.86, 133.21, 128.40, 125.93, 124.97, 32.13, -1.63. HRMS (ESI, *m*/*z*): calcd. for C_11_H_16_NO_2_SSi [M + H]^+^, 254.0666; found, 254.0666.

A 25 mL ordinary tube was charged with *N*-substituted benzo[*d*]isothiazol-3(2*H*)-ones (**1p**–**s**, 0.2 mmol), Selectfluor (141.70 mg, 0.4 mmol), DMF (0.2 mL), and H_2_O (1.8 mL). The reaction was then stirred vigorously at room temperature for 1 h. After the reaction was finished, ethyl acetate (5 mL) was added. The organic phase was subjected to washing with H_2_O (2 × 5 mL) and brine (5 mL), followed by drying over Na_2_SO_4_ and filtration. The filtrate was concentrated in vacuo to yield the *N*-substituted benzo[*d*]isothiazol-3(2*H*)-one-1-oxides **2p**–**s**.

The details for *Benzo[d]isothiazol-3(2H)-one-1-oxide* (**2p**). White solid, 30.1 mg, 90%, m.p. = 158–159 °C (known compound [30]). ^1^H NMR (300 MHz, DMSO-*d_6_*) δ 11.52 (br, 1H), 8.13 (d, *J* = 7.5 Hz, 1H), 7.95–7.82 (m, 3H). ^13^C NMR (75 MHz, DMSO-*d_6_*) δ 167.94, 148.57, 135.18, 133.69, 127.73, 126.33, 125.91.

The details for *2-Phenylbenzo[d]isothiazol-3(2H)-one-1-oxide* (**2q**). White solid, 44.8 mg, 92%, m.p. = 136–137 °C (known compound [30]). ^1^H NMR (400 MHz, CDCl_3_) δ 8.11–8.09 (m, 1H), 7.98–7.95 (m, 1H), 7.89–7.79 (m, 2H), 7.53–7.43 (m, 5H). ^13^C NMR (101 MHz, CDCl_3_) δ 164.54, 145.51, 134.65, 133.90, 133.48, 129.79, 128.94, 128.25, 127.42, 126.80, 125.26.

The details for *2-(p-Tolyl)benzo[d]isothiazol-3(2H)-one-1-oxide* (**2r**). White solid, 46.3 mg, 90%, m.p. = 169–170 °C (known compound [30]). ^1^H NMR (400 MHz, CDCl_3_) δ 8.10–8.07 (m, 1H), 7.97–7.94 (m, 1H), 7.88–7.78 (m, 2H), 7.41–7.38 (m, 2H), 7.32–7.30 (m, 2H), 2.41 (s, 3H). ^13^C NMR (101 MHz, CDCl_3_) δ 164.66, 145.57, 139.19, 134.57, 133.42, 131.04, 130.42, 128.29, 127.45, 126.73, 125.24, 21.31.

The details for *2-(4-Bromophenyl)benzo[d]isothiazol-3(2H)-one-1-oxide* (**2s**). White solid, 61.2 mg, 95%, m.p. = 100–101 °C. ^1^H NMR (400 MHz, CDCl_3_) δ 8.11–8.09 (m, 1H), 7.98–7.96 (m, 1H), 7.91–7.81 (m, 2H), 7.66–7.62 (m, 2H), 7.45–7.41 (m, 2H). ^13^C NMR (101 MHz, CDCl_3_) δ 164.37, 145.39, 134.81, 133.60, 133.10, 132.94, 128.74, 128.02, 126.87, 125.30, 122.81. HRMS (ESI, *m*/*z*): calcd. for C_13_H_9_BrNO_2_S [M + H]^+^, 321.9532; found, 321.9529.

A 25 mL ordinary tube was charged with *N*-substituted benzo[*d*]isothiazol-3(2*H*)-ones (**1t**–**x**, 0.2 mmol), Selectfluor (106.28 mg, 0.3 mmol), DMF (0.2 mL), and H_2_O (1.8 mL). The reaction was then stirred vigorously at room temperature for 1 h. After the reaction was finished, ethyl acetate (5 mL) was added. The organic phase was subjected to washing with H_2_O (2 × 5 mL) and brine (5 mL), followed by drying over Na_2_SO_4_ and filtration. The filtrate was concentrated in vacuo to yield the *N*-substituted benzo[*d*]isothiazol-3(2*H*)-one-1-oxides **2t**–**x**.

The details for *2-Butyl-5-fluorobenzo[d]isothiazol-3(2H)-one-1-oxide* (**2t**). White solid, 45.0 mg, 93%, m.p. = 82–83 °C. ^1^H NMR (300 MHz, CDCl_3_) δ 7.83 (dd, *J* = 8.5, 4.3 Hz, 1H), 7.58 (dd, *J* = 7.4, 2.4 Hz, 1H), 7.44–7.37 (m, 1H), 3.91–3.82 (m, 1H), 3.74–3.64 (m, 1H), 1.76–1.66 (m, 2H), 1.41–1.29 (m, 2H), 0.89 (t, *J* = 7.4 Hz, 3H). ^13^C NMR (75 MHz, CDCl_3_) δ 165.65 (d, *J* = 256.5 Hz), 164.12 (d, *J* = 3.0 Hz), 141.09 (d, *J* = 3.1 Hz), 131.59 (d, *J* = 9.2 Hz), 127.33 (d, *J* = 9.3 Hz), 121.45 (d, *J* = 23.9 Hz), 113.32 (d, *J* = 24.5 Hz), 41.33, 31.25, 20.04, 13.60. ^19^F NMR (282 MHz, CDCl_3_) δ -103.42. HRMS (ESI, *m*/*z*): calcd. for C_11_H_13_FNO_2_S [M + H]^+^, 242.0646; found, 242.0648.

The details for *2-Butyl-5-chlorobenzo[d]isothiazol-3(2H)-one-1-oxide* (**2u**). Colorless oil, 47.4 mg, 92%. ^1^H NMR (300 MHz, CDCl_3_) δ 7.88 (dd, *J* = 1.9, 0.5 Hz, 1H), 7.77 (dd, *J* = 8.2, 0.6 Hz, 1H), 7.68 (dd, *J* = 8.2, 1.9 Hz, 1H), 3.91–3.81 (m, 1H), 3.74–3.64 (m, 1H), 1.76–1.65 (m, 2H), 1.41–1.29 (m, 2H), 0.90 (t, *J* = 7.3 Hz, 3H). ^13^C NMR (75 MHz, CDCl_3_) δ 164.14, 143.64, 140.08, 134.12, 130.37, 126.29, 126.27, 41.31, 31.26, 20.05, 13.61. HRMS (ESI, *m*/*z*): calcd. for C_11_H_13_ClNO_2_S [M + H]^+^, 258.0350; found, 258.0343.

The details for *6-Bromo-2-butylbenzo[d]isothiazol-3(2H)-one-1-oxide* (**2v**). White solid, 57.4 mg, 95%, m.p. = 97–98 °C. ^1^H NMR (300 MHz, CDCl_3_) δ 8.04 (s, 1H), 7.90–7.83 (m, 1H), 3.98–3.88 (m, 1H), 3.81–3.72 (m, 1H), 1.86–1.73 (m, 2H), 1.48–1.36 (m, 2H), 0.97 (t, *J* = 7.3 Hz, 3H). ^13^C NMR (75 MHz, CDCl_3_) δ 164.61, 147.12, 136.50, 128.84, 128.31, 127.35, 127.20, 41.24, 31.27, 20.06, 13.61. HRMS (ESI, *m*/*z*): calcd. for C_11_H_13_BrNO_2_S [M + H]^+^, 301.9845; found, 301.9840.

The details for *2-Butyl-5-methylbenzo[d]isothiazol-3(2H)-one-1-oxide* (**2w**). White solid, 42.7 mg, 90%, m.p. = 100–101 °C. ^1^H NMR (300 MHz, CDCl_3_) δ 7.71–7.68 (m, 2H), 7.51 (d, *J* = 7.8 Hz, 1H), 3.91–3.81 (m, 1H), 3.72–3.62 (m, 1H), 2.45 (s, 3H), 1.76–1.65 (m, 2H), 1.41–1.29 (m, 2H), 0.89 (t, *J* = 7.3 Hz, 3H). ^13^C NMR (75 MHz, CDCl_3_) δ 165.50, 144.33, 142.77, 134.75, 128.68, 126.37, 124.84, 41.03, 31.35, 21.67, 20.08, 13.64. HRMS (ESI, *m*/*z*): calcd. for C_12_H_15_NNaO_2_S [M + Na]^+^, 260.0716; found, 260.0716.

The details for *2-Butyl-6-methoxybenzo[d]isothiazol-3(2H)-one-1-oxide* (**2x**). White solid, 45.6 mg, 90%, m.p. = 139–140 °C. ^1^H NMR (300 MHz, CDCl_3_) δ 7.89 (d, *J* = 8.4 Hz, 1H), 7.36 (s, 1H), 7.21 (dd, *J* = 8.5, 2.2 Hz, 1H), 3.95–3.87 (m, 4H), 3.79–3.70 (m, 1H), 1.83–1.72 (m, 2H), 1.46– 1.39 (m, 2H), 0.97 (t, *J* = 7.3 Hz, 3H). ^13^C NMR (75 MHz, CDCl_3_) δ 165.21, 164.54, 147.86, 127.43, 120.44, 119.56, 109.51, 56.19, 41.08, 31.42, 20.07, 13.64. HRMS (ESI, *m*/*z*): calcd. for C_12_H_16_NO_3_S [M + H]^+^, 254.0845; found, 254.0666.

### 3.4. Synthetic Procedures for the Synthesis of Compounds ***4***

A 25 mL ordinary tube was charged with 2-methylisothiazol-3(2*H*)-one (**3a**, 23.03 mg, 0.2 mmol), Selectfluor (70.85 mg, 0.2 mmol), DMF (0.2 mL), and H_2_O (1.8 mL). The reaction was then stirred vigorously at room temperature for 1 h. After the reaction was finished, ethyl acetate (5 mL) was added. The organic phase was subjected to washing with H_2_O (2 × 5 mL) and brine (5 mL), followed by drying over Na_2_SO_4_ and filtration. The filtrate was concentrated in vacuo to yield the product **4a**.

The details for *2-Methylisothiazol-3(2H)-one-1-oxide* (**4a**). White solid, 24.0 mg, 91%, m.p. = 81–82 °C. ^1^H NMR (300 MHz, CDCl_3_) δ 7.60 (d, *J* = 6.5 Hz, 1H), 6.83 (d, *J* = 6.4 Hz, 1H), 3.25 (s, 3H). ^13^C NMR (75 MHz, CDCl_3_) δ 166.06, 148.19, 130.66, 26.57. HRMS (ESI, *m*/*z*): calcd. for C_4_H_6_NO_2_S [M + H]^+^, 132.0114; found, 132.0111.

A 25 mL ordinary tube was charged with 4,5-dichloro-2-octylisothiazol-3(2*H*)-one (**3b**, 56.44 mg, 0.2 mmol), Selectfluor (212.56 mg, 0.6 mmol), DMF (0.2 mL), and H_2_O (1.8 mL). The reaction was then stirred vigorously at 100 °C for 12 h. After the reaction was finished, ethyl acetate (5 mL) was added. The organic phase was subjected to washing with H_2_O (2 × 5 mL) and brine (5 mL), followed by drying over Na_2_SO_4_ and filtration. The filtrate was concentrated in vacuo to yield the product **4b**.

The details for *4,5-Dichloro-2-octylisothiazol-3(2H)-one-1-oxide* (**4b**). Colorless oil, 53.7 mg, 90%. ^1^H NMR (300 MHz, CDCl_3_) δ 3.80–3.59 (m, 2H), 1.71–1.62 (m, 2H), 1.31–1.18 (m, 10H), 0.81 (d, *J* = 6.6 Hz, 3H). ^13^C NMR (75 MHz, CDCl_3_) δ 159.92, 148.68, 130.86, 42.85, 31.72, 29.08, 29.07, 29.00, 26.66, 22.60, 14.07. HRMS (ESI, *m*/*z*): calcd. for C_11_H_18_Cl_2_NO_2_S [M + H]^+^, 298.0430; found, 298.0416.

A 25 mL ordinary tube was charged with 3-butyl-2,3-dihydro-4*H*-benzo[*e*][1,3]thiazin-4-one (**3e**, 44.26 mg, 0.2 mmol), Selectfluor (70.85 mg, 0.2 mmol), DMF (0.2 mL), and H_2_O (1.8 mL). The reaction was then stirred vigorously at room temperature for 1 h. After the reaction was finished, ethyl acetate (5 mL) was added. The organic phase was subjected to washing with H_2_O (2 × 5 mL) and brine (5 mL), followed by drying over Na_2_SO_4_ and filtration. The filtrate was concentrated in vacuo to yield the product **4e**.

The details for *3-Butyl-2,3-dihydro-4H-benzo[e][1,3]thiazin-4-one-1-oxide* (**4e**). Colorless oil, 43.7 mg, 92%. ^1^H NMR (300 MHz, CDCl_3_) δ 8.14–8.11 (m, 1H), 7.70–7.56 (m, 3H), 4.67 (d, *J* = 13.0 Hz, 1H), 4.48 (d, *J* = 13.0 Hz, 1H), 3.61 (t, *J* = 7.4 Hz, 2H), 1.64–1.54 (m, 2H), 1.39–1.28 (m, 2H), 0.89 (t, *J* = 7.4 Hz, 3H). ^13^C NMR (75 MHz, CDCl_3_) δ 162.05, 140.88, 132.84, 132.56, 130.42, 127.51, 127.00, 65.22, 48.87, 29.80, 19.95, 13.77. HRMS (ESI, *m*/*z*): calcd. for C_12_H_15_NNaO_2_S [M + Na]^+^, 260.0716; found, 260.0714.

### 3.5. Synthetic Procedures for Gram-Scale Reactions

A 50 mL round-bottom flask was charged with 2-butylbenzo[*d*]isothiazol-3(2*H*)-one (**1a**, 1.04 g, 5 mmol), Selectfluor (1.77 g, 5 mmol), DMF (2 mL), and H_2_O (18 mL). The reaction was then stirred vigorously at room temperature for 3 h. After the reaction was finished, ethyl acetate (20 mL) was added. The organic phase was subjected to washing with H_2_O (2 × 15 mL) and brine (15 mL), followed by drying over Na_2_SO_4_ and filtration. The filtrate was concentrated in vacuo to yield the 2-butylbenzo[*d*]isothiazol-3(2*H*)-one-1-oxide **2a** (1.03 g, 92%).

A 50 mL round-bottom flask was charged with benzo[*d*]isothiazol-3(2*H*)-one (**1p**, 1.51 g, 10 mmol), Selectfluor (7.09 g, 20 mmol), DMF (3 mL), and H_2_O (27 mL). The reaction was then stirred vigorously at room temperature for 3 h. After the reaction was finished, ethyl acetate (40 mL) was added. The organic phase was subjected to washing with H_2_O (2 × 25 mL) and brine (25 mL), followed by drying over Na_2_SO_4_ and filtration. The filtrate was concentrated in vacuo to yield the benzo[*d*]isothiazol-3(2*H*)-one-1-oxide **2p** (1.45 g, 87%).

### 3.6. Synthetic Procedures for the Synthesis of Compounds ***5***

A 25 mL ordinary tube was charged with *N*-substituted benzo[*d*]isothiazol-3(2*H*)-ones (**1**, 0.2 mmol), Selectfluor (70.85 mg, 0.2 mmol), DMF (0.2 mL), and H_2_O (1.8 mL). The reaction was then stirred vigorously at room temperature for 1 h. Next, *m*-CPBA (103.54 mg, 0.6 mmol) was added to the reaction system, then stirred vigorously at room temperature for 6 h. After the reaction was finished, ethyl acetate (5 mL) was added. The organic phase was treated with NaOH aqueous solution (10 wt%), followed by washing with H_2_O (2 × 5 mL) and brine (5 mL), then dried over Na_2_SO_4_ and filtered. The filtrate was concentrated in vacuo to yield the *N*-substituted benzo[*d*]isothiazol-3(2*H*)-one-1,1-dioxides **5a**–**d**.

The details for *2-Butylbenzo[d]isothiazol-3(2H)-one-1,1-dioxide* (**5a**). White solid, 40.7 mg, 85%, m.p. = 42–43 °C (known compound [32]). ^1^H NMR (300 MHz, CDCl_3_) δ 8.00–7.96 (m, 1H), 7.87–7.72 (m, 3H), 3.70 (t, *J* = 7.4 Hz, 2H), 1.82–1.72 (m, 2H), 1.44–1.31 (m, 2H), 0.91 (t, *J* = 7.4 Hz, 3H). ^13^C NMR (75 MHz, CDCl_3_) δ 158.96, 137.74, 134.65, 134.27, 127.48, 125.09, 120.88, 39.23, 30.43, 20.05, 13.52.

The details for *2-Ethylbenzo[d]isothiazol-3(2H)-one-1,1-dioxide* (**5b**). White solid, 34.2 mg, 81%, m.p. = 93–94 °C (known compound [32]). ^1^H NMR (300 MHz, CDCl_3_) δ 7.99–7.96 (m, 1H), 7.86–7.72 (m, 3H), 3.78 (q, *J* = 7.2 Hz, 2H), 1.38 (t, *J* = 7.2 Hz, 3H). ^13^C NMR (75 MHz, CDCl3) δ 158.71, 137.78, 134.67, 134.29, 127.50, 125.08, 120.88, 34.50, 13.98.

The details for *2-Benzylbenzo[d]isothiazol-3(2H)-one-1,1-dioxide* (**5c**). White solid, 45.4 mg, 83%, m.p. = 110–111 °C (known compound [32]). ^1^H NMR (300 MHz, CDCl_3_) δ 7.97–7.94 (m, 1H), 7.85–7.69 (m, 3H), 7.42 (dd, *J* = 7.6, 1.9 Hz, 2H), 7.30–7.17 (m, 3H), 4.82 (s, 2H). ^13^C NMR (75 MHz, CDCl_3_) δ 158.93, 137.75, 134.85, 134.51, 134.38, 128.75, 128.72, 128.30, 127.30, 125.26, 121.06, 42.69.

The details for *2-((Trimethylsilyl)methyl)benzo[d]isothiazol-3(2H)-one-1,1-dioxide* (**5d**). Colorless oil, 45.8 mg, 85% (known compound [33]). ^1^H NMR (300 MHz, CDCl_3_) δ 8.03–8.00 (m, 1H), 7.92–7.89 (m, 1H), 7.86–7.77 (m, 2H), 3.13 (s, 2H), 0.19 (s, 9H). ^13^C NMR (75 MHz, CDCl_3_) δ 159.02, 137.75, 134.45, 134.33, 127.79, 124.98, 120.99, 29.12, -1.54.

A 25 mL ordinary tube was charged with **1p** (30.24 mg, 0.2 mmol), Selectfluor (141.70 mg, 0.4 mmol), DMF (0.2 mL), and H_2_O (1.8 mL). The reaction was then stirred vigorously at room temperature for 1 h. Next, *m*-CPBA (103.54 mg, 0.6 mmol) was added to the reaction system, then stirred vigorously at room temperature for 6 h. After the reaction was finished, ethyl acetate (5 mL) was added. The organic phase was treated with NaOH aqueous solution (10 wt%), followed by washing with H_2_O (2 × 5 mL) and brine (5 mL), then dried over Na_2_SO_4_ and filtered. The filtrate was concentrated in vacuo, the residue was purified by flash chromatography on silica gel, using EtOAc/MeOH (*v*/*v* = 10/1) as the eluent, to yield the product **5e**.

The details for *Benzo[d]isothiazol-3(2H)-one-1,1-dioxide* (**5e**). White solid, 27.5 mg, 75%, m.p. = 227–228 °C (known compound [34]). ^1^H NMR (300 MHz, DMSO-*d*_6_) δ 12.36 (br, 1H), 8.19–8.15 (m, 1H), 8.04–7.92 (m, 3H). ^13^C NMR (75 MHz, DMSO*-d*_6_) δ 161.30, 139.74, 136.01, 135.22, 127.92, 125.31, 121.64.

### 3.7. Procedures for Free Radical Trapping Experiments

A 25 mL ordinary tube was charged with 2-butylbenzo[*d*]isothiazol-3(2*H*)-one (**1a**, 41.46 mg, 0.2 mmol), Selectfluor (141.70 mg, 0.4 mmol), DMF (0.2 mL), H_2_O (1.8 mL), and TEMPO (0.1, 0.2, or 0.4 mmol). The tube was sealed, and the reaction was then stirred vigorously at room temperature for 1 h. After the reaction was finished, ethyl acetate (5 mL) was added. The organic phase was subjected to washing with H_2_O (2 × 5 mL) and brine (5 mL), followed by drying over Na_2_SO_4_ and filtration. The filtrate was concentrated in vacuo, then the crude product was analyzed by ^1^H NMR in CDCl_3_. The yields are based on **1a**, determined by crude ^1^H NMR, using dibromomethane as the internal standard.

## 4. Conclusions

In summary, we have developed Selectfluor-mediated, selective oxidation of benzo[*d*]isothiazol-3(2*H*)-ones, in the aqueous phase. This strategy demonstrated broad tolerance towards assorted functional groups, producing a variety of benzo[*d*]isothiazol-3(2*H*)-one-1-dioxides with excellent yields, without column chromatography purification. Furthermore, other similar sulfur-containing substrates, including *N*-substituted isothiazol-3(2*H*)-ones and benzothiazin-4-ones, proved suitable for this strategy. Lastly, saccharine derivatives were also synthesizable via sequential, one-pot, double oxidation using Selectfluor and *m*-CPBA in aqueous media.

## Figures and Tables

**Table 1:** Optimization of reaction conditions ^a^.

Entry	Oxidant (Eq.)	Solvent (*v*/*v*, mL)	Yield (%)
1	Selectfluor (1.0)	H_2_O	87
2	Selectfluor (1.0)	MeOH	trace
3	Selectfluor (1.0)	EtOH	0
4	Selectfluor (1.0)	DMC	0
5	Selectfluor (1.0)	DMF	>99
6	Selectfluor II (1.0)	H_2_O	82
7	NFSI (1.0)	H_2_O	80
8	NFTP (1.0)	H_2_O	0
9	NIS (1.0)	H_2_O	0
10	NaIO_4_ (1.0)	H_2_O	0
11	K_2_S_2_O_8_ (1.0)	H_2_O	0
12	Selectfluor (1.0)	H_2_O/DMF (*v*/*v* = 4/1)	>99
13	Selectfluor (1.0)	H_2_O/DMF (*v*/*v* = 9/1)	>99[95] ^b^
14	Selectfluor (1.0)	H_2_O/DMF (*v*/*v* = 19/1)	90
15	Selectfluor (0.5)	H_2_O/DMF (*v*/*v* = 9/1)	48
16	Selectfluor (2.0)	H_2_O/DMF (*v*/*v* = 9/1)	>99
17	Selectfluor (3.0)	H_2_O/DMF (*v*/*v* = 9/1)	>99

^a^ Reaction conditions: **1a** (0.2 mmol), oxidant (0.1–0.6 mmol), solvent (2.0 mL), room temperature (25 °C), 1 h, air. Yields are based on **1a**, determined by ^1^H-NMR using dibromomethane as the internal standard. ^b^ Isolated yields. Selectfluor =1-chloromethyl-4-fluoro-1,4-diazoniabicyclo [2.2.2]octane bis(tetrafluoroborate). Selectfluor II = 1-fluoro-4-methyl-1,4-diazoniabicyclo [2.2.2]octane bis(tetrafluoroborate). NFSI = *N*-fluorobenzenesulfonamide. NFTP = 1-fluoropyridinium triflate. NIS = *N*-iodosuccinimide. DMC =dimethyl carbonate. DMF = *N*,*N*-dimethylformamide.

## Data Availability

Data are contained within the article and Supplementary Materials.

## Supplementary Materials

The following supporting information can be downloaded at: https://www.mdpi.com/article/10.3390/molecules29163899/s1, Figure S1: benzo[*d*]isothiazol-3(2*H*)-ones; Figure S2: isothiazol-3-ones, isothiazoles, and benzothiazin-4-ones; Figures S3–S67: ^1^H, ^19^F, and ^13^C NMR spectra.
