# Supplementary material for: Selective Oxidation of Benzo[d]isothiazol-3(2H)-Ones Enabled by Selectfluor
Source: Molecules. 2024 Aug 17;29(16):3899. doi: 10.3390/molecules29163899 (PMC11357611; doi:10.3390/molecules29163899)
Supplement: Supplementary file 1 [file molecules-29-03899-s001.zip › molecules-3146555-supplementary.pdf]

Supplementary Materials

Selective oxidation of benzo[*d*]isothiazol-3(2*H*)-ones enabled by Selectfluor

Qin Li [1], Dan Yuan [1], Chong Liu [2], Faith Herington [2], Ke Yang [1,*] and Haibo Ge [2,*]

[1] Jiangsu Key Laboratory of Advanced Catalytic Materials & Technology, School of Petrochemical Engineering, Changzhou University, Changzhou, Jiangsu 213164, China
[2] Department of Chemistry and Biochemistry, Texas Tech University, Lubbock, Texas 79409, USA
* Correspondence: keyang@cczu.edu.cn (K.Y.); haibo.ge@ttu.edu (H.G.)

Table of contents

SI. Starting materials································S2

SII. ^{1}H, ^{19}F and ^{13}C NMR Spectra································S3-S35

SI. Starting materials

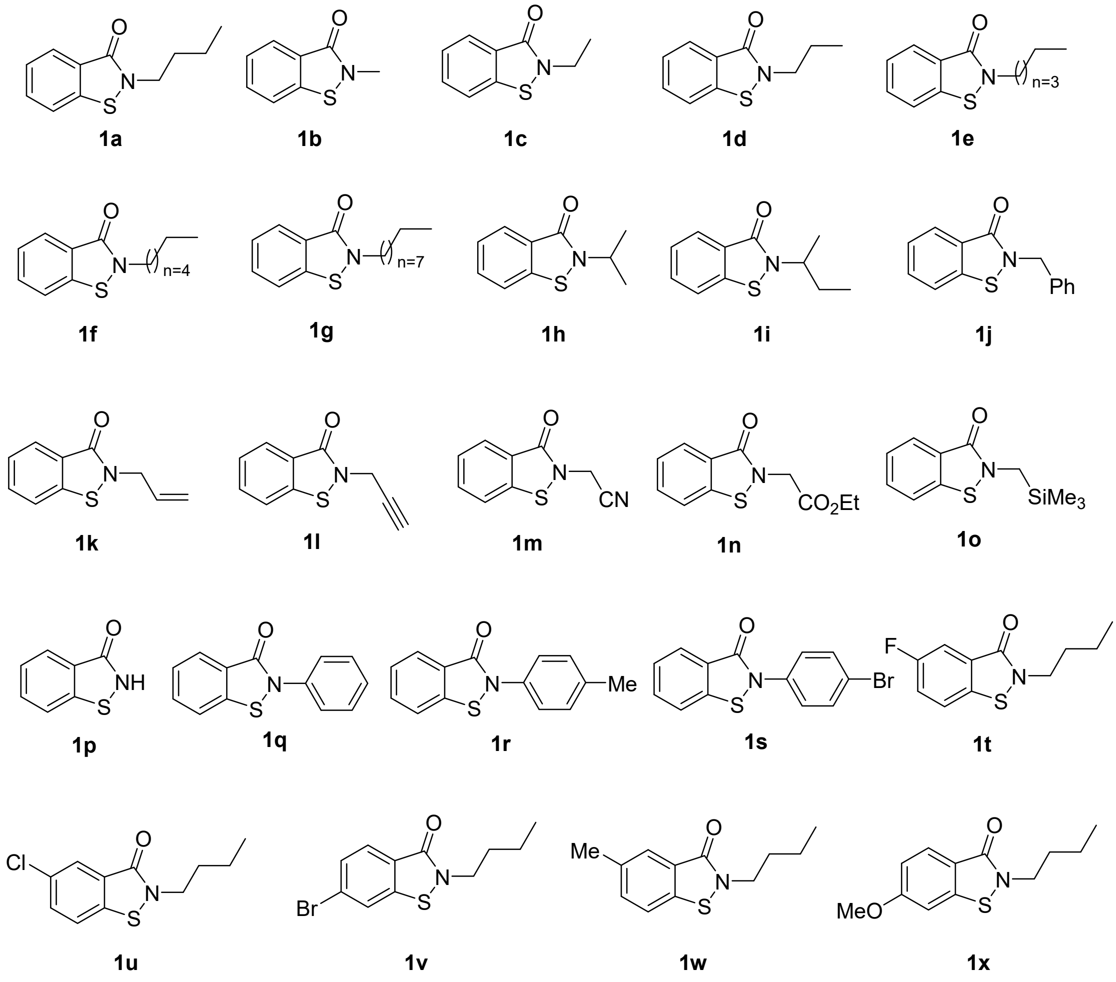

Figure S1 benzo[*d*]isothiazol-3(2*H*)-ones

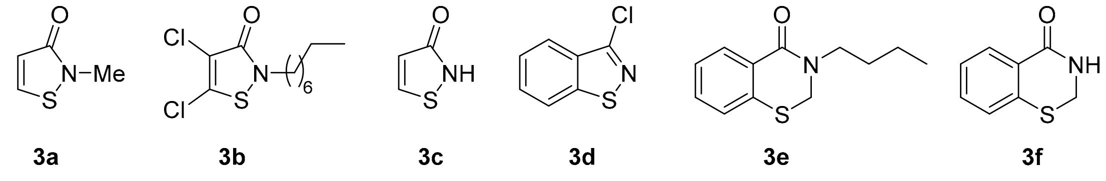

Figure S2. isothiazol-3-ones, isothiazoles and benzothiazin-4-ones

SII. ^{1}H, ^{19}F and ^{13}C NMR Spectra

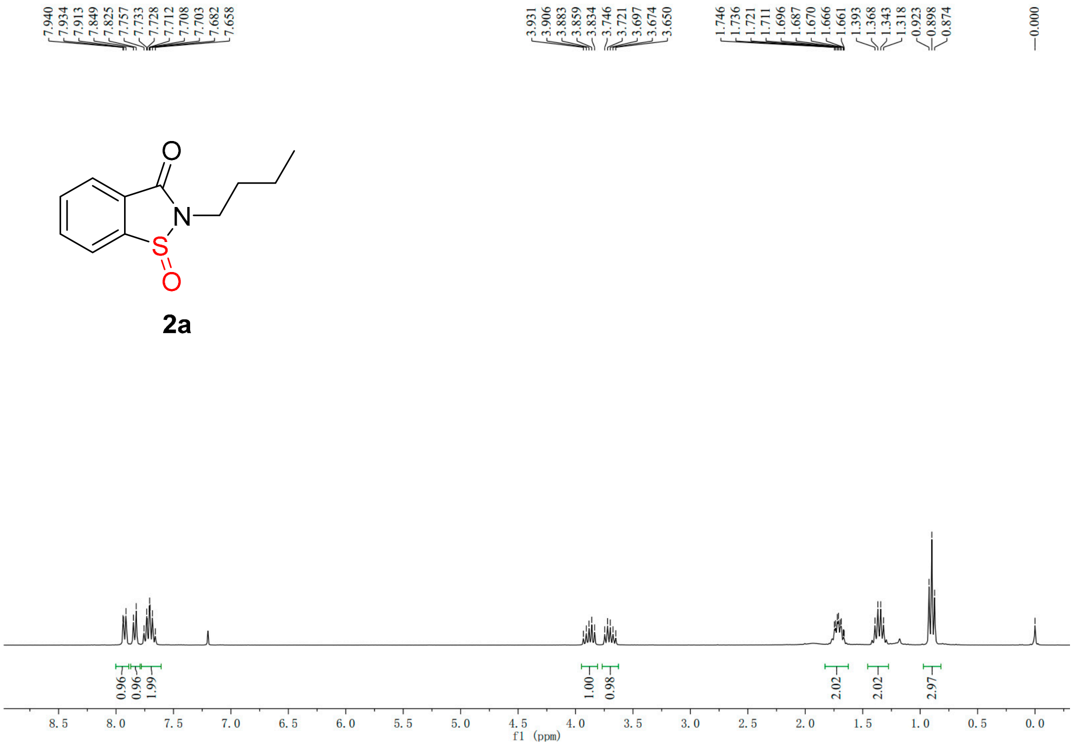

Figure S3 ^{1}H NMR spectrum (300MHz, $CDCl_3$, 298K) of **2a**

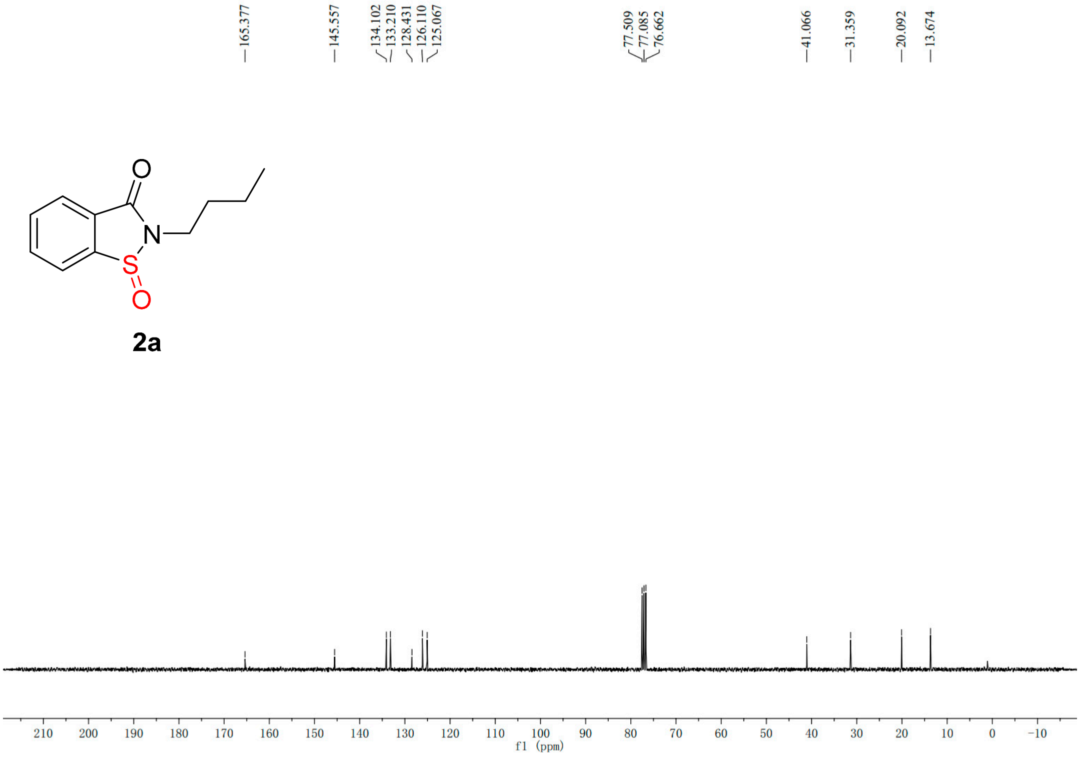

Figure S4 ^{13}C NMR spectrum (75MHz, $CDCl_3$, 298K) of **2a**

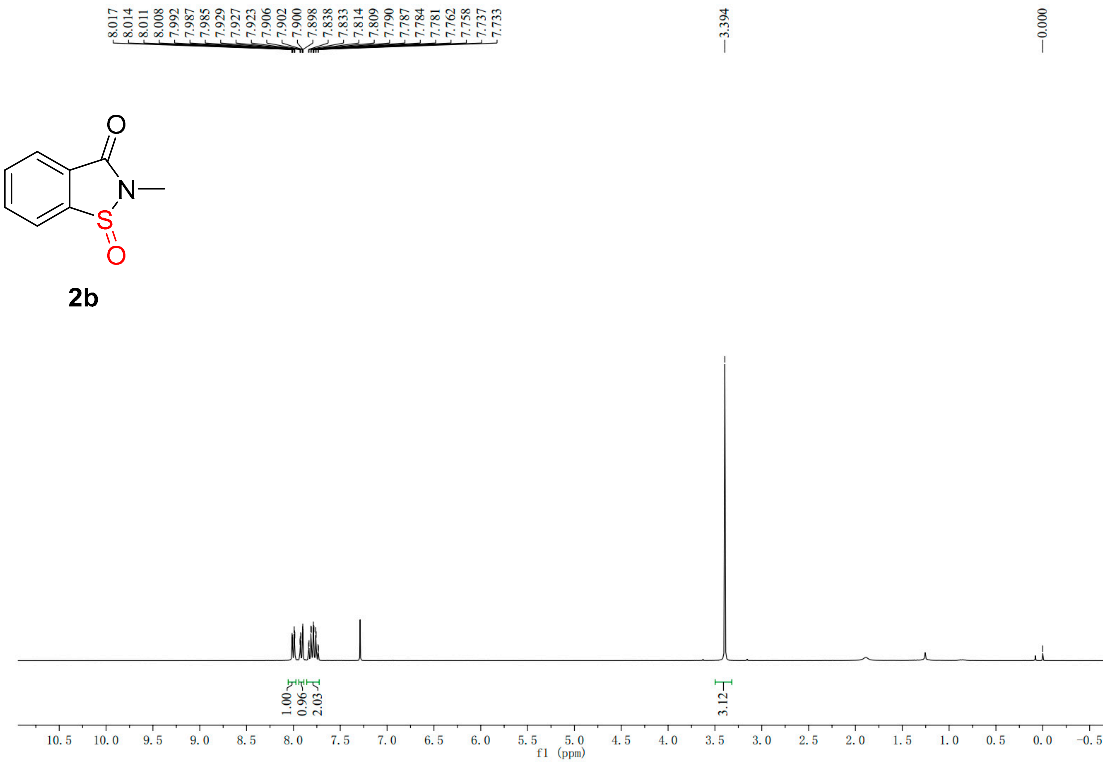

Figure S5 ^{1}H NMR spectrum (300MHz, $CDCl_3$, 298K) of **2b**

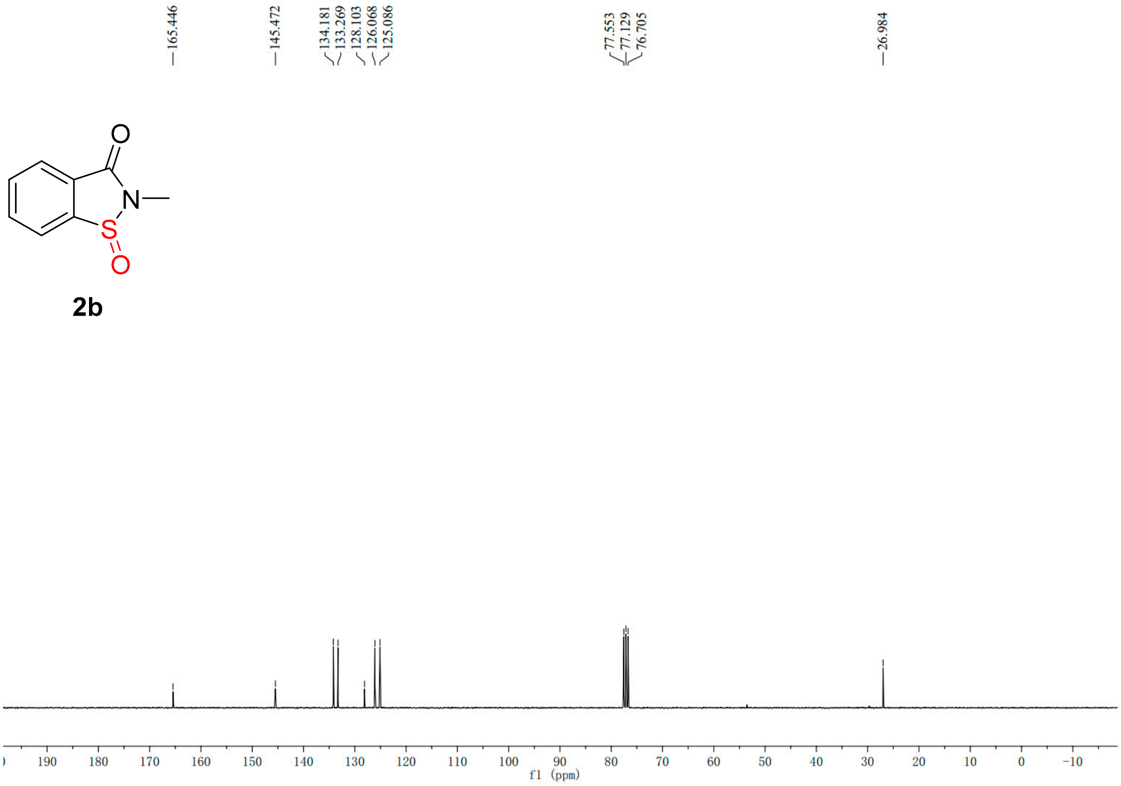

Figure S6 ^{13}C NMR spectrum (75MHz, $CDCl_3$, 298K) of **2b**

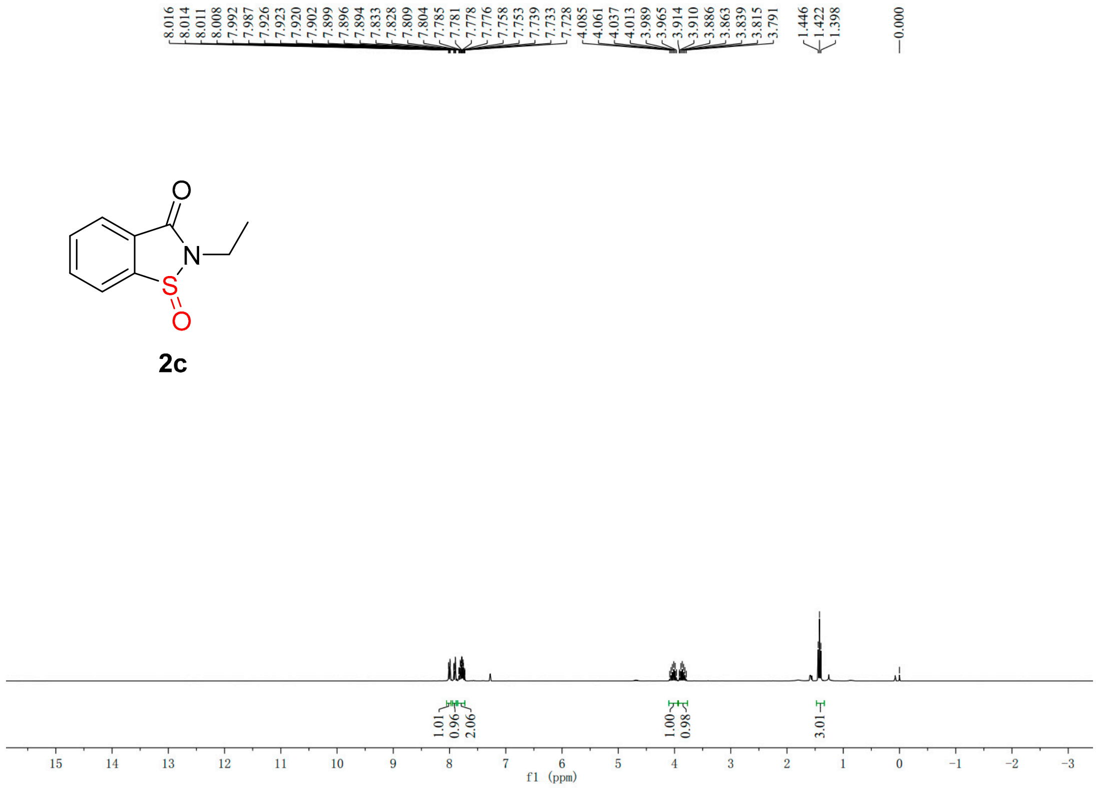

Figure S7 ^{1}H NMR spectrum (300MHz, $CDCl_3$, 298K) of **2c**

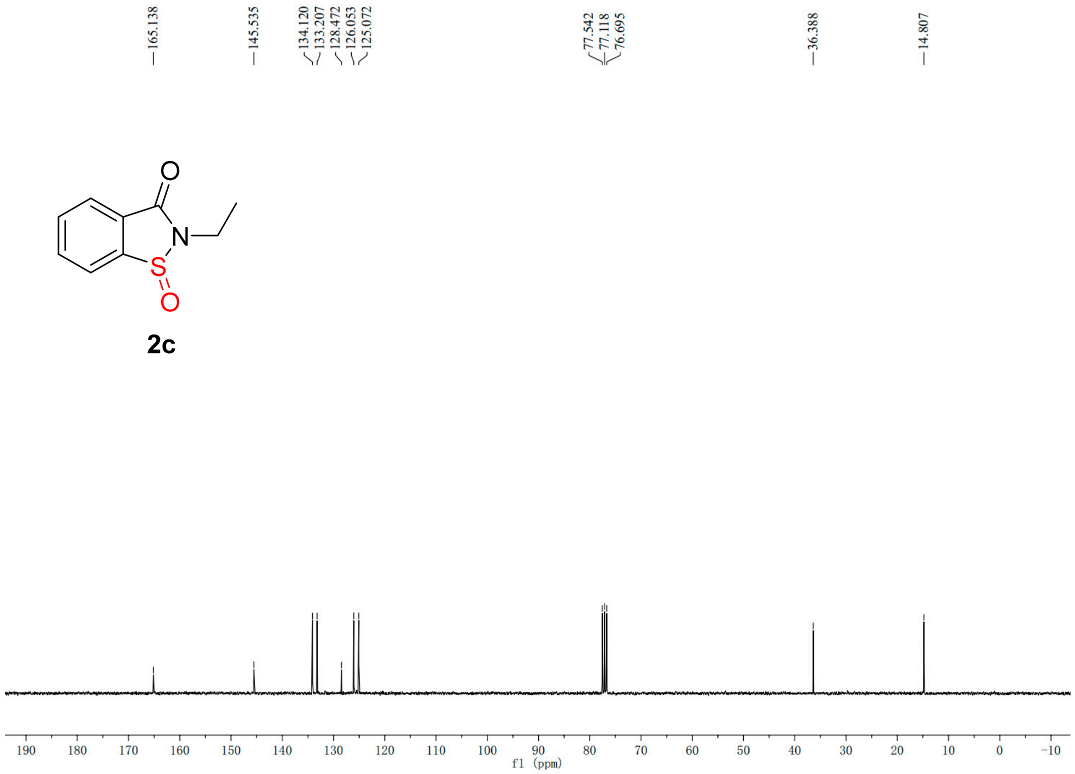

Figure S8 ^{13}C NMR spectrum (75MHz, $CDCl_3$, 298K) of **2c**

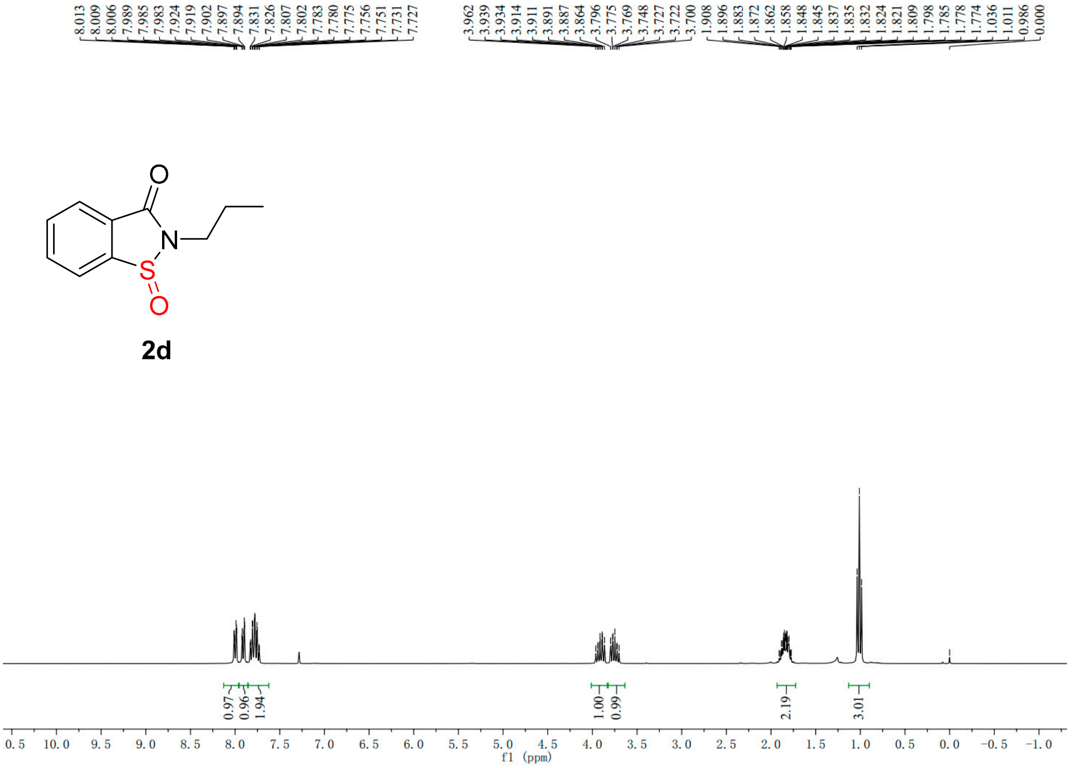

Figure S9 ^{1}H NMR spectrum (300MHz, $CDCl_3$, 298K) of **2d**

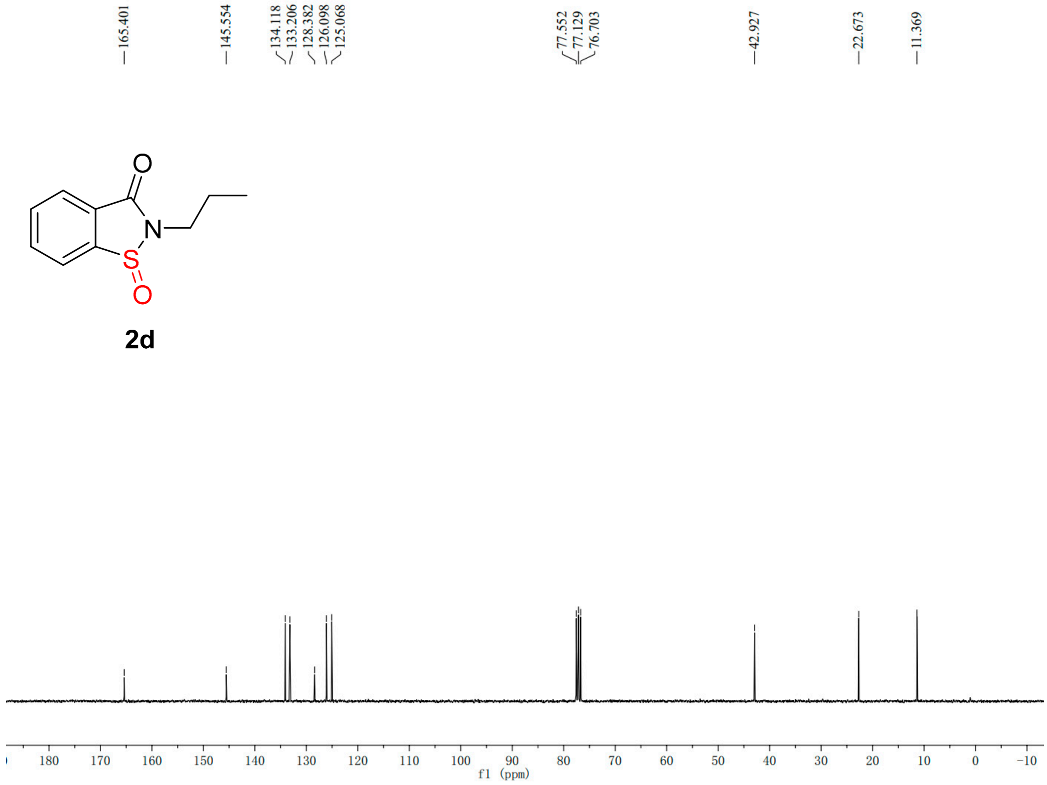

Figure S10 ^{13}C NMR spectrum (75MHz, $CDCl_3$, 298K) of **2d**

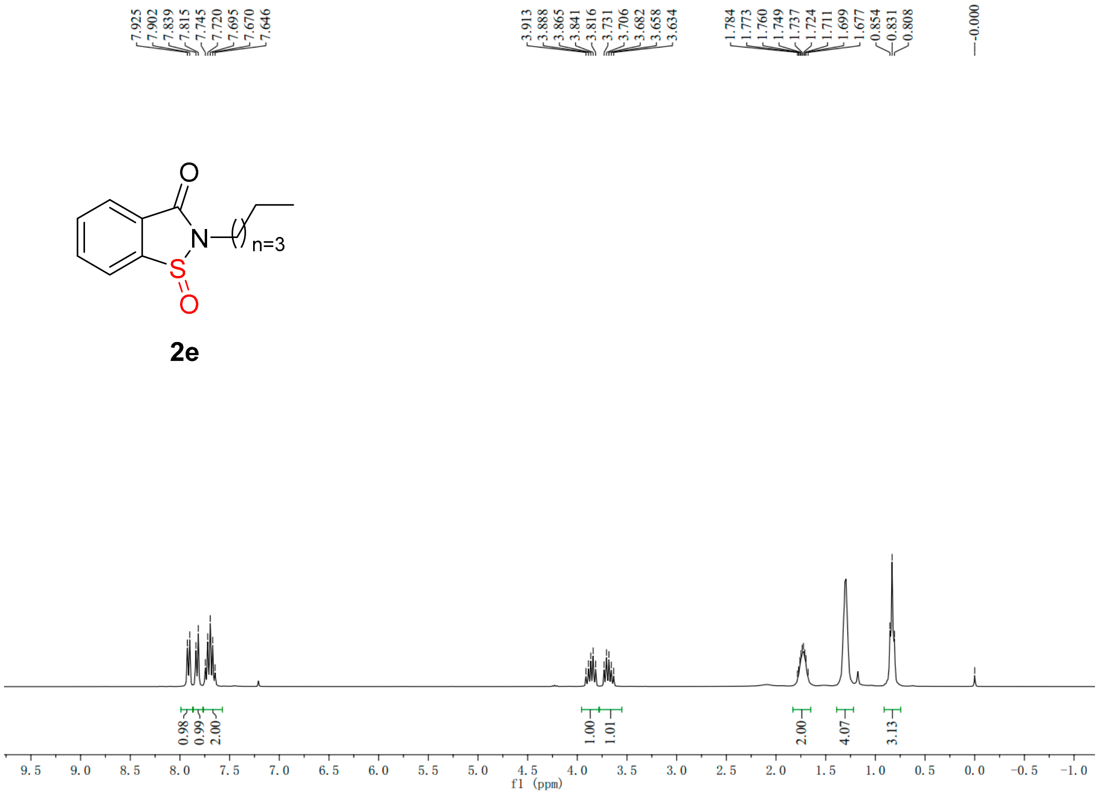

Figure S11 ^{1}H NMR spectrum (300MHz, $CDCl_3$, 298K) of **2e**

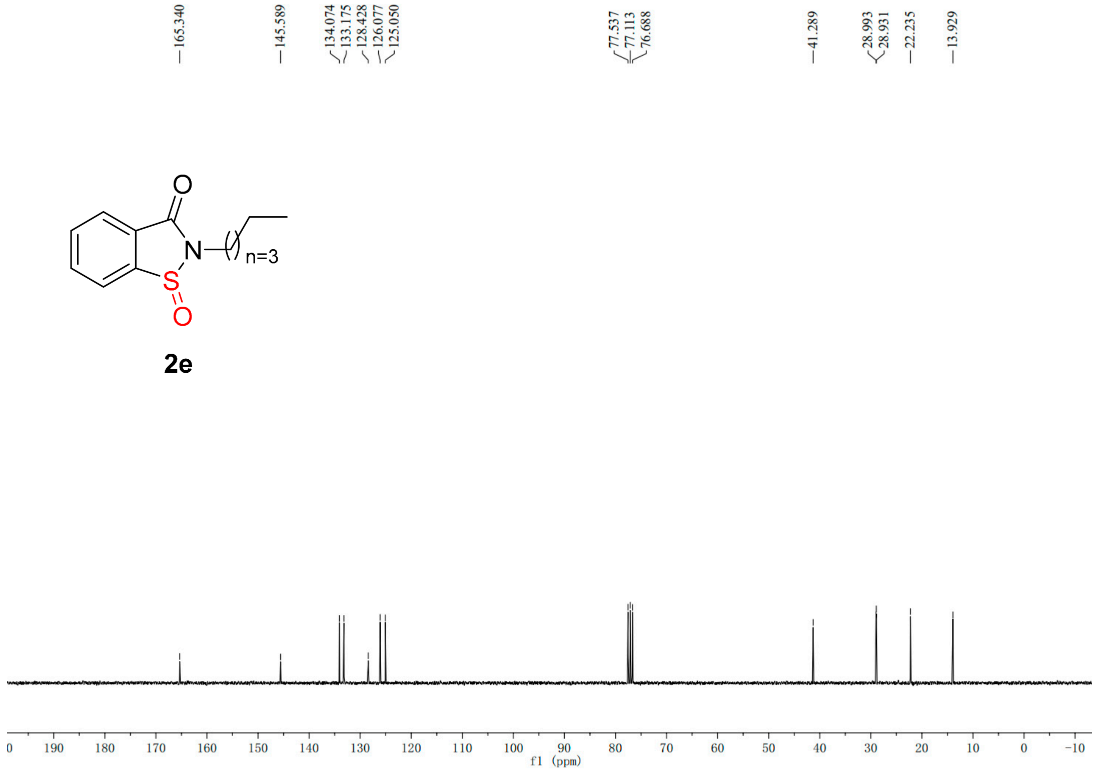

Figure S12 ^{13}C NMR spectrum (75MHz, $CDCl_3$, 298K) of **2e**

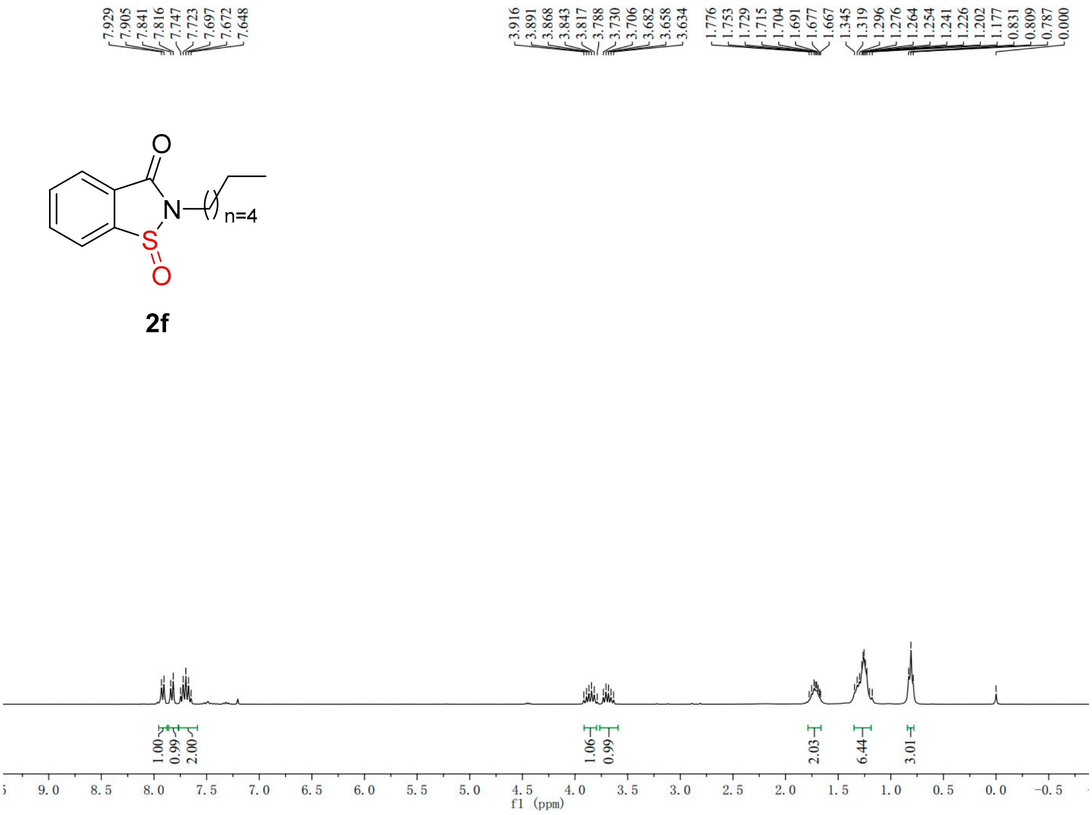

Figure S13 ^{1}H NMR spectrum (300MHz, CDCl3, 298K) of **2f**

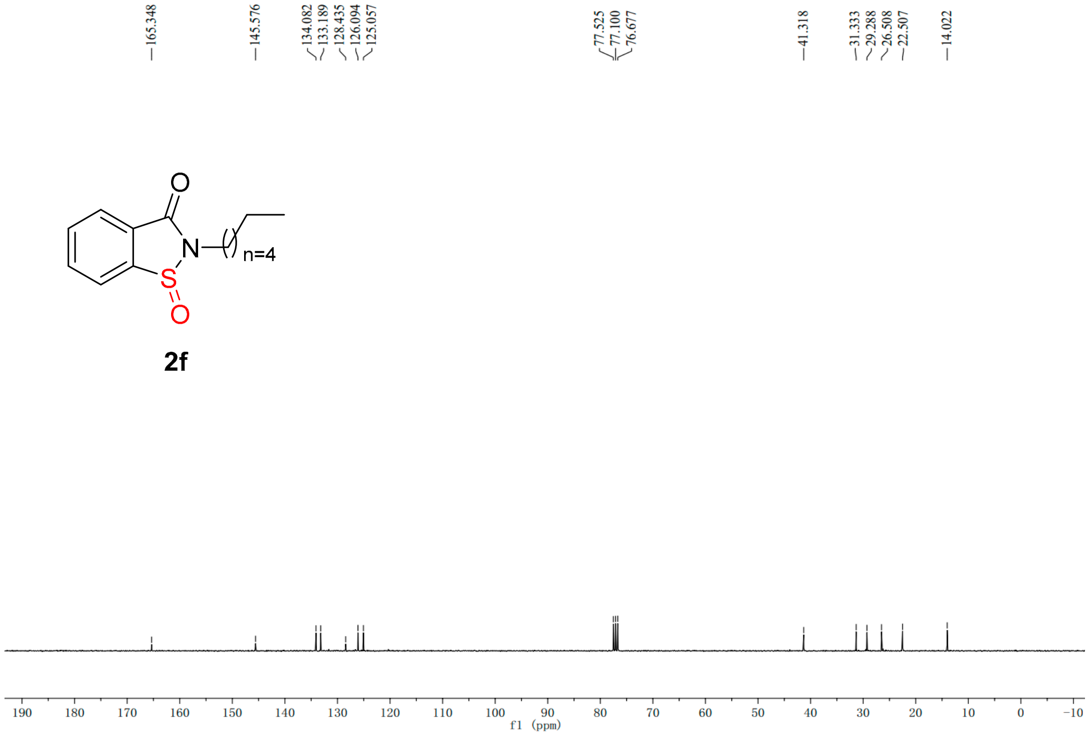

Figure S14 ^{13}C NMR spectrum (75MHz, CDCl3, 298K) of **2f**

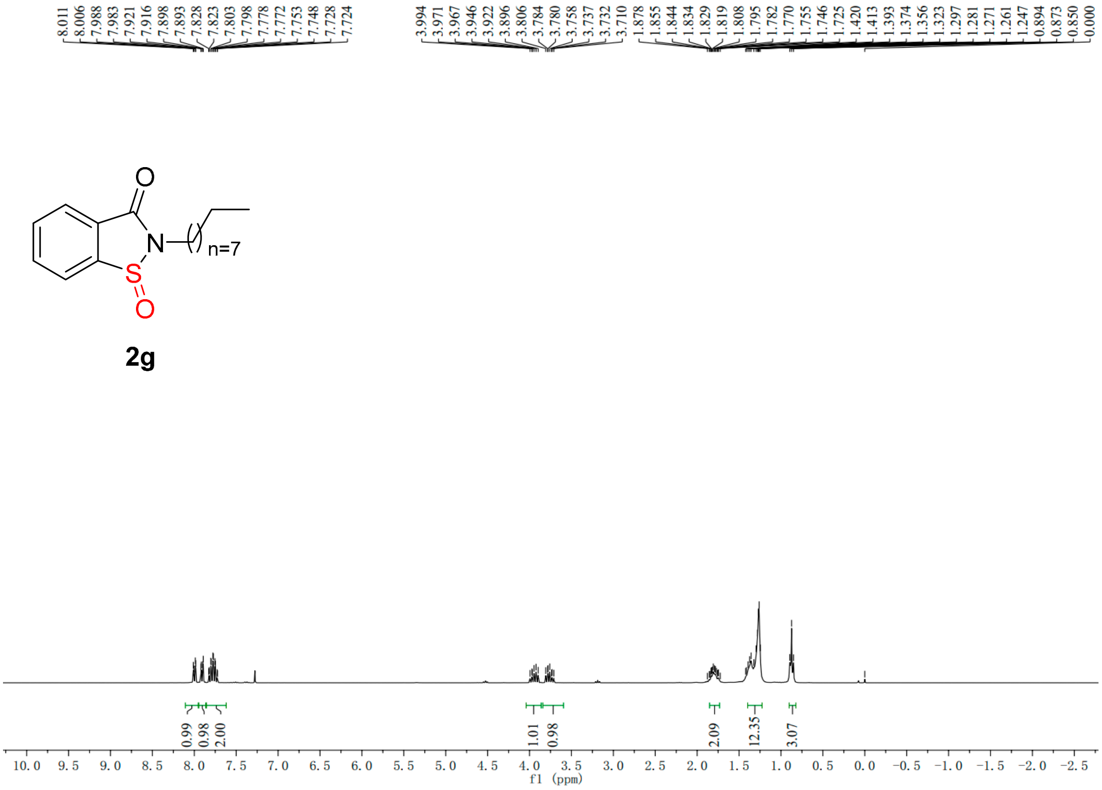

Figure S15 ¹H NMR spectrum (300MHz, $CDCl_3$, 298K) of **2g**

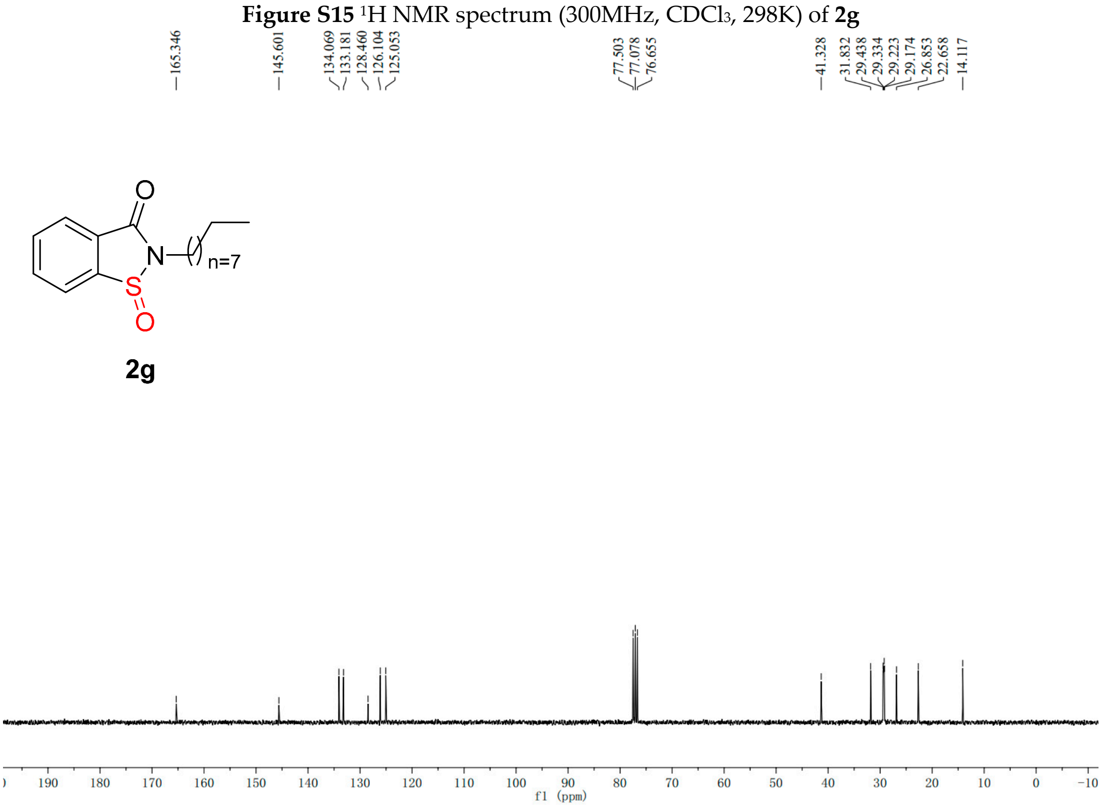

Figure S16 ¹³C NMR spectrum (75MHz, $CDCl_3$, 298K) of **2g**

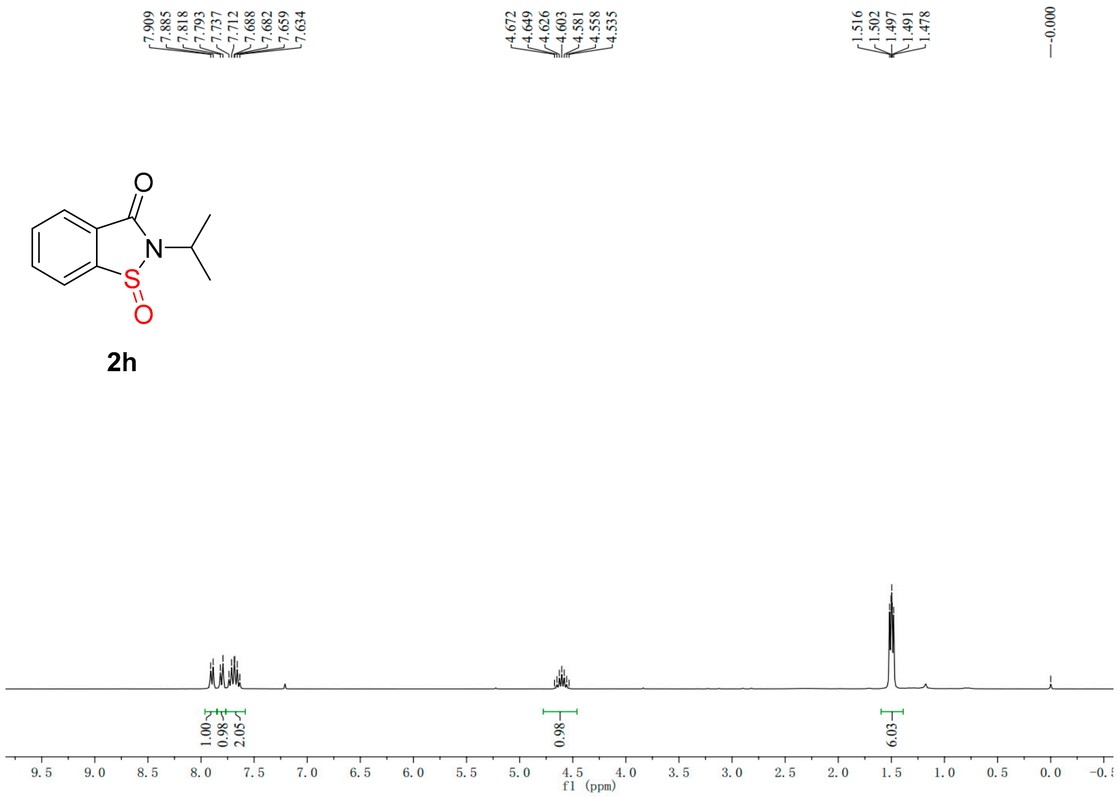

Figure S17 ¹H NMR spectrum (300MHz, $CDCl_3$, 298K) of **2h**

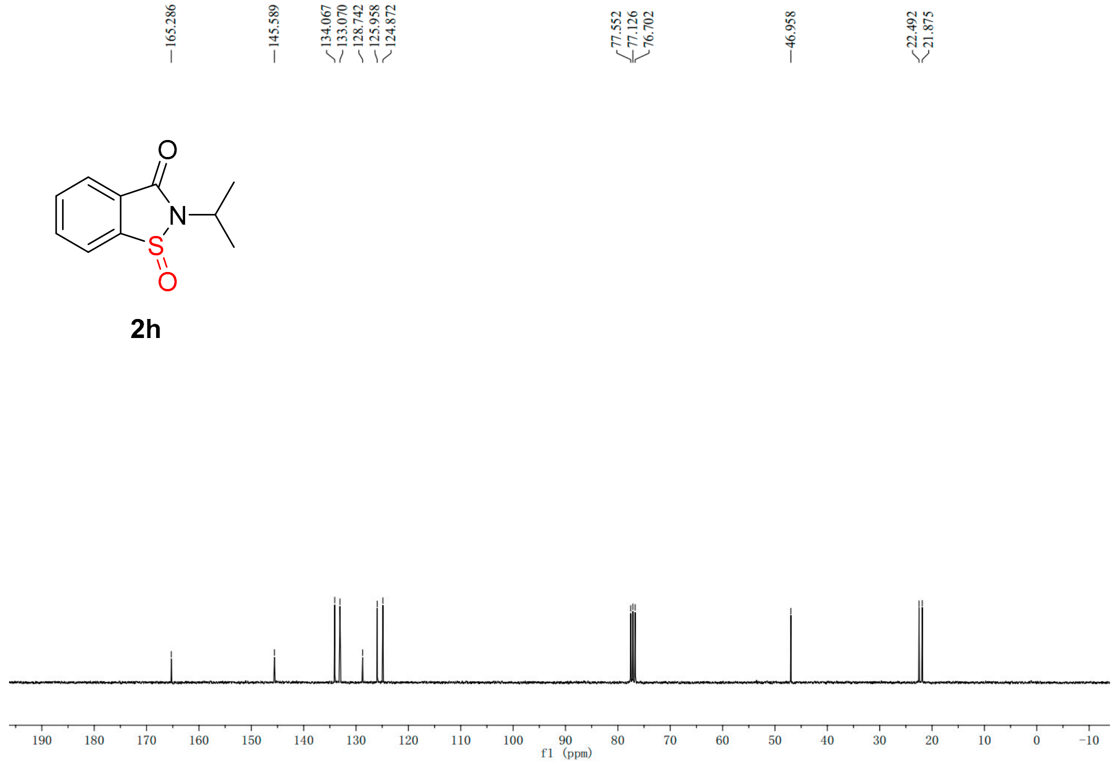

Figure S18 ¹³C NMR spectrum (75MHz, $CDCl_3$, 298K) of **2h**

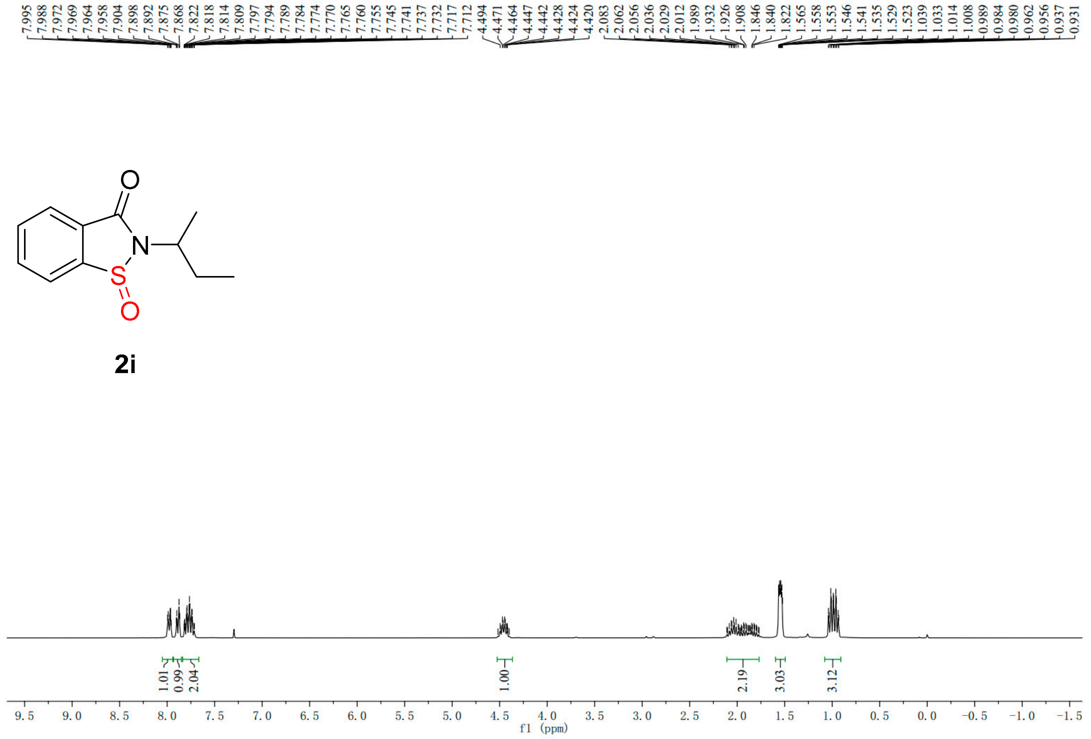

Figure S19 ^{1}H NMR spectrum (300MHz, $CDCl_3$, 298K) of **2i**

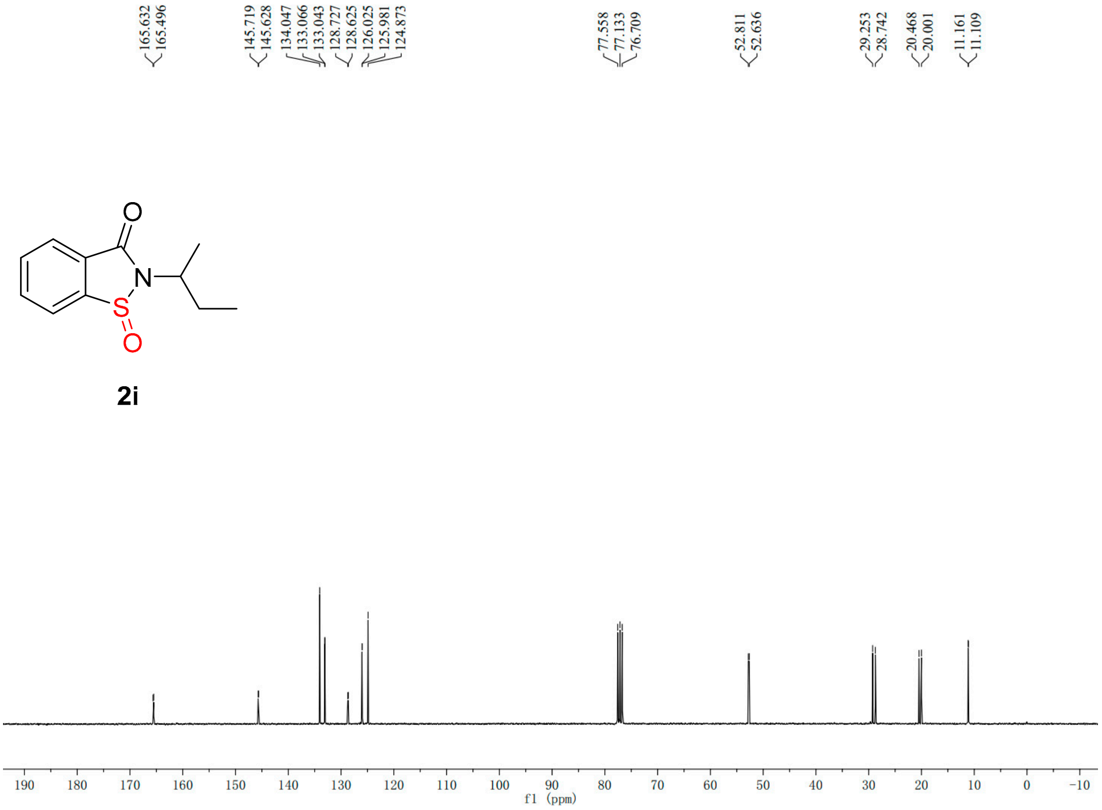

Figure S20 ^{13}C NMR spectrum (75MHz, $CDCl_3$, 298K) of **2i**

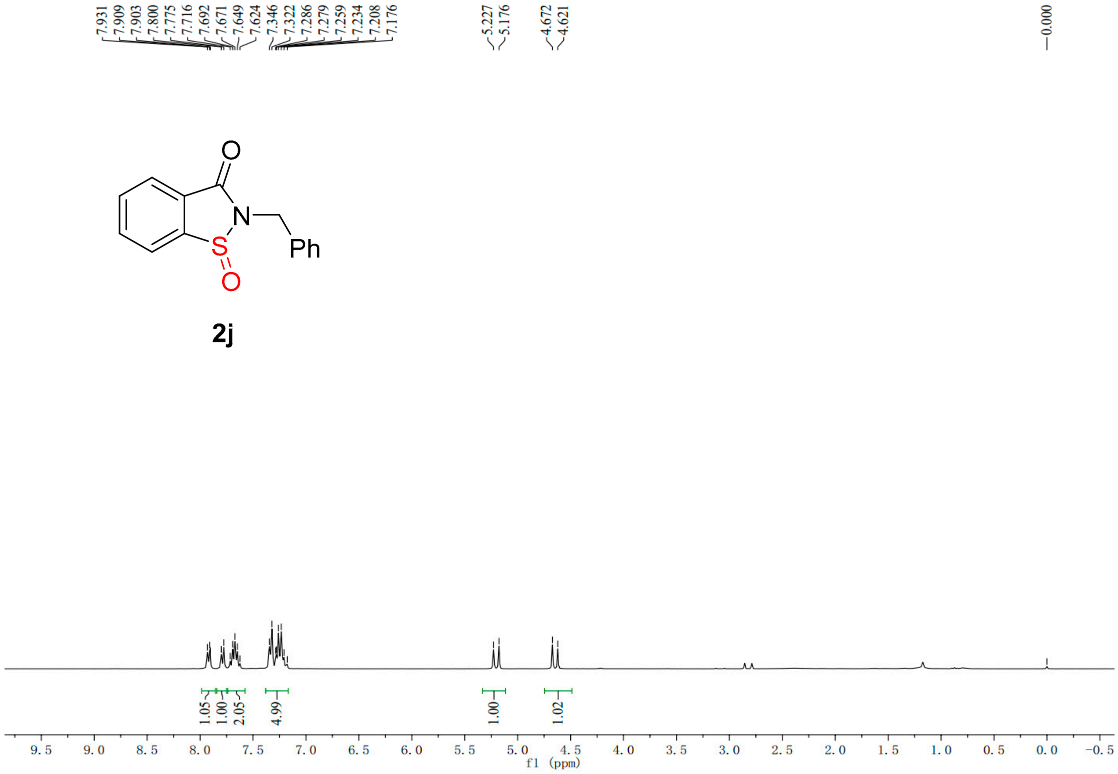

Figure S21 ^{1}H NMR spectrum (300MHz, $CDCl_3$, 298K) of **2j**

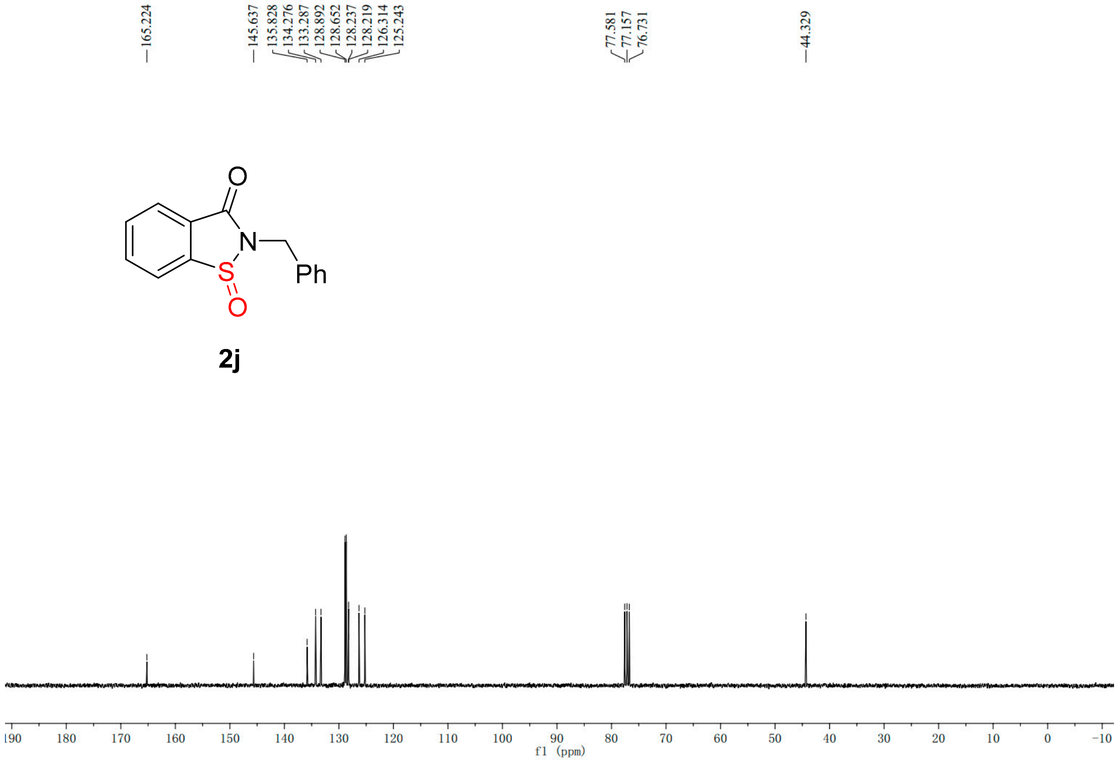

Figure S22 ^{13}C NMR spectrum (75MHz, $CDCl_3$, 298K) of **2j**

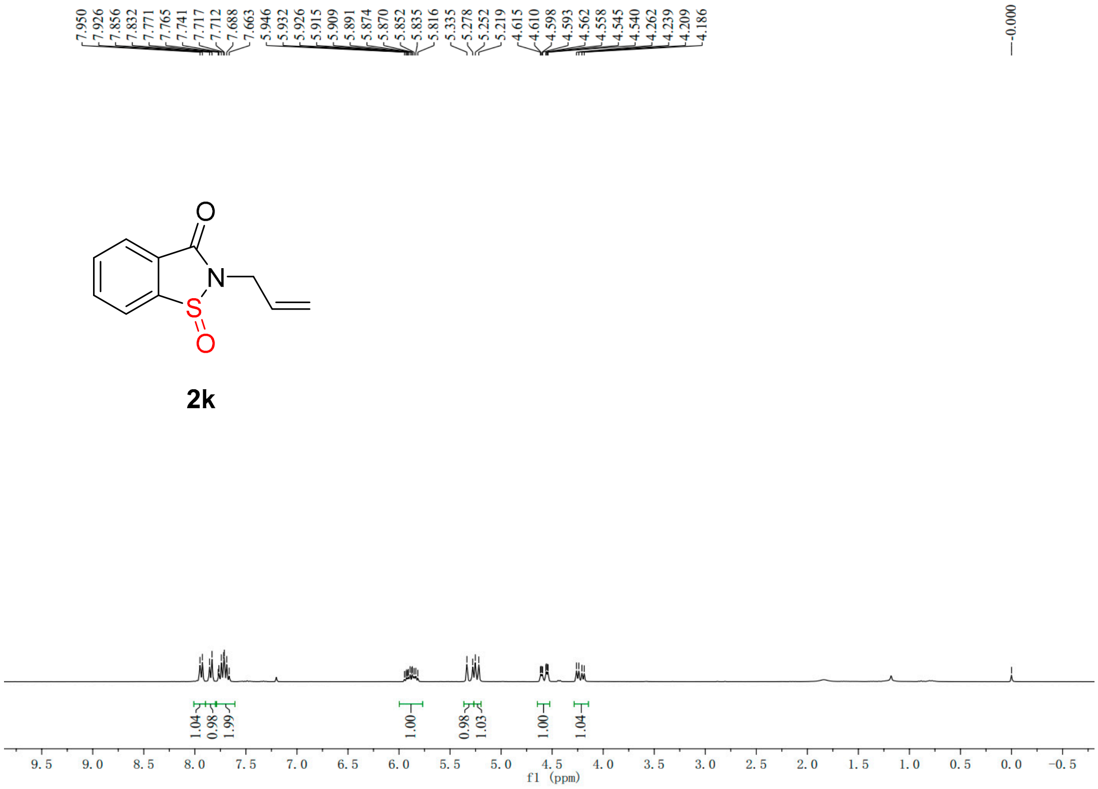

Figure S23 ^{1}H NMR spectrum (300MHz, $CDCl_3$, 298K) of **2k**

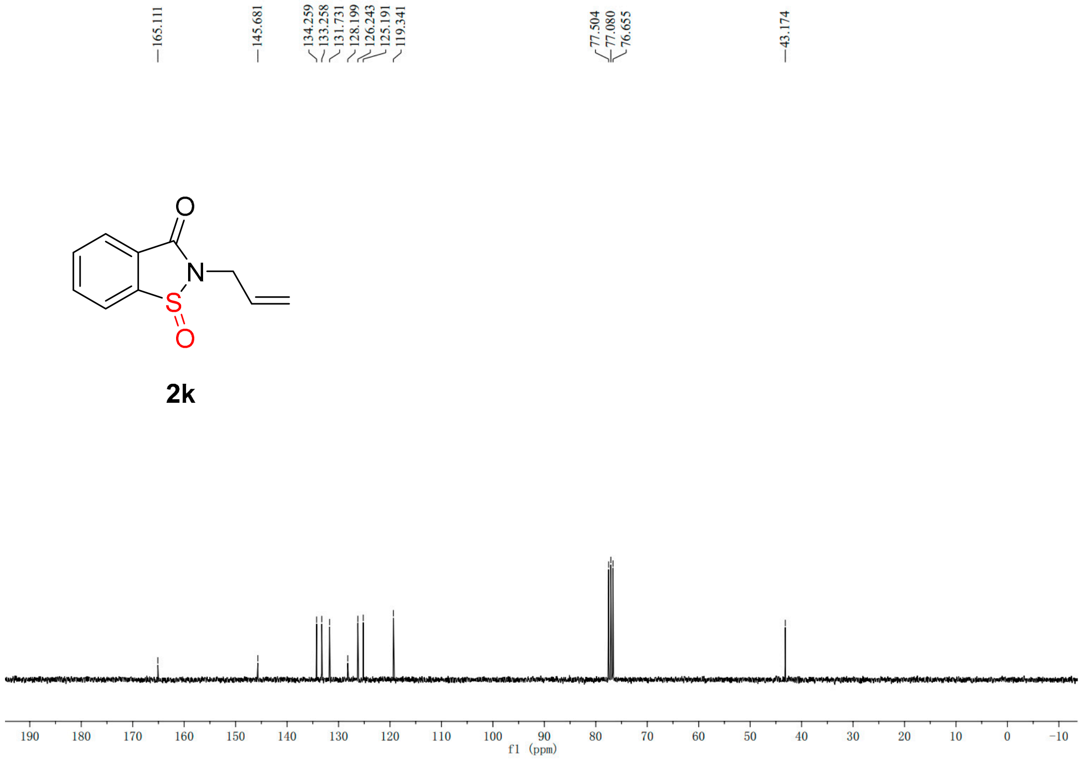

Figure S24 ^{13}C NMR spectrum (75MHz, $CDCl_3$, 298K) of **2k**

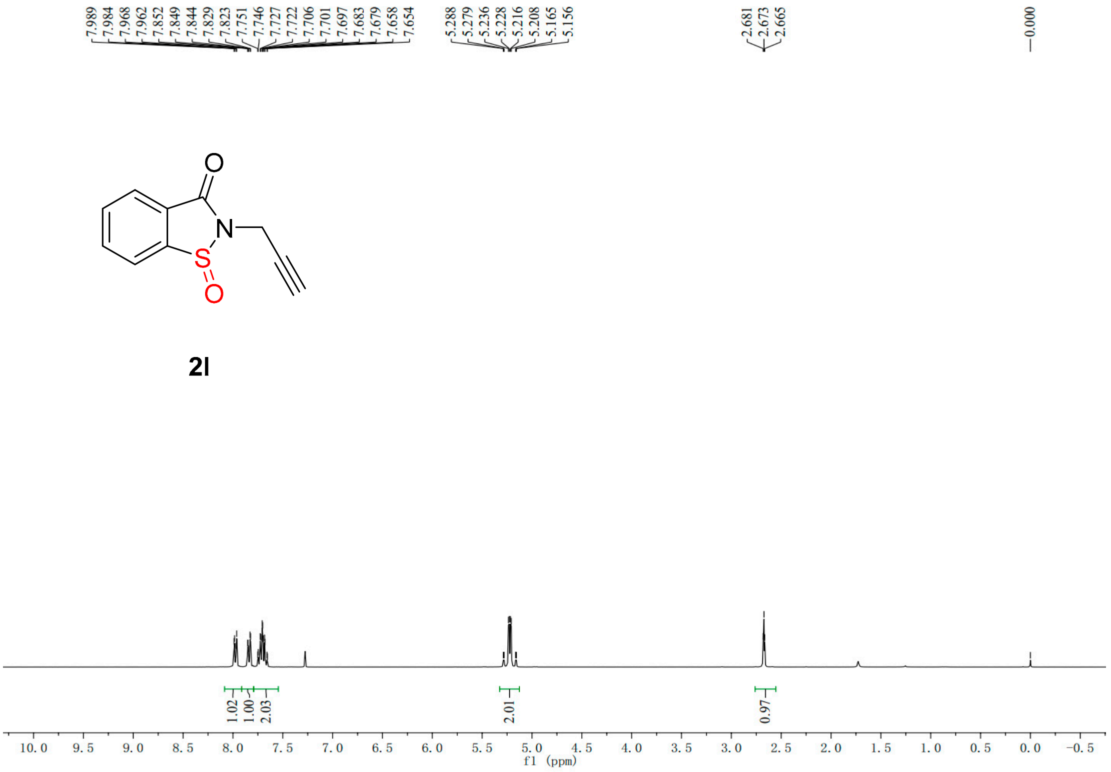

Figure S25 ^{1}H NMR spectrum (300MHz, $CDCl_3$, 298K) of **2l**

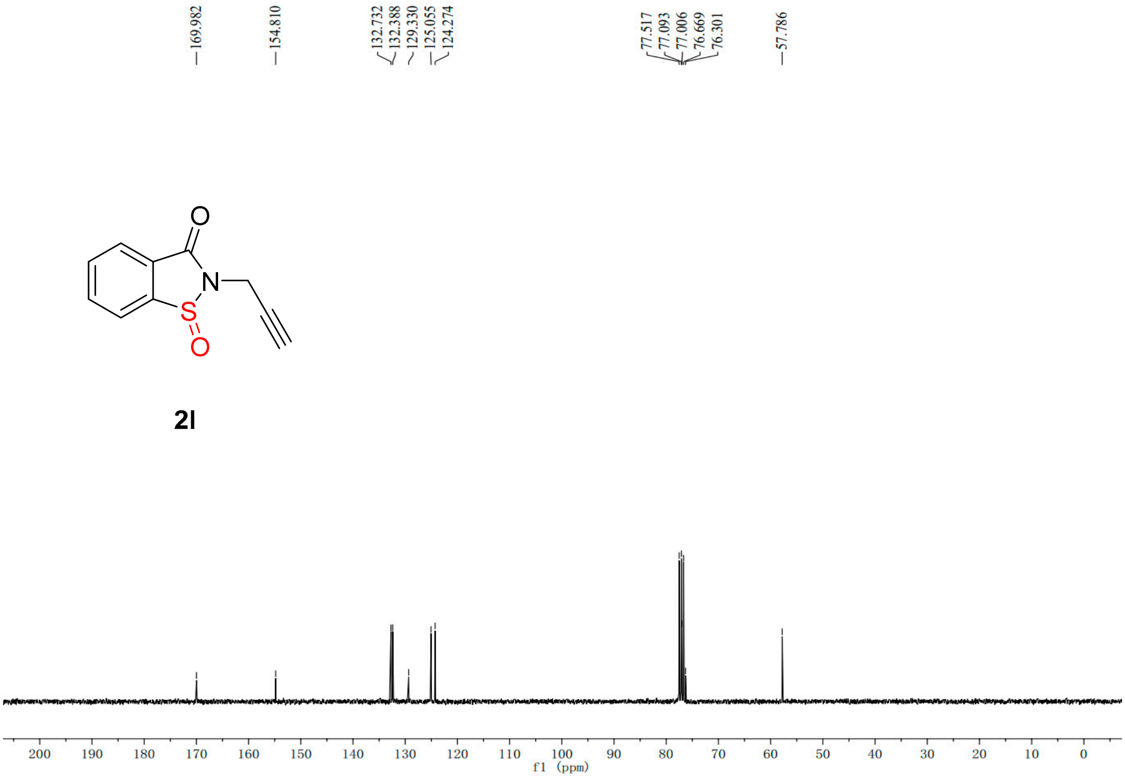

Figure S26 ^{13}C NMR spectrum (75MHz, $CDCl_3$, 298K) of **2l**

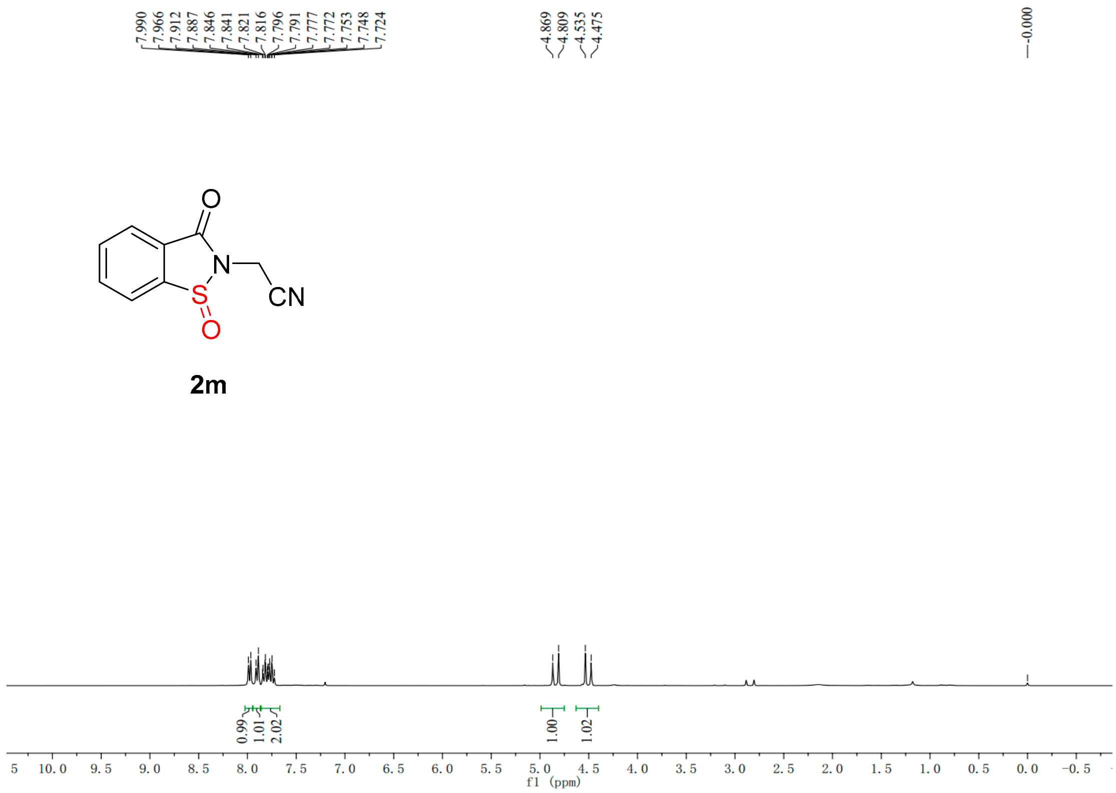

Figure S27 ^{1}H NMR spectrum (300MHz, $CDCl_3$, 298K) of **2m**

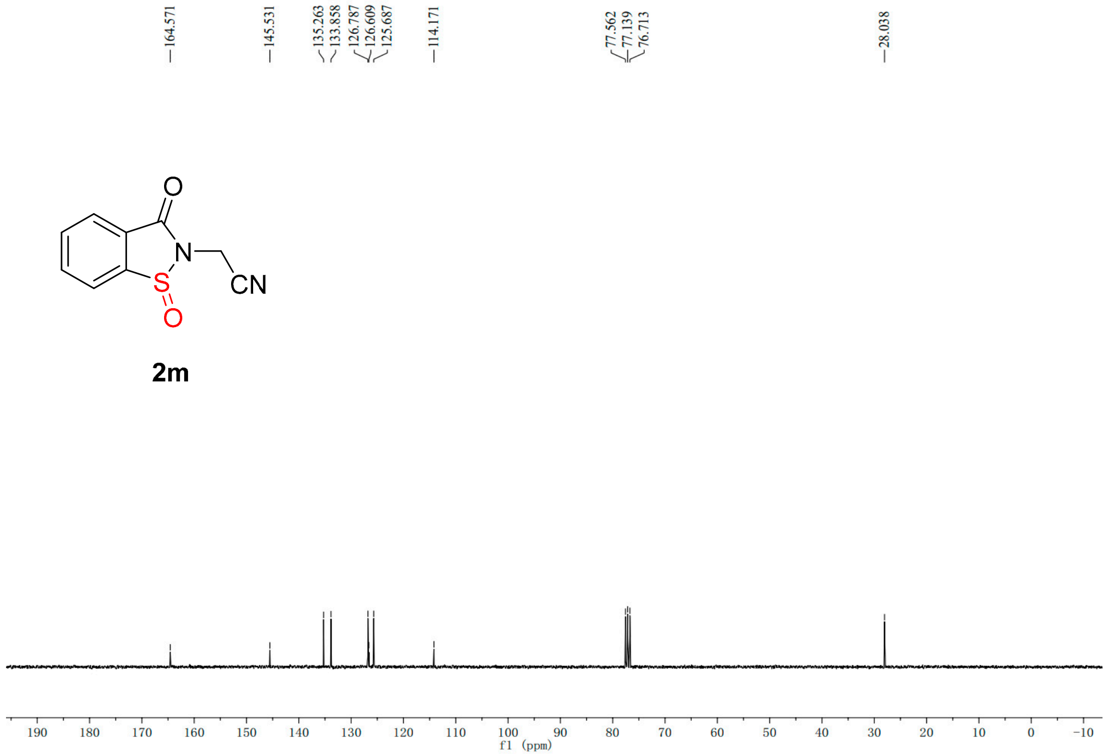

Figure S28 ^{13}C NMR spectrum (75MHz, $CDCl_3$, 298K) of **2m**

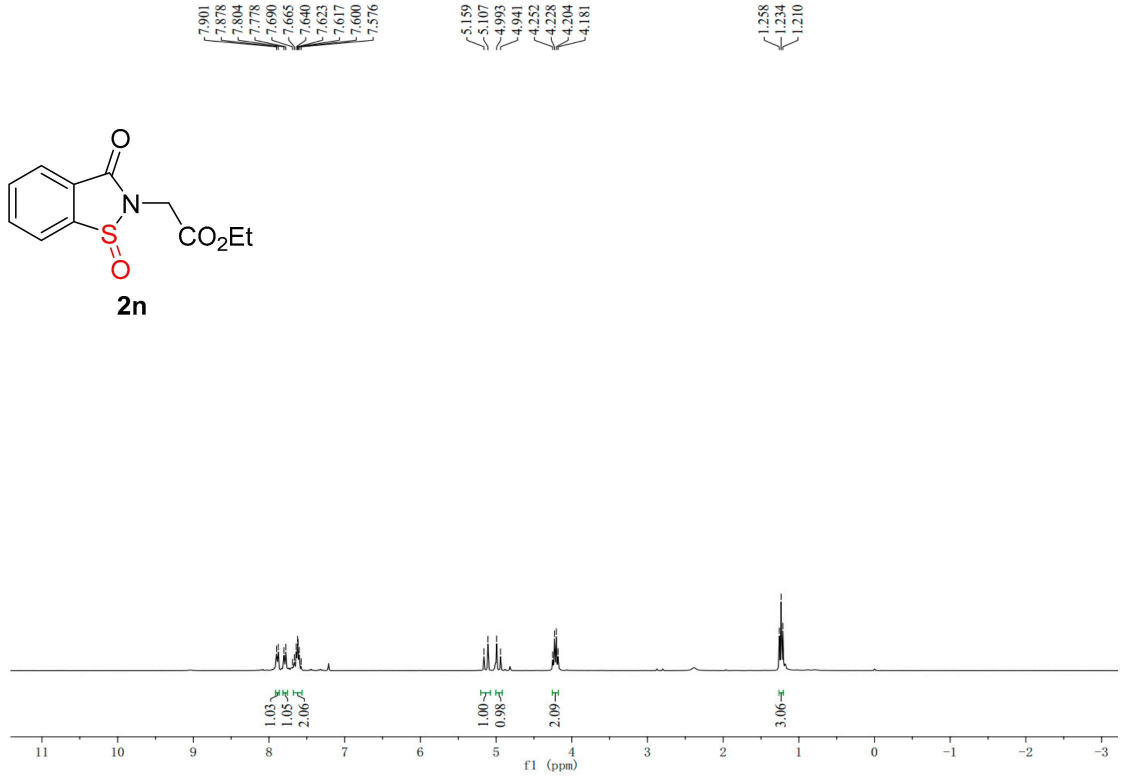

Figure S29 ^{1}H NMR spectrum (300MHz, $CDCl_3$, 298K) of **2n**

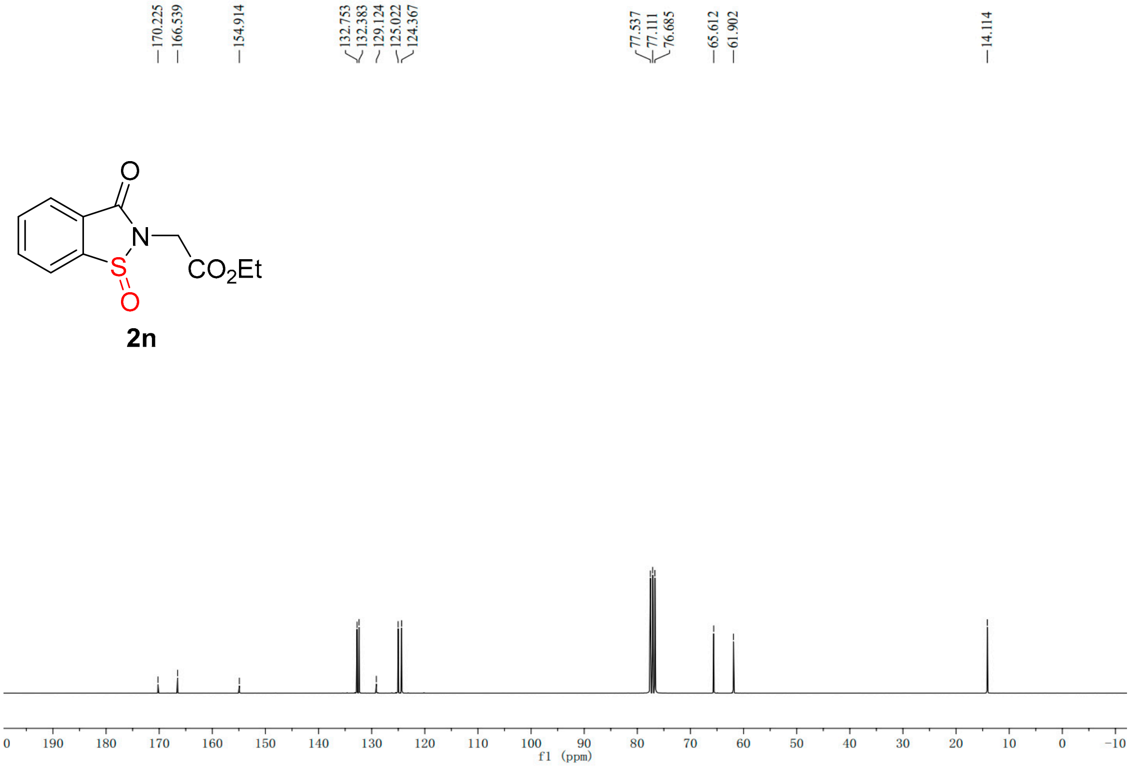

Figure S30 ^{13}C NMR spectrum (75MHz, $CDCl_3$, 298K) of **2n**

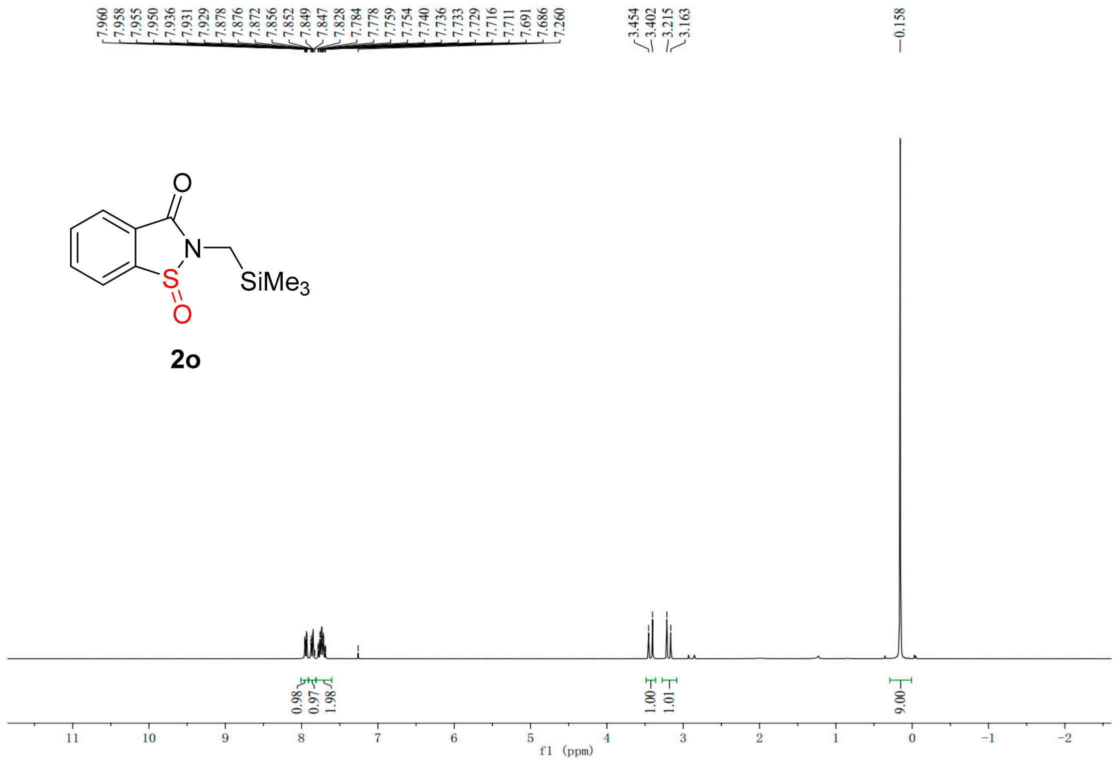

Figure S31 ^{1}H NMR spectrum (300MHz, $CDCl_3$, 298K) of **2o**

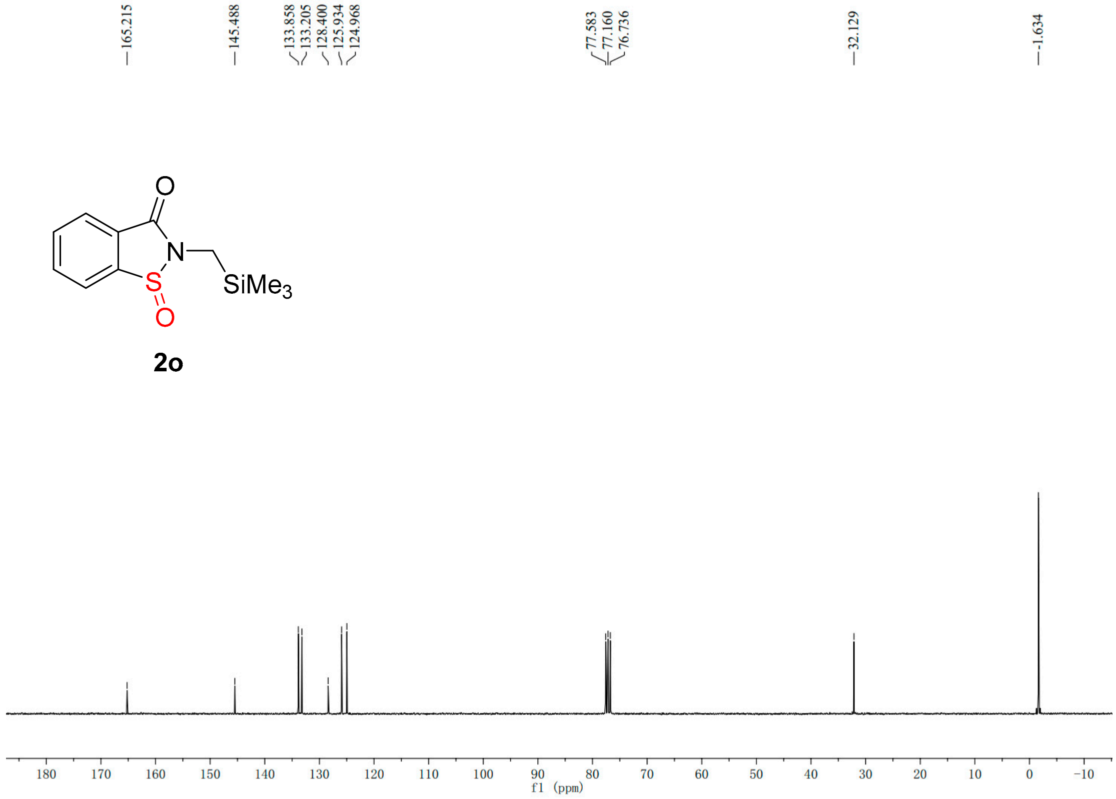

Figure S32 ^{13}C NMR spectrum (75MHz, $CDCl_3$, 298K) of **2o**

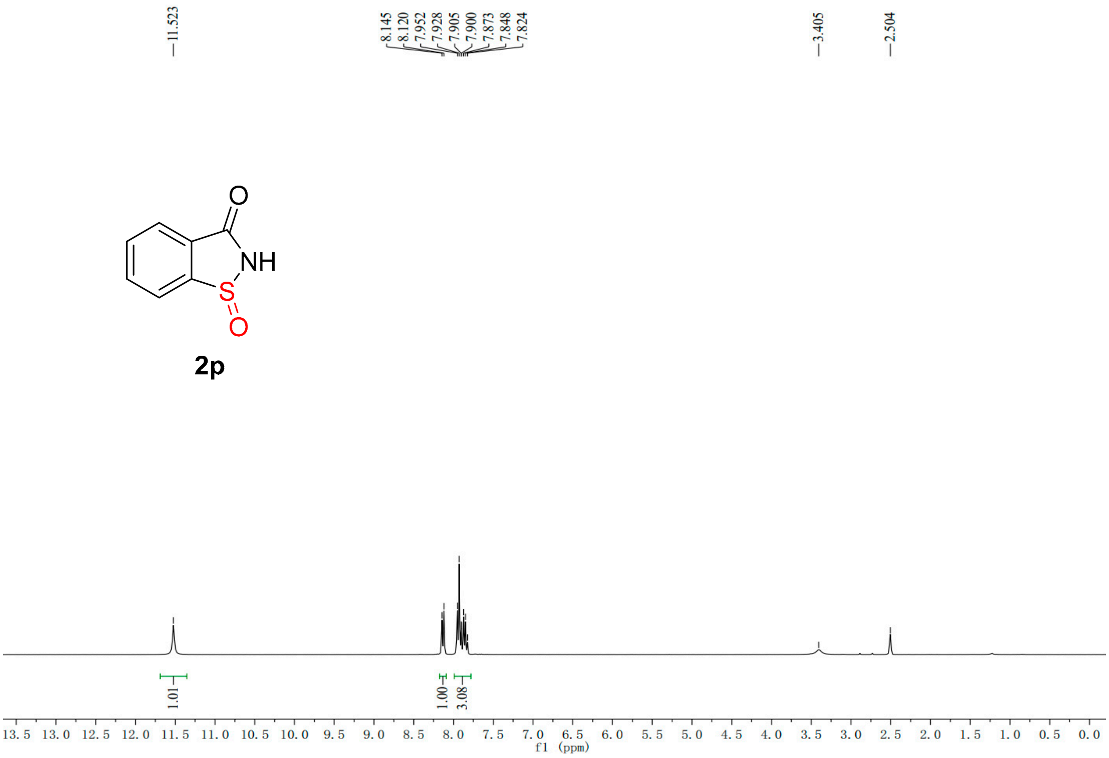

Figure S33 ^{1}H NMR spectrum (300MHz, DMSO, 298K) of **2p**

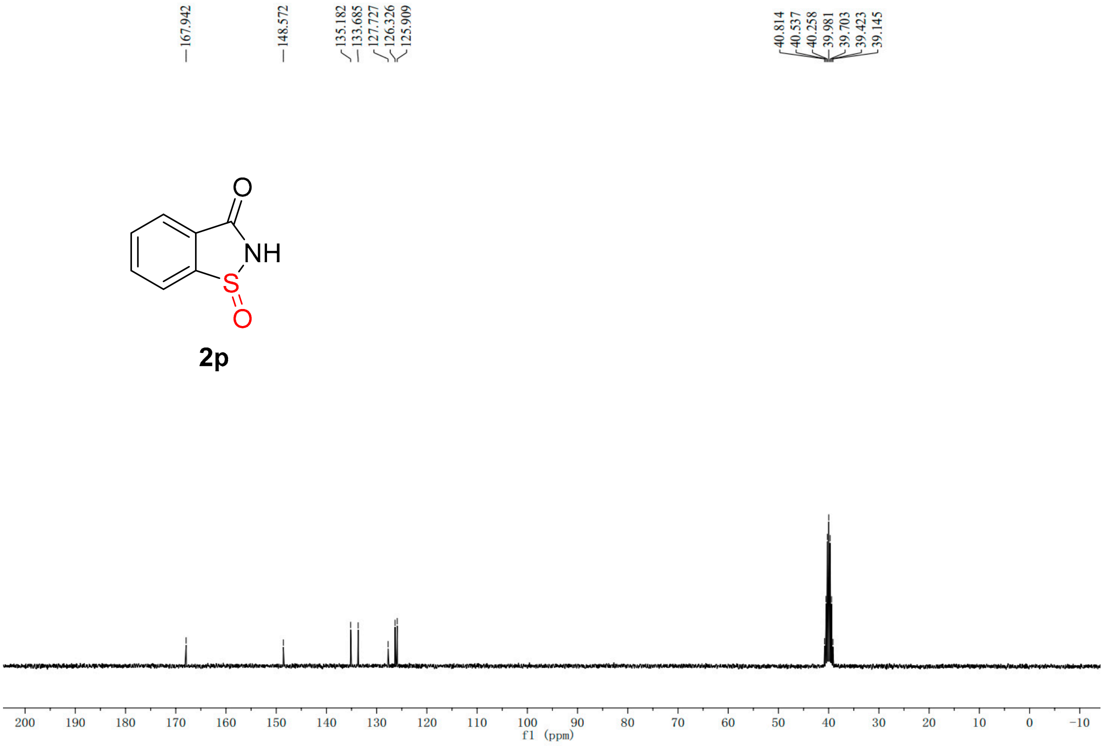

Figure S34 ^{13}C NMR spectrum (75MHz, DMSO, 298K) of **2p**

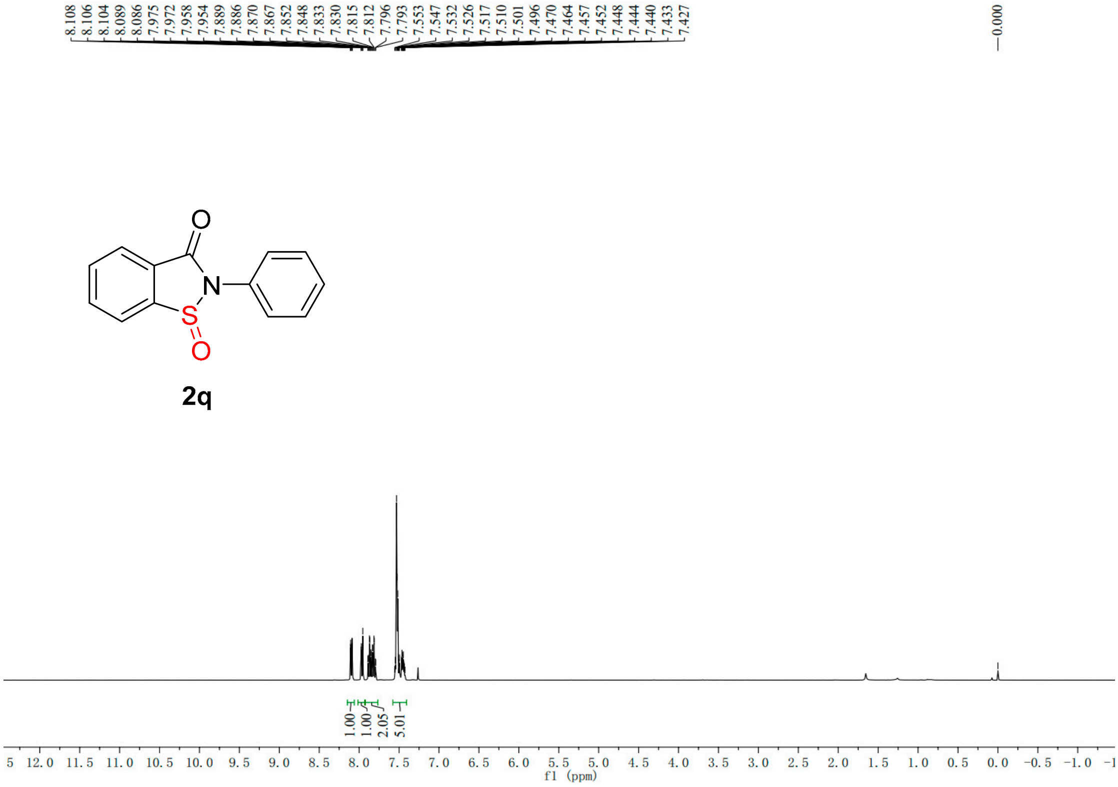

Figure S35 ¹H NMR spectrum (400MHz, CDCl₃, 298K) of **2q**

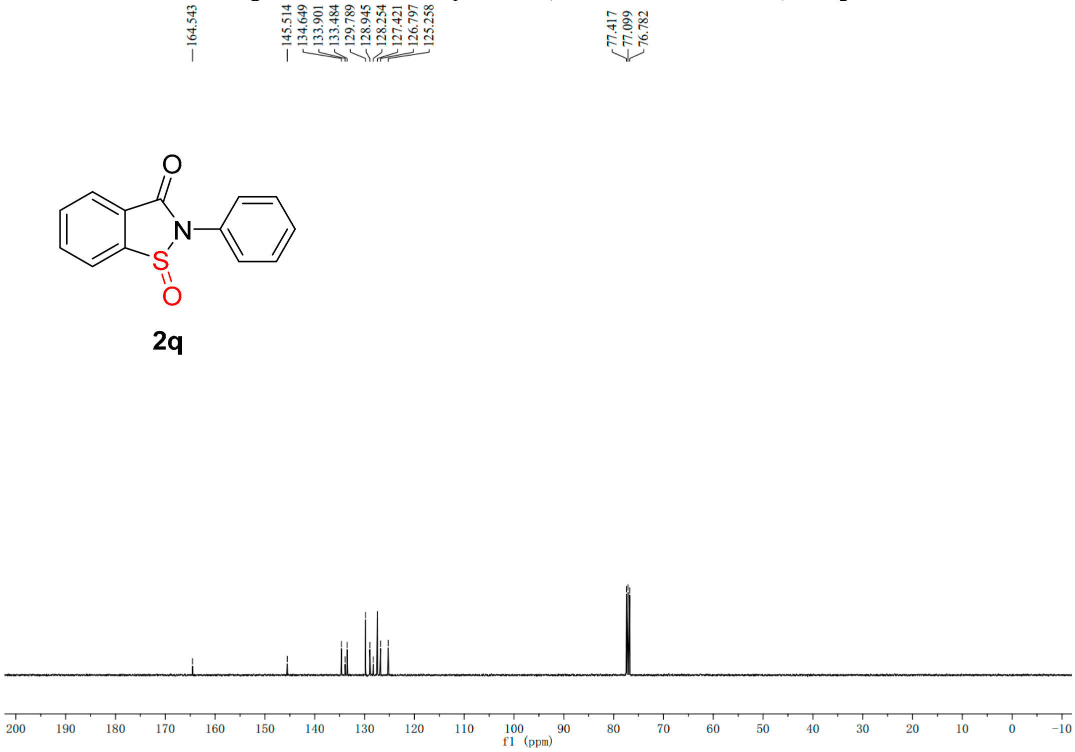

Figure S36 ¹³C NMR spectrum (101MHz, CDCl₃, 298K) of **2q**

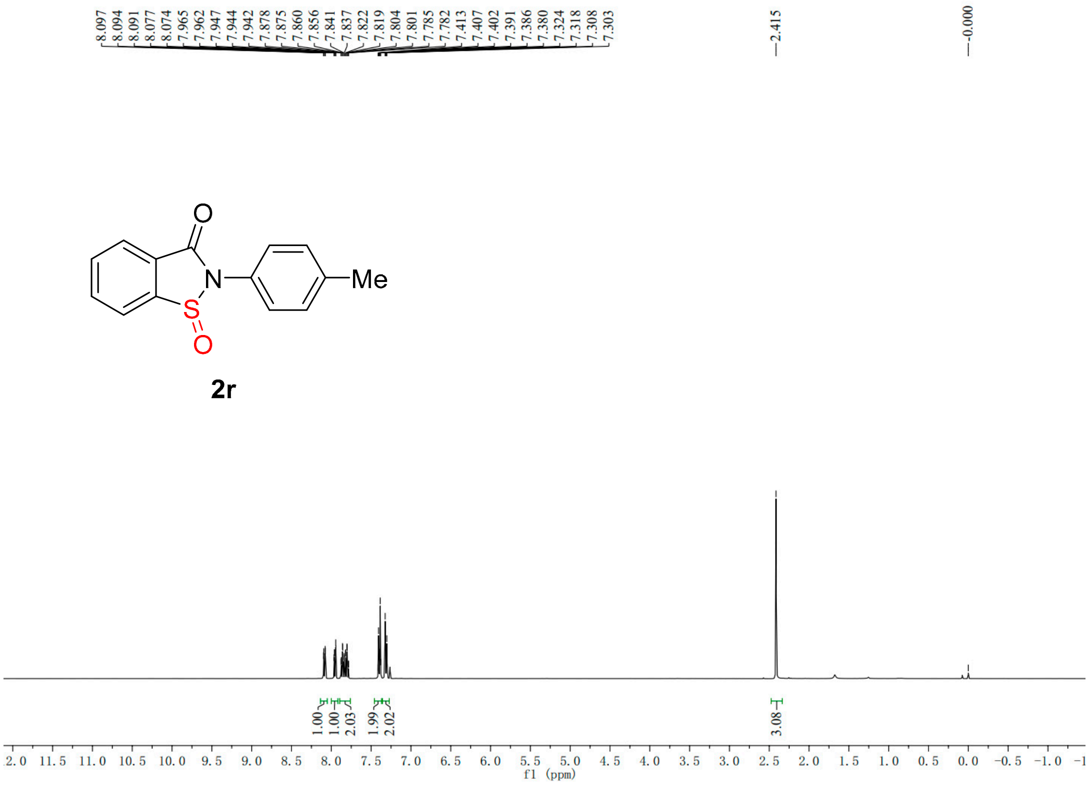

Figure S37 ¹H NMR spectrum (400MHz, $CDCl_3$, 298K) of **2r**

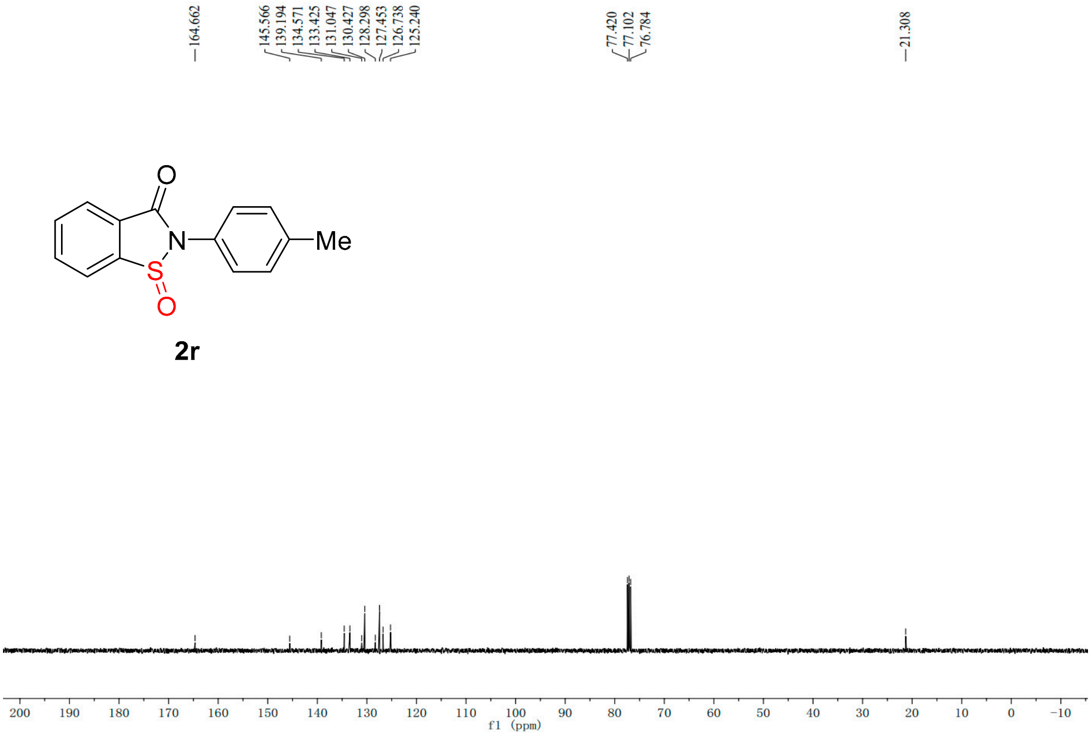

Figure S38 ¹³C NMR spectrum (101MHz, $CDCl_3$, 298K) of **2r**

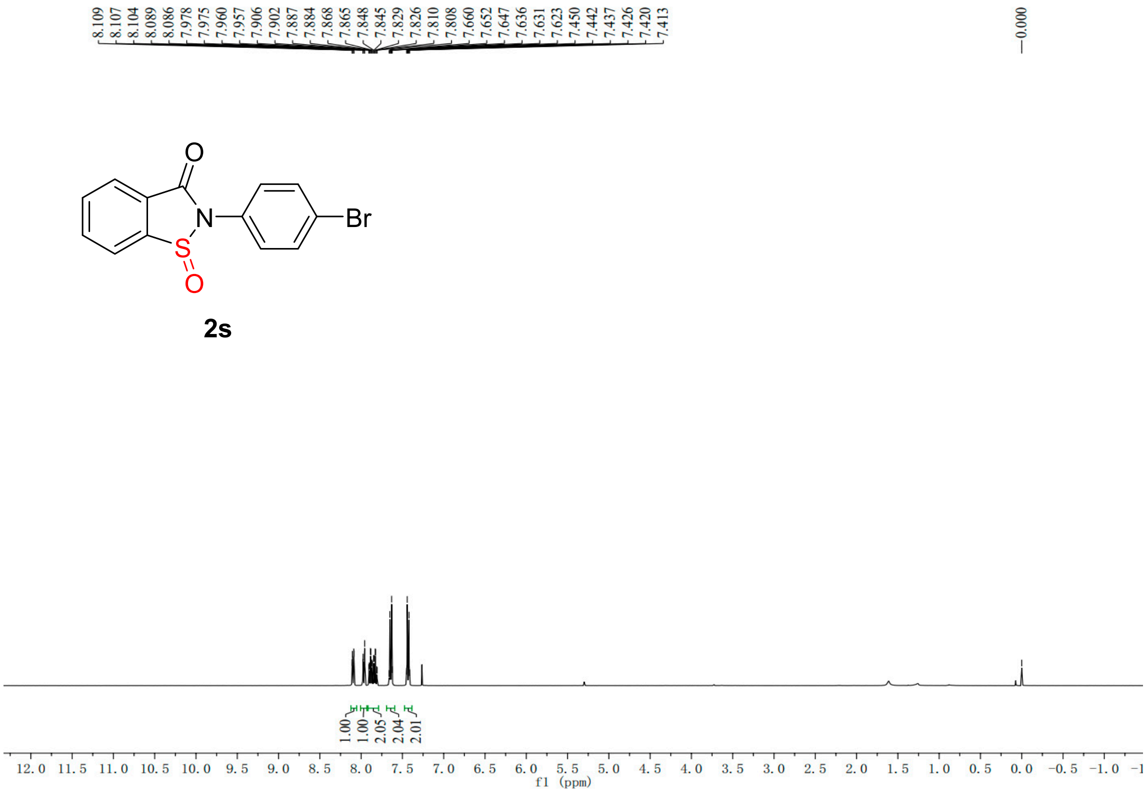

Figure S39 ^{1}H NMR spectrum (400MHz, $CDCl_3$, 298K) of **2s**

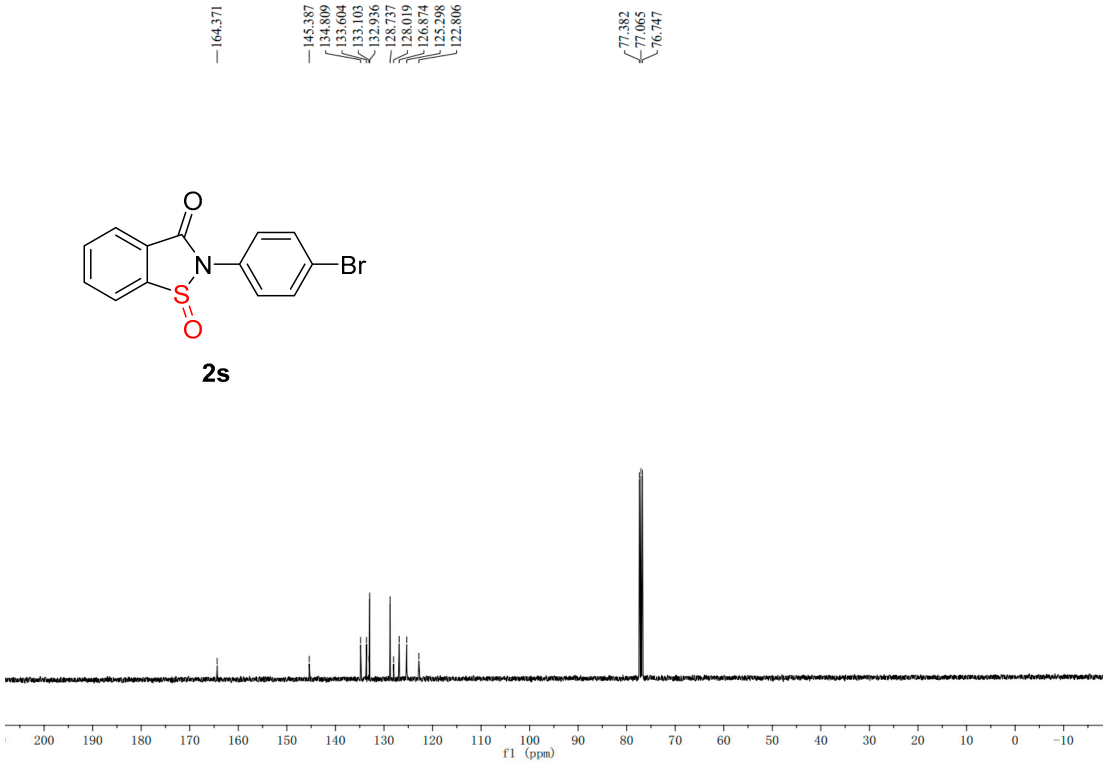

Figure S40 ^{13}C NMR spectrum (101MHz, $CDCl_3$, 298K) of **2s**

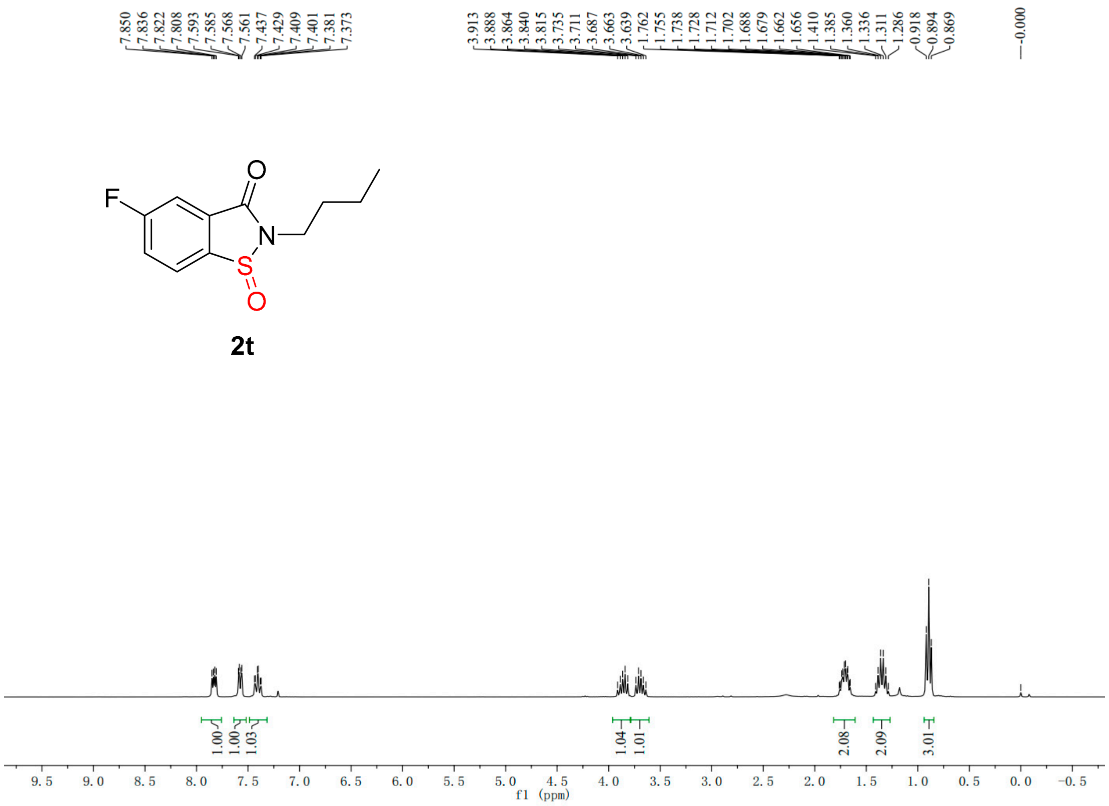

Figure S41 ^{1}H NMR spectrum (300MHz, $CDCl_3$, 298K) of **2t**

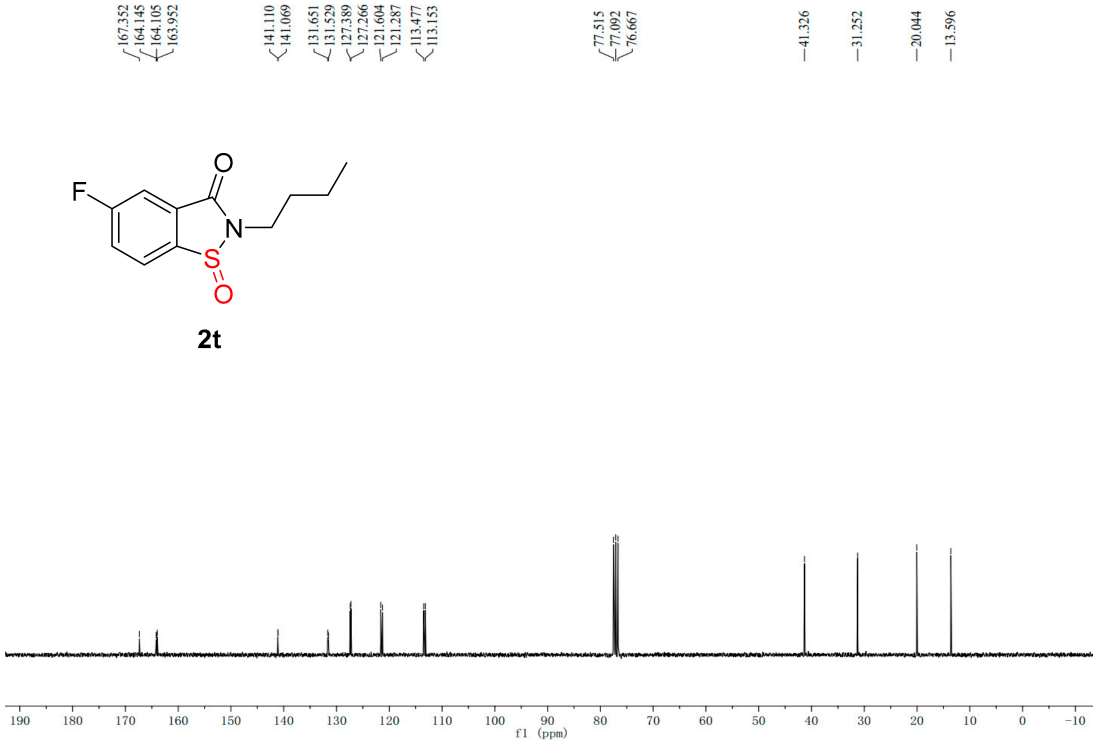

Figure S42 ^{13}C NMR spectrum (75MHz, $CDCl_3$, 298K) of **2t**

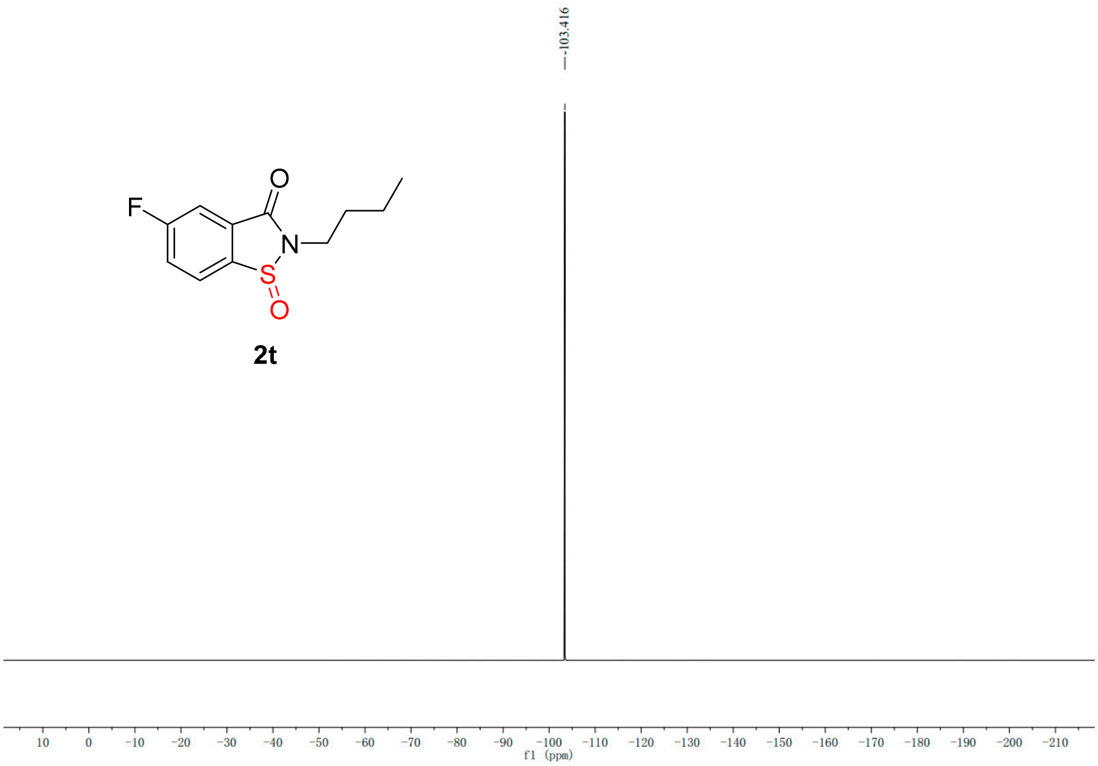

Figure S43 ^{19}F NMR spectrum (282MHz, $CDCl_3$, 298K) of **2t**

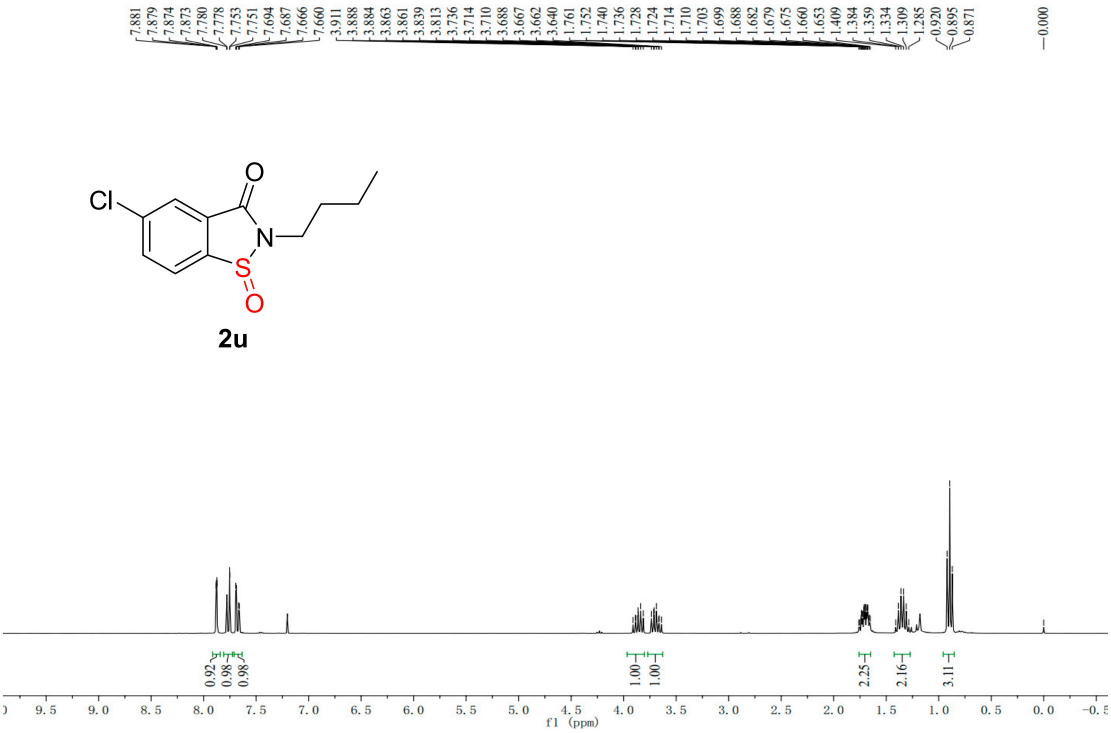

Figure S44 ^{1}H NMR spectrum (300MHz, $CDCl_3$, 298K) of **2u**

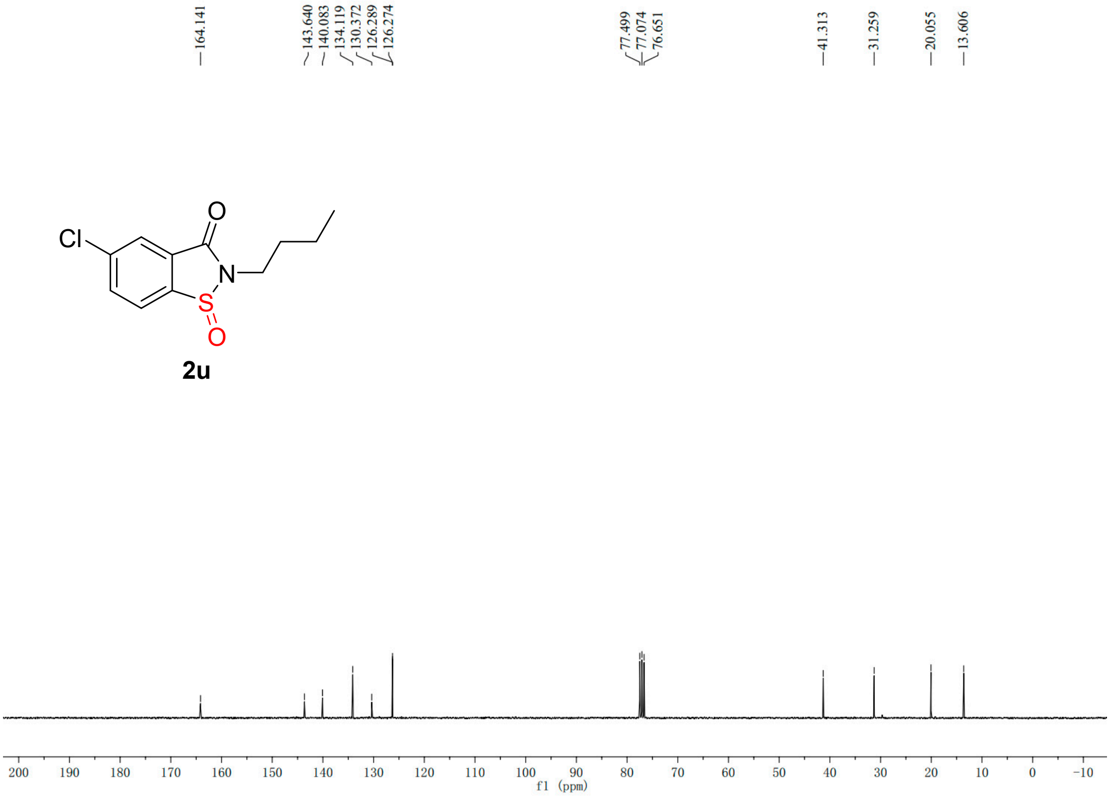

Figure S45 ^{13}C NMR spectrum (75MHz, $CDCl_3$, 298K) of **2u**

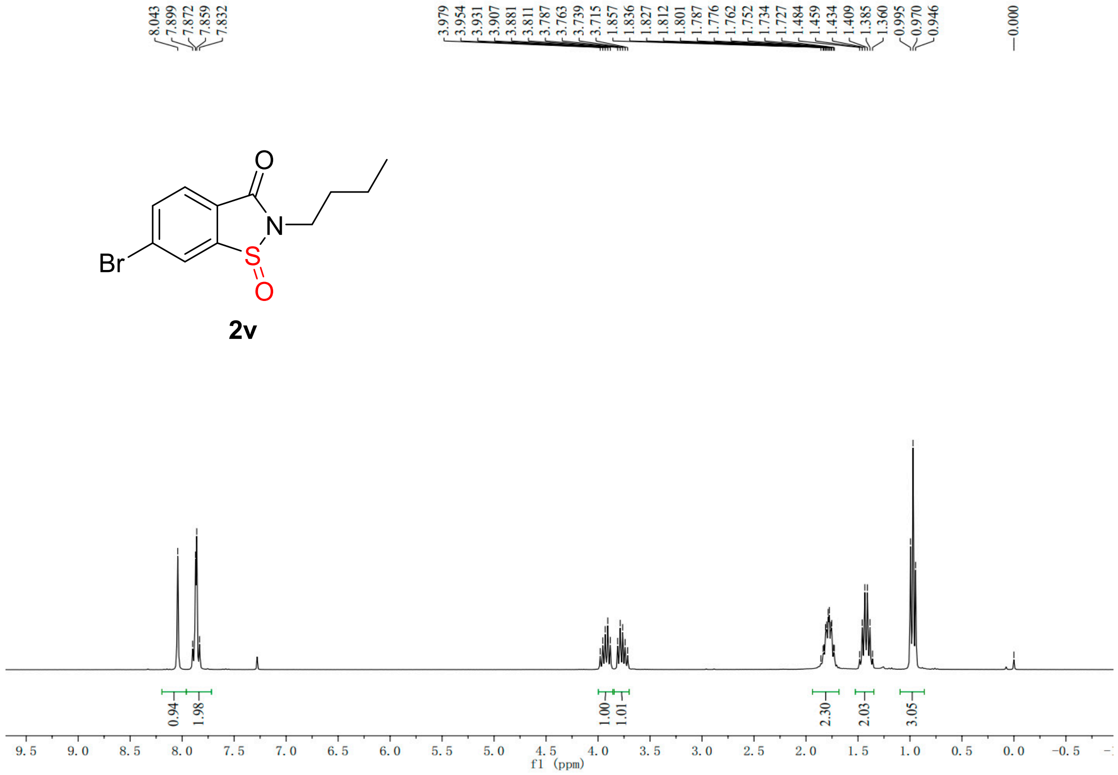

Figure S46 ^{1}H NMR spectrum (300MHz, $CDCl_3$, 298K) of **2v**

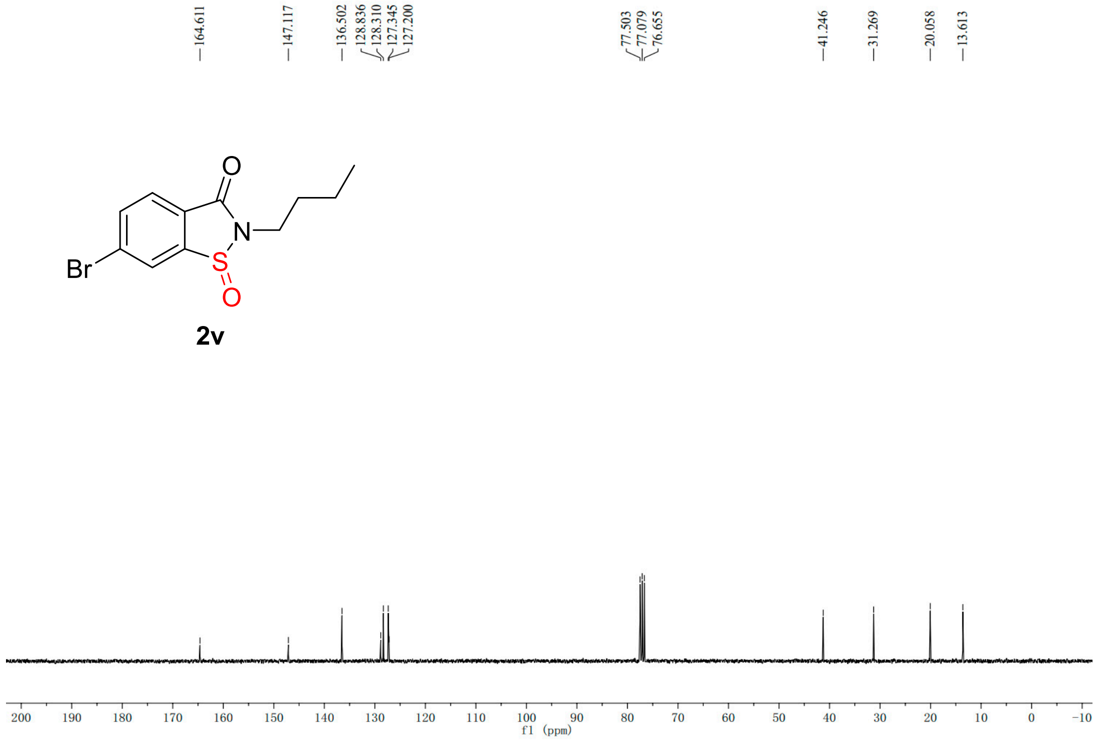

Figure S47 ^{13}C NMR spectrum (75MHz, $CDCl_3$, 298K) of **2v**

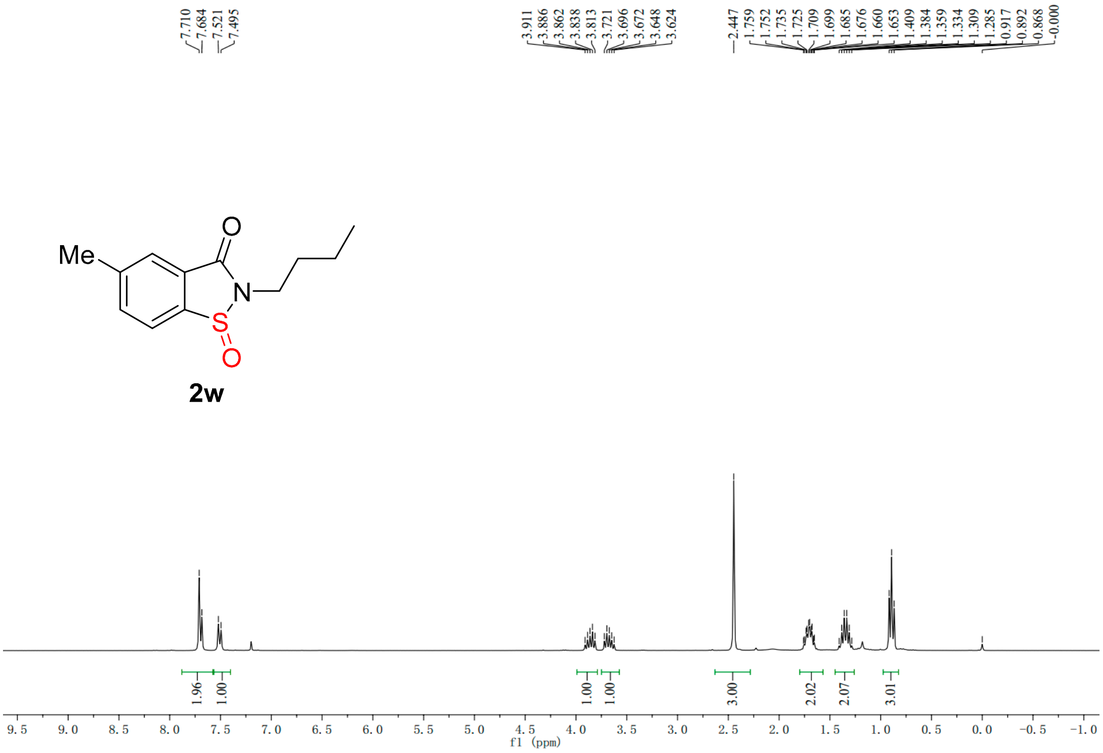

Figure S48 ^{1}H NMR spectrum (300MHz, $CDCl_3$, 298K) of **2w**

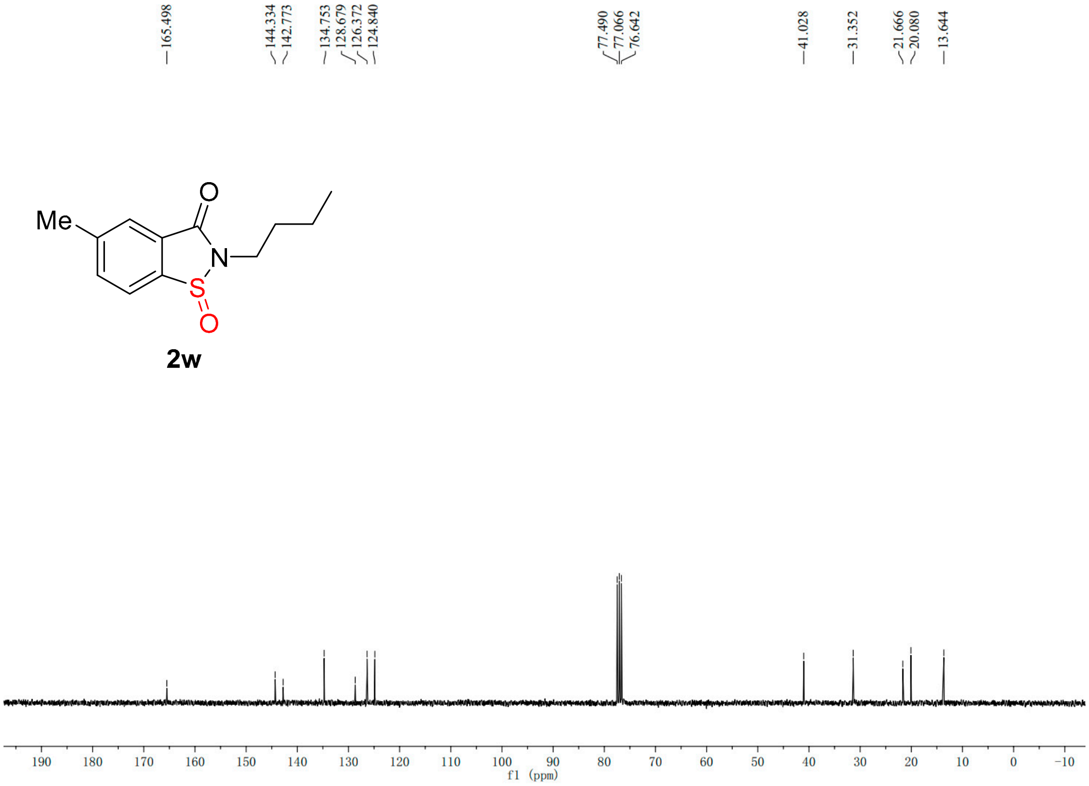

Figure S49 ^{13}C NMR spectrum (75MHz, $CDCl_3$, 298K) of **2w**

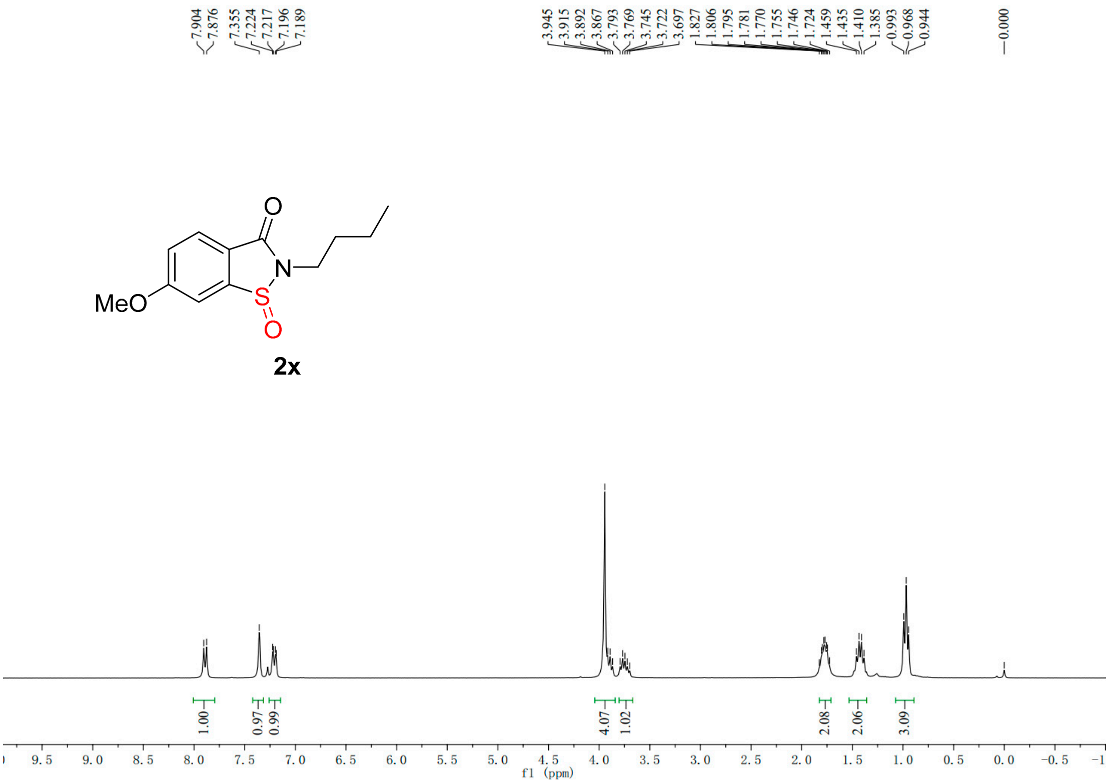

Figure S50 ¹H NMR spectrum (300MHz, CDCl₃, 298K) of **2x**

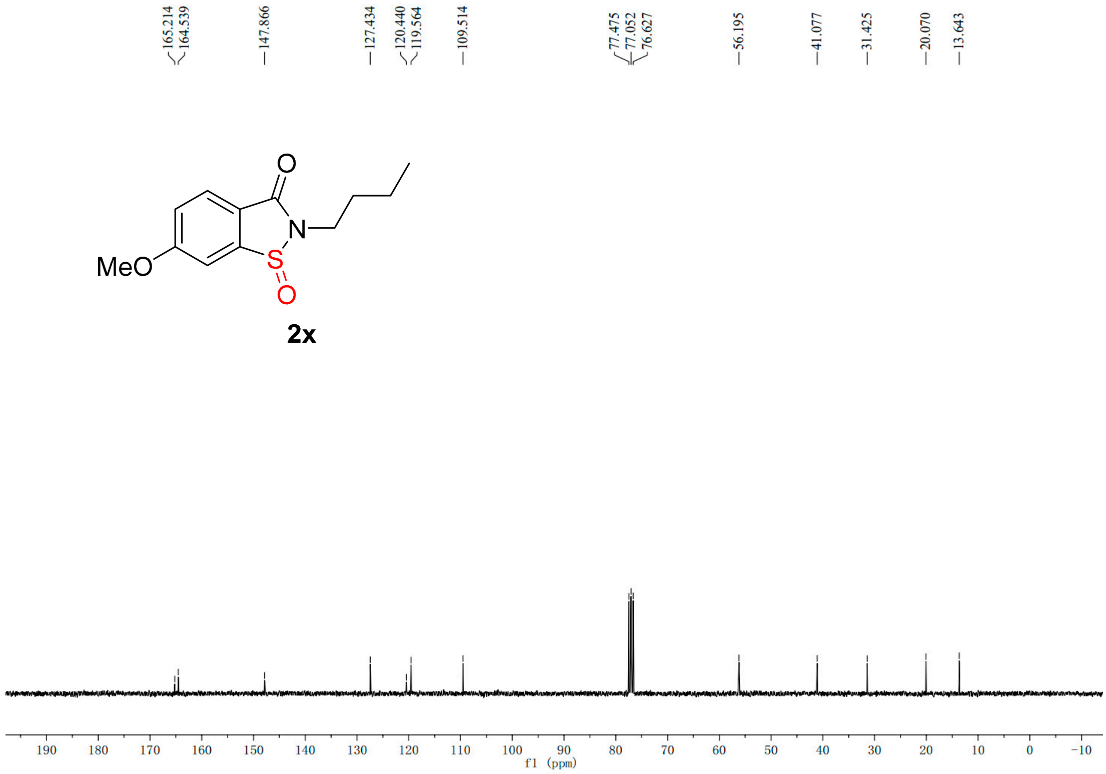

Figure S51 ¹³C NMR spectrum (75MHz, CDCl₃, 298K) of **2x**

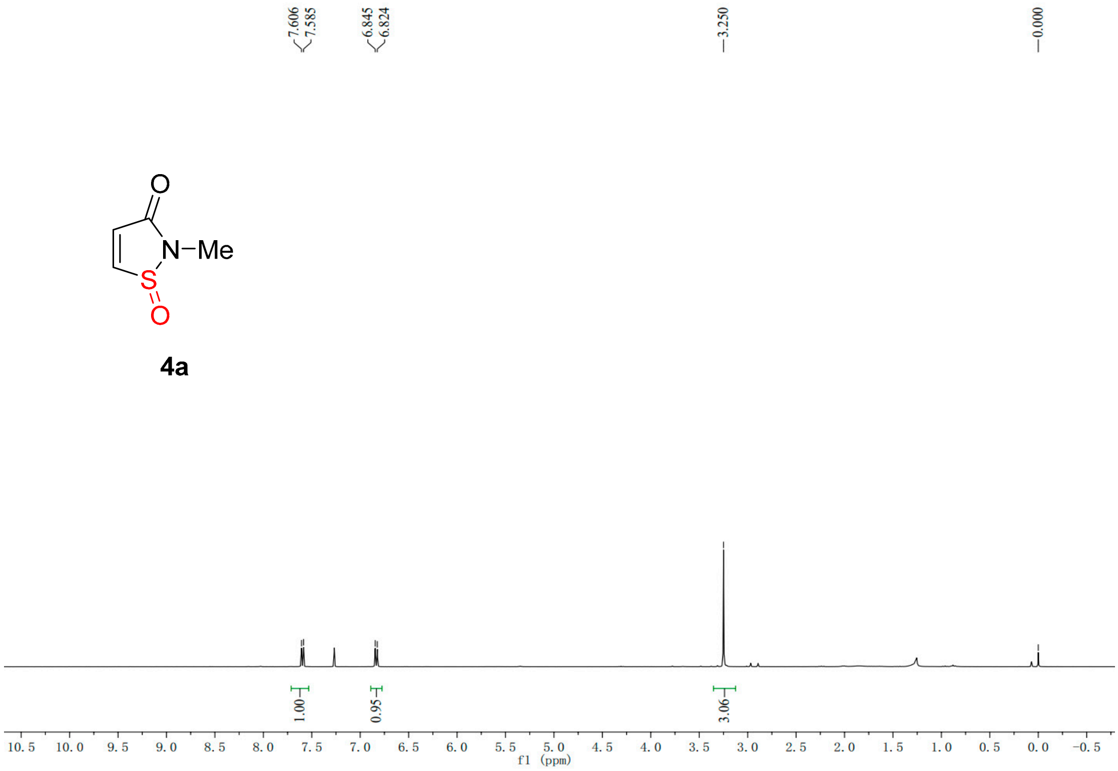

Figure S52 ^{1}H NMR spectrum (300MHz, $CDCl_3$, 298K) of **4a**

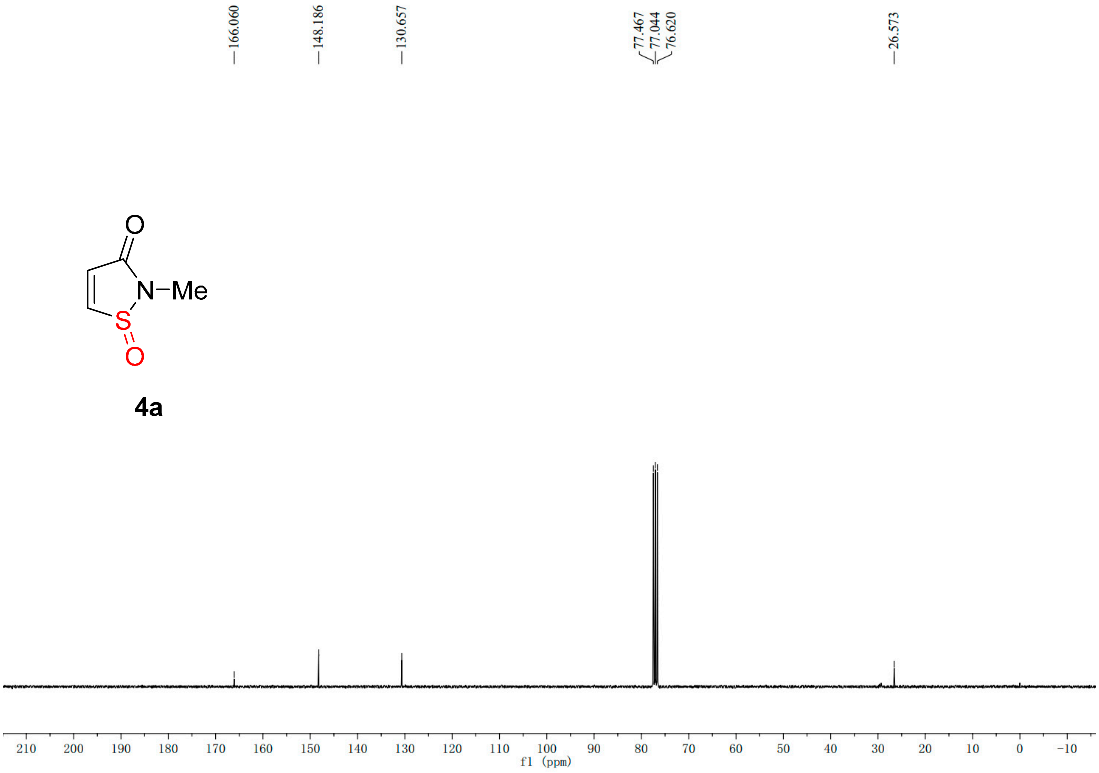

Figure S53 ^{13}C NMR spectrum (75MHz, $CDCl_3$, 298K) of **4a**

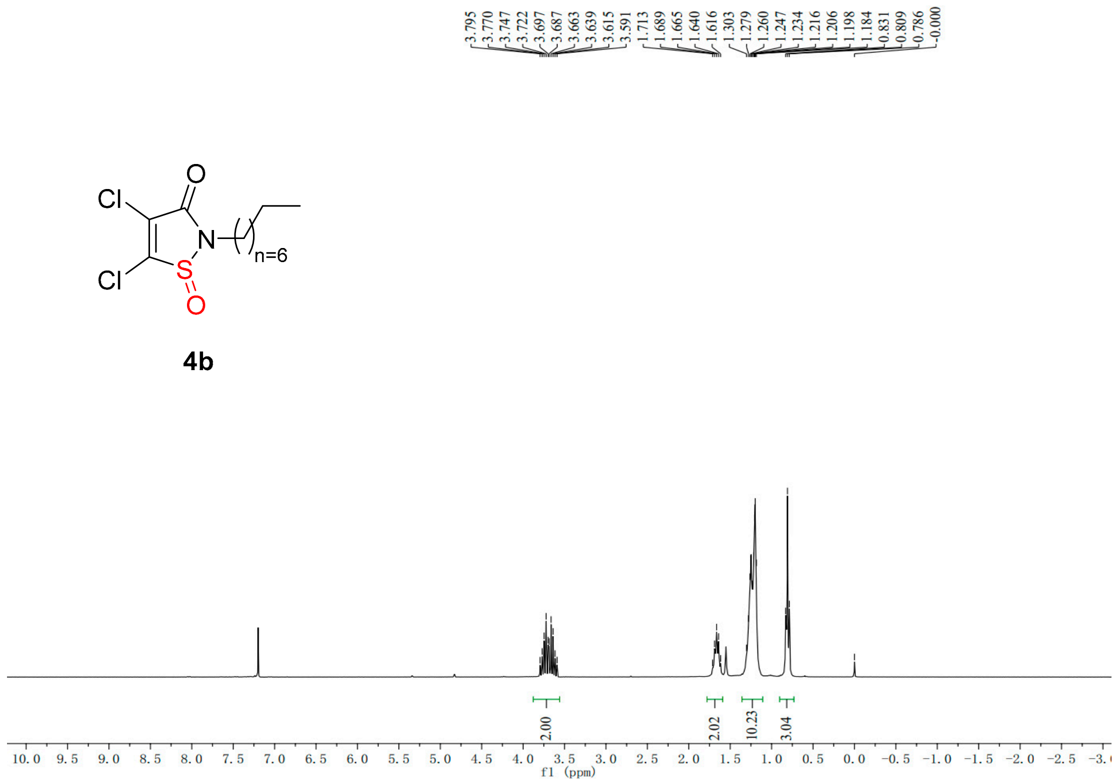

Figure S54 ^{1}H NMR spectrum (300MHz, $CDCl_3$, 298K) of **4b**

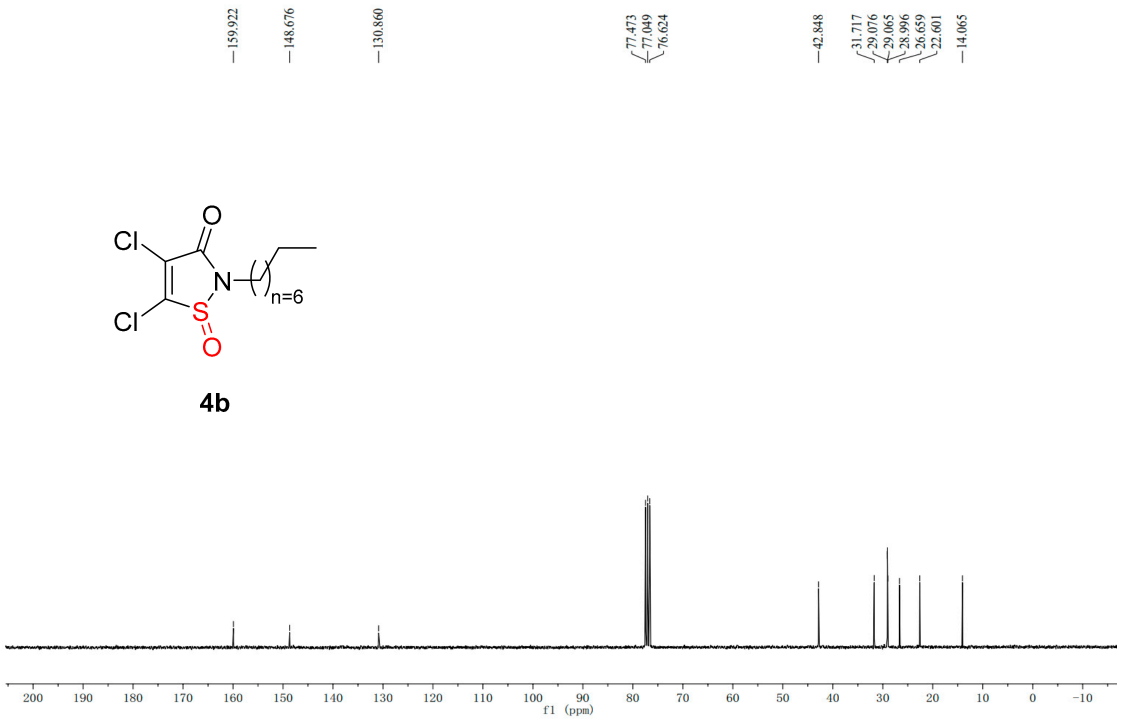

Figure S55 ^{13}C NMR spectrum (75MHz, $CDCl_3$, 298K) of **4b**

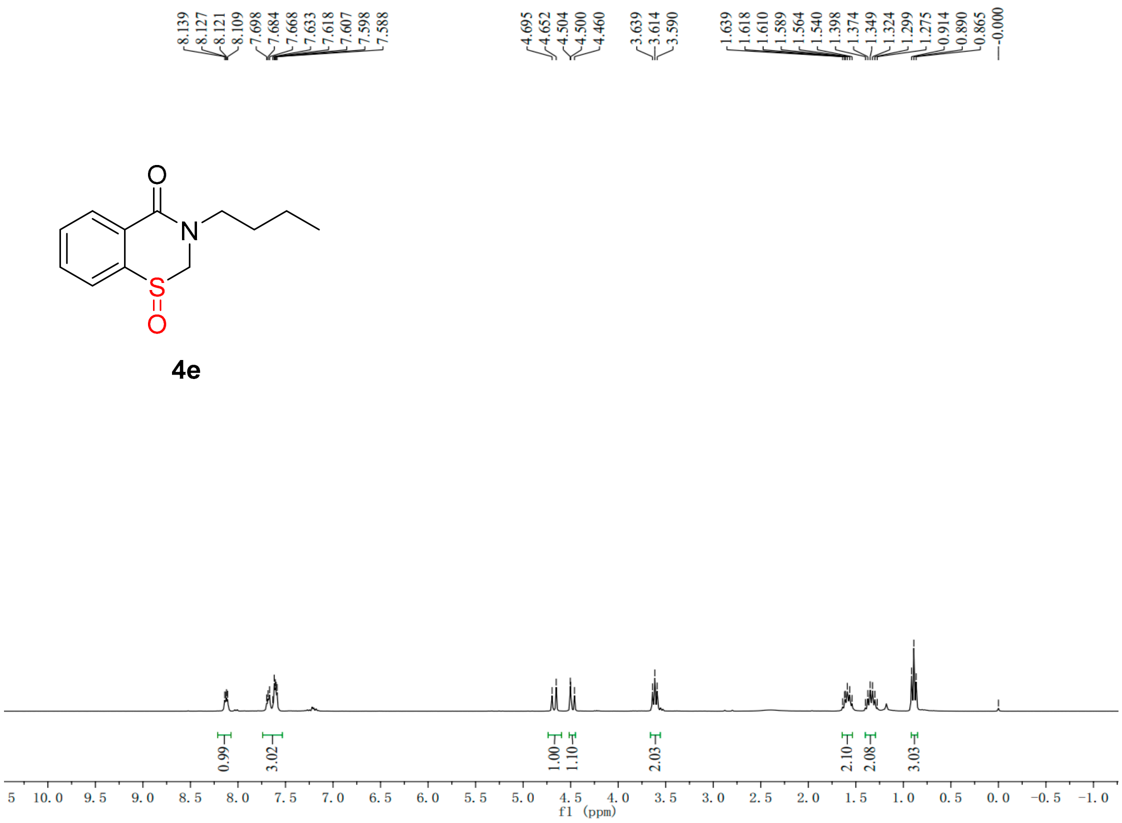

Figure S56 ^{1}H NMR spectrum (300MHz, $CDCl_3$, 298K) of **4e**

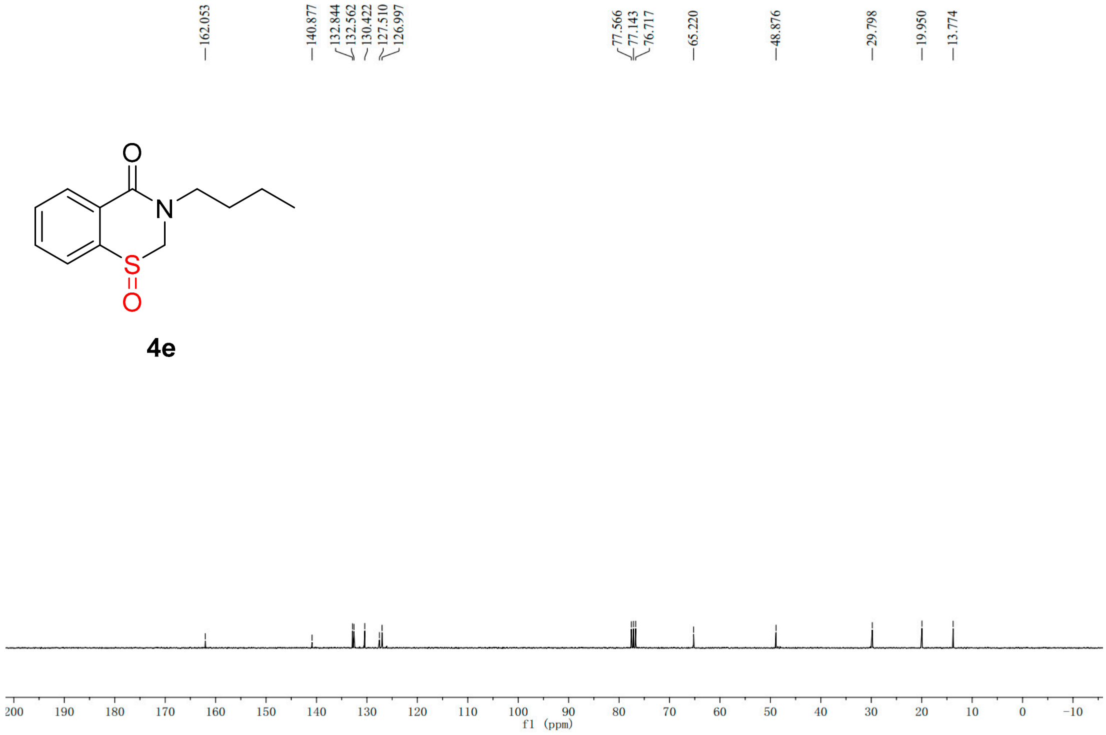

Figure S57 ^{13}C NMR spectrum (75MHz, $CDCl_3$, 298K) of **4e**

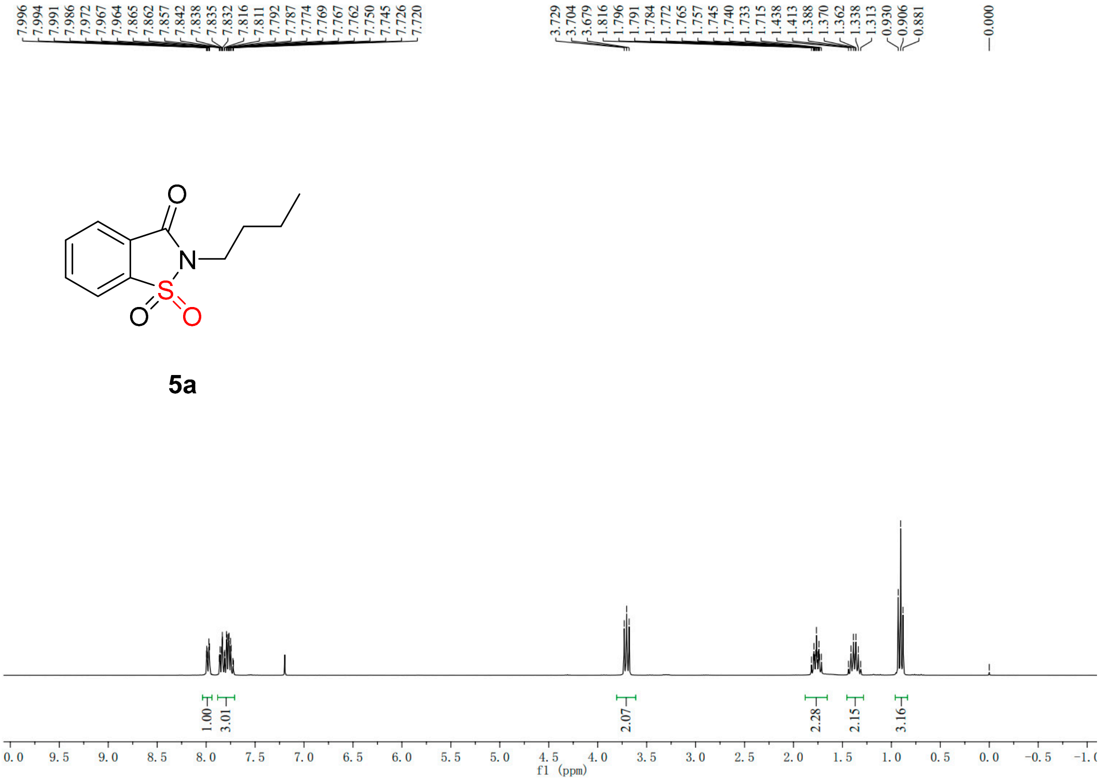

Figure S58 ^{1}H NMR spectrum (300MHz, $CDCl_3$, 298K) of **5a**

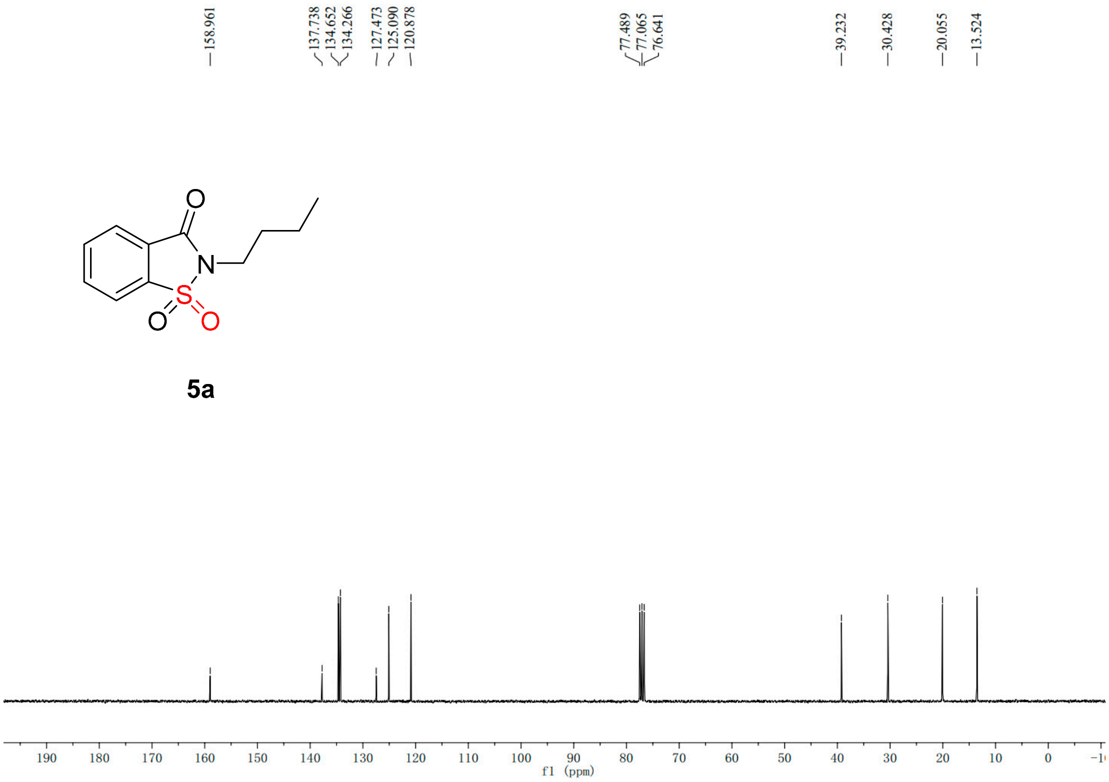

Figure S59 ^{13}C NMR spectrum (75MHz, $CDCl_3$, 298K) of **5a**

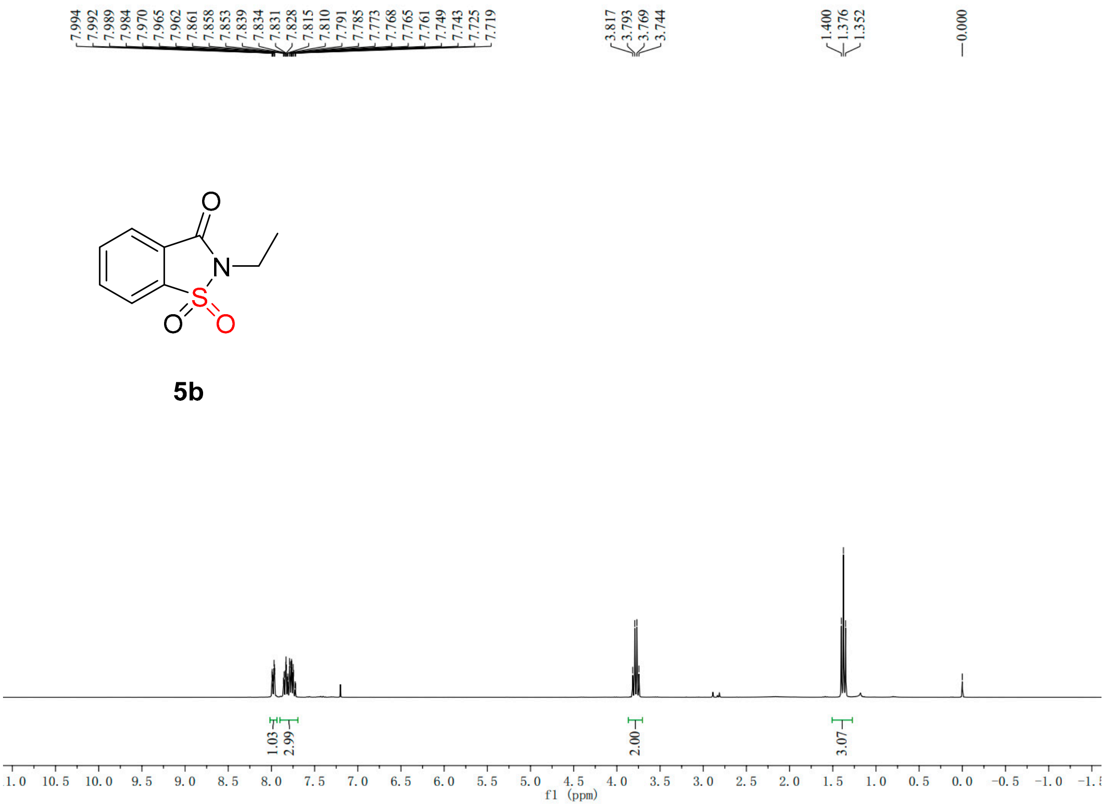

Figure S60 ^{1}H NMR spectrum (300MHz, $CDCl_3$, 298K) of **5b**

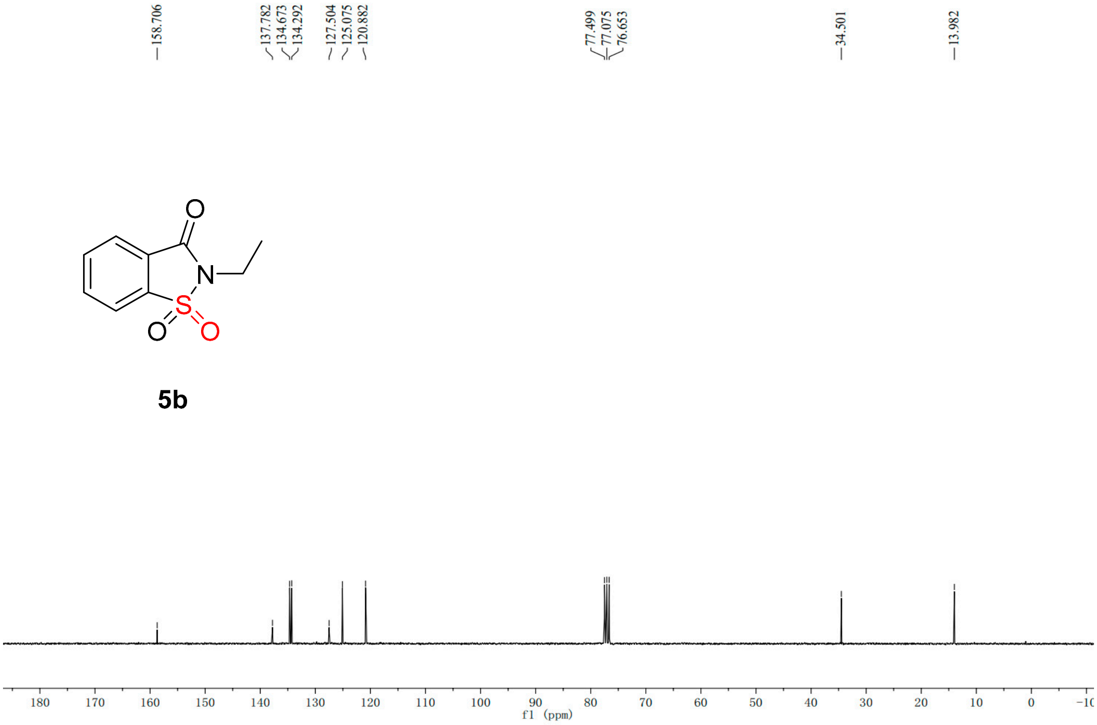

Figure S61 ^{13}C NMR spectrum (75MHz, $CDCl_3$, 298K) of **2h**

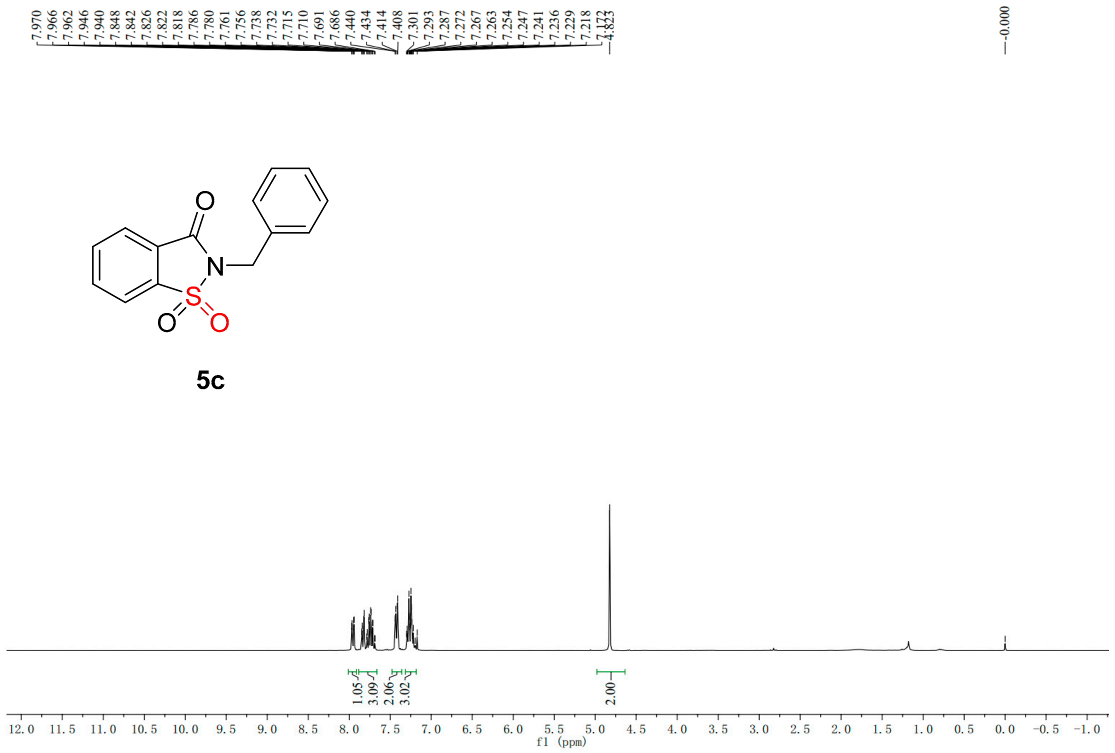

Figure S62 ^{1}H NMR spectrum (300MHz, $CDCl_3$, 298K) of **5c**

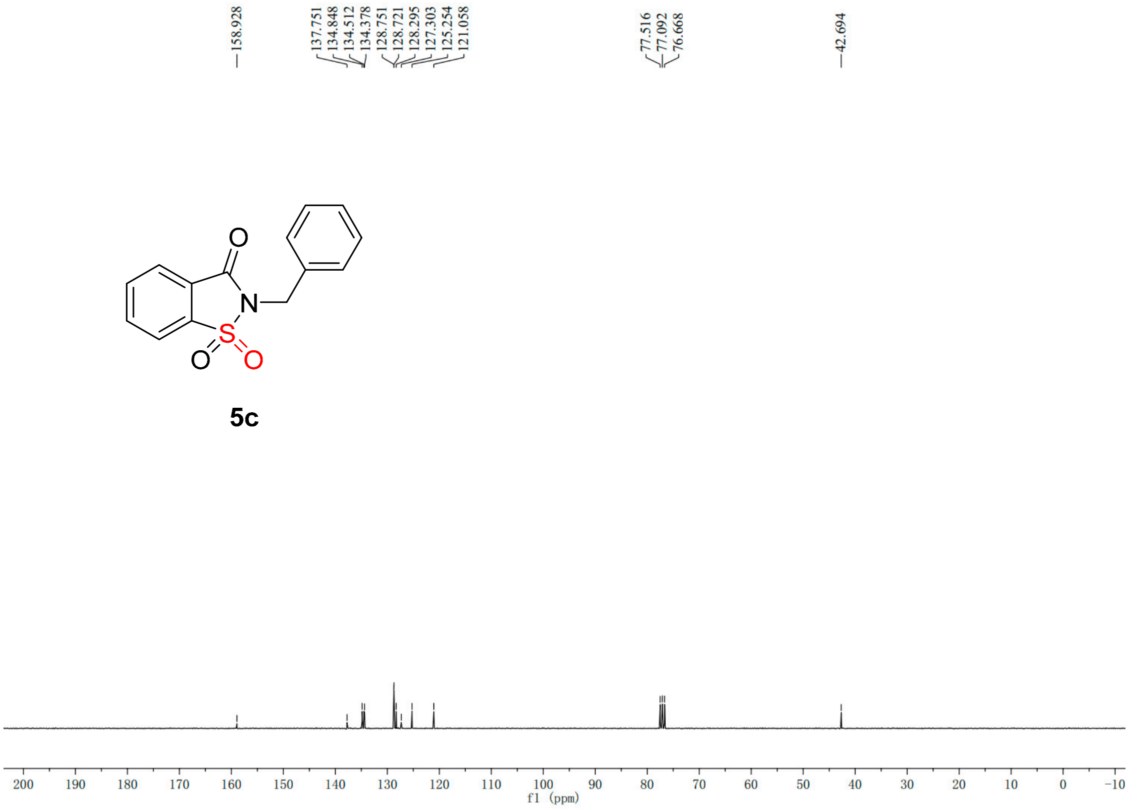

Figure S63 ^{13}C NMR spectrum (75MHz, $CDCl_3$, 298K) of **5c**

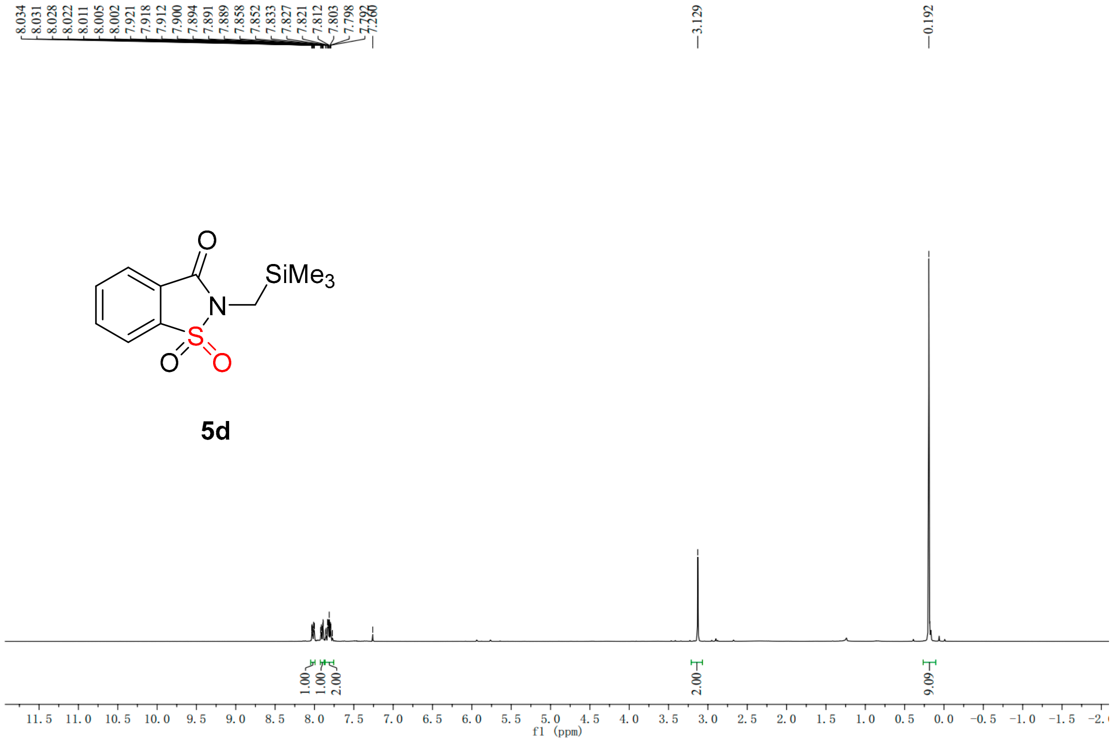

Figure S64 ^{1}H NMR spectrum (300MHz, $CDCl_3$, 298K) of **5d**

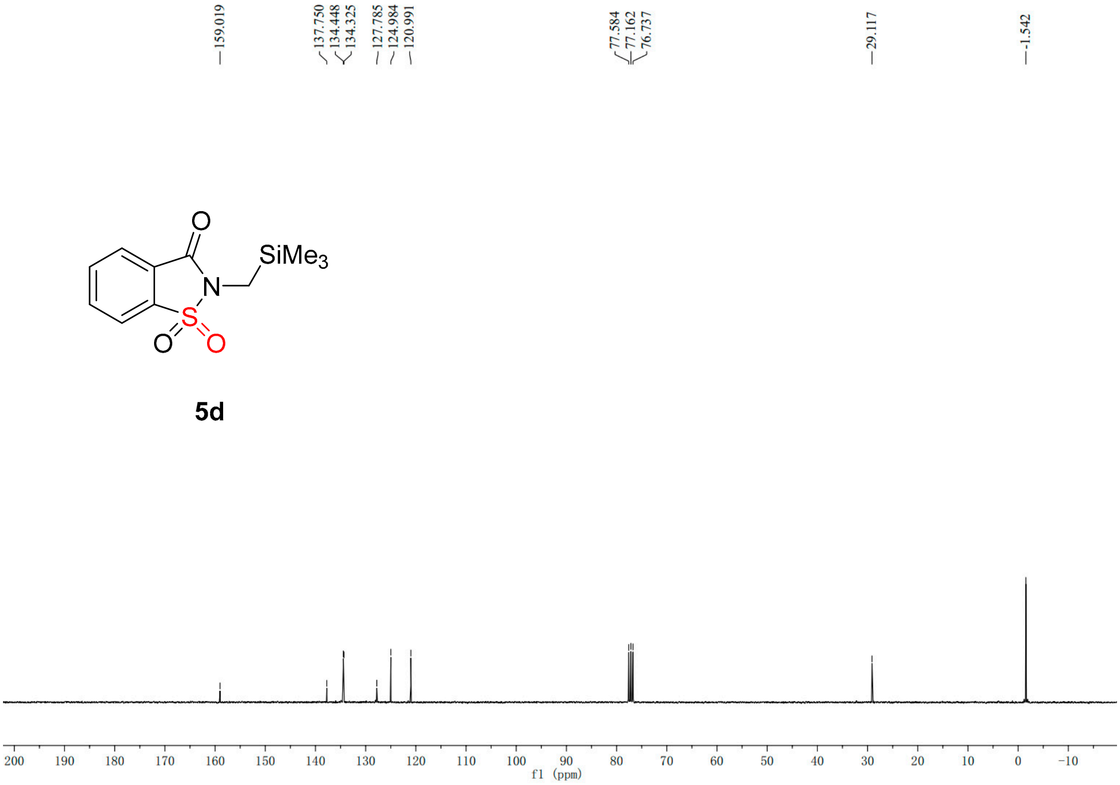

Figure S65 ^{13}C NMR spectrum (75MHz, $CDCl_3$, 298K) of **5d**

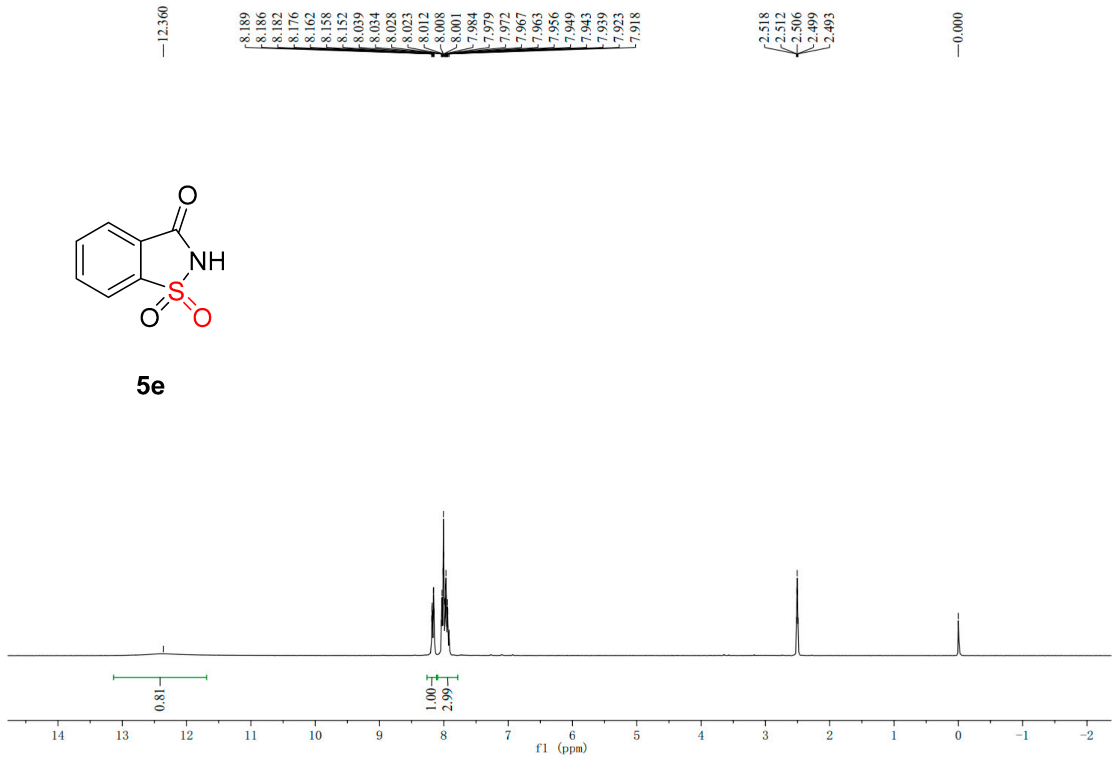

Figure S66 ¹H NMR spectrum (300MHz, DMSO, 298K) of **5e**

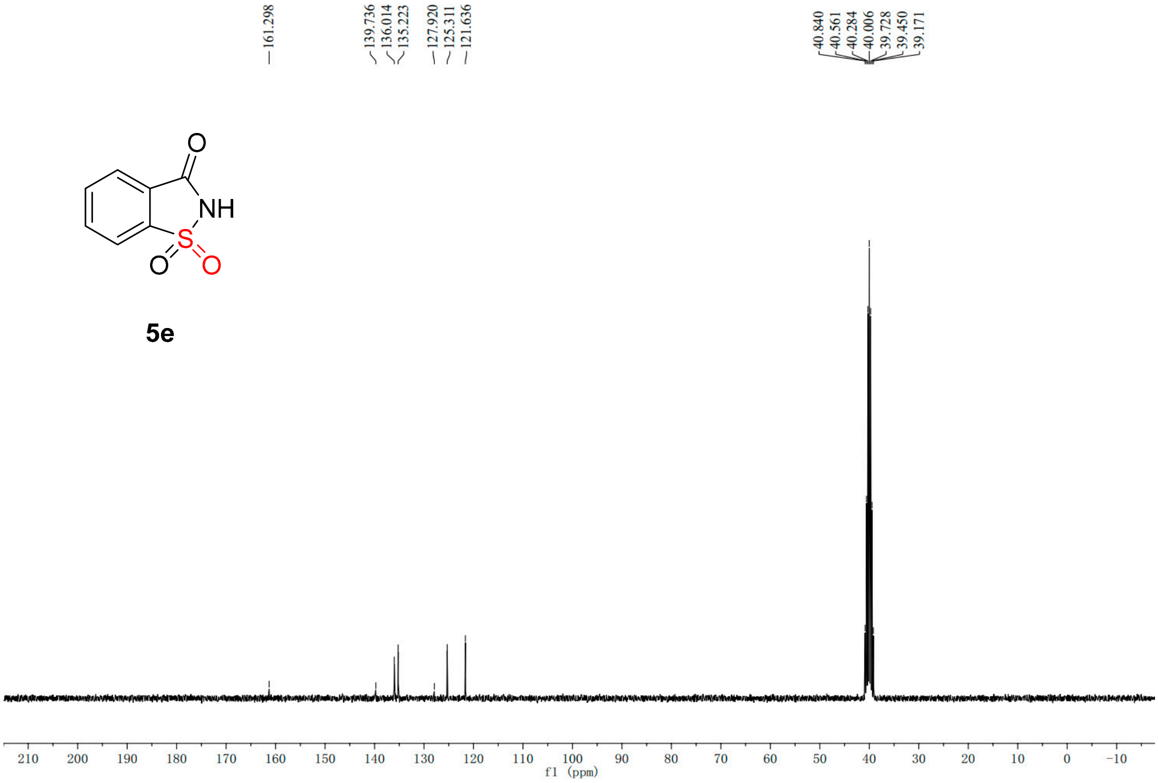

Figure S67 ¹³C NMR spectrum (75MHz, DMSO, 298K) of **5e**
